# *Euoplocephalus tutus* and the Diversity of Ankylosaurid Dinosaurs in the Late Cretaceous of Alberta, Canada, and Montana, USA

**DOI:** 10.1371/journal.pone.0062421

**Published:** 2013-05-08

**Authors:** Victoria M. Arbour, Philip J. Currie

**Affiliations:** Department of Biological Sciences, University of Alberta, Edmonton, Alberta, Canada; Raymond M. Alf Museum of Paleontology, United States of America

## Abstract

Few ankylosaurs are known from more than a single specimen, but the ankylosaurid *Euoplocephalus tutus* (from the Late Cretaceous of Alberta, Canada and Montana, USA) is represented by dozens of skulls and partial skeletons, and is therefore an important taxon for understanding intraspecific variation in ankylosaurs. *Euoplocephalus* is unusual compared to other dinosaurs from the Late Cretaceous of Alberta because it is recognized from the Dinosaur Park, Horseshoe Canyon, and Two Medicine formations. A comprehensive review of material attributed to *Euoplocephalus* finds support for the resurrection of its purported synonyms *Anodontosaurus lambei* and *Scolosaurus cutleri*, and the previously resurrected *Dyoplosaurus acutosquameus*. *Anodontosaurus* is found primarily in the Horseshoe Canyon Formation of Alberta and is characterized by ornamentation posterior to the orbits and on the first cervical half ring, and wide, triangular knob osteoderms. *Euoplocephalus* is primarily found in Megaherbivore Assemblage Zone 1 in the Dinosaur Park Formation of Alberta and is characterized by the absence of ornamentation posterior to the orbits and on the first cervical half ring, and keeled medial osteoderms on the first cervical half ring. *Scolosaurus* is found primarily in the Two Medicine Formation of Montana (although the holotype is from Dinosaur Provincial Park), and is characterized by long, back-swept squamosal horns, ornamentation posterior to the orbit, and low medial osteoderms on the first cervical half ring; *Oohkotokia horneri* is morphologically indistinguishable from *Scolosaurus cutleri*. *Dyoplosaurus* was previously differentiated from *Euoplocephalus* sensu lato by the morphology of the pelvis and pes, and these features also differentiate *Dyoplosaurus* from *Anodontosaurus* and *Scolosaurus*; a narrow tail club knob is probably also characteristic for *Dyoplosaurus*.

## Introduction

**Figure 1:**
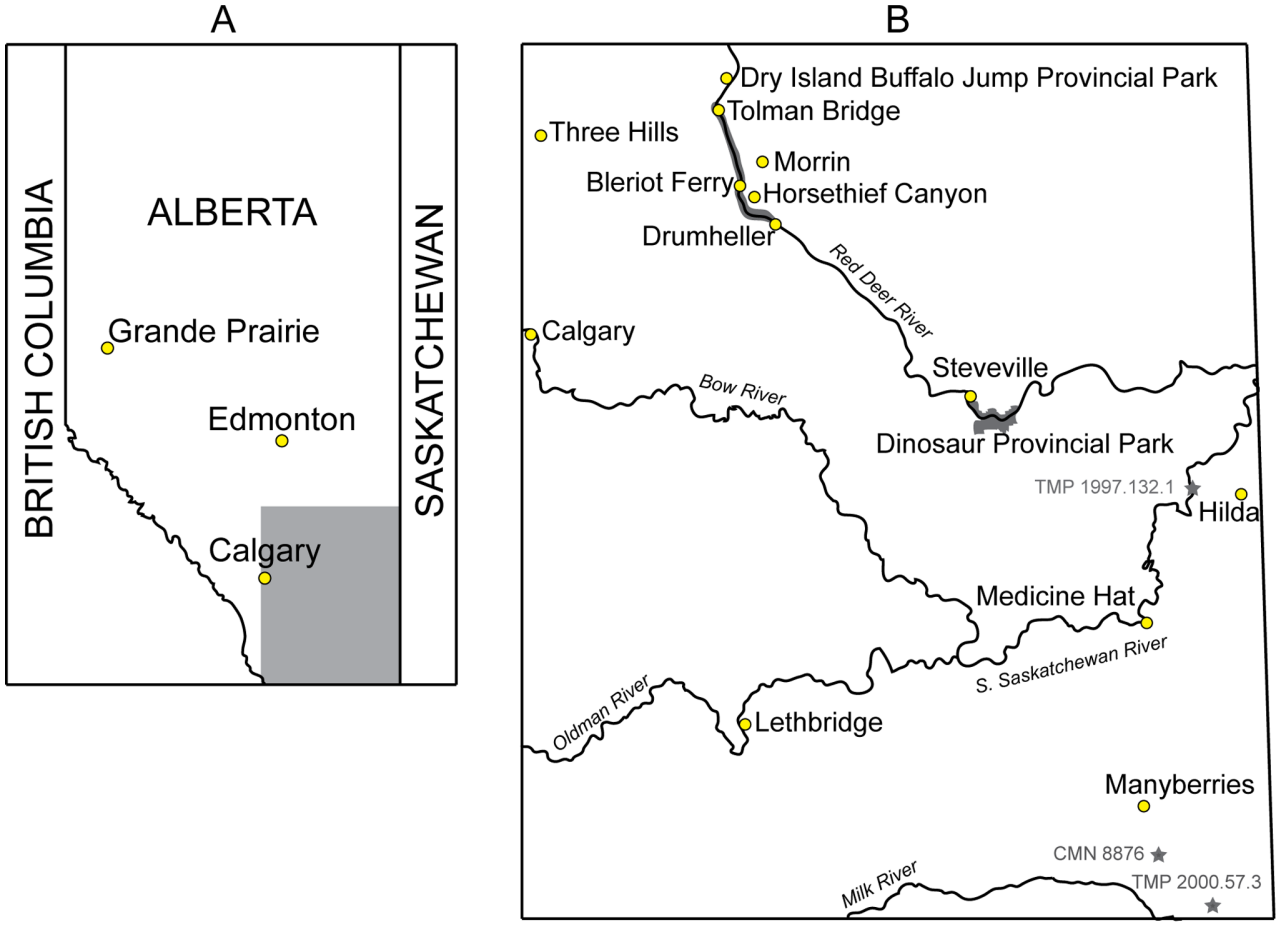
Geographic distribution of Albertan ankylosaurids. **A**) Map of the Canadian province of Alberta. **B**) Area represented by grey square in (**A**), showing locations of localities discussed in this paper. Specimens referred to *Euoplocephalus tutus* have been collected from sediments between Tolman Bridge and Drumheller, within Dinosaur Provincial Park, west of Hilda, and south of Manyberries.

More fossil material has been referred to *Euoplocephalus tutus* ( = *Stereocephalus tutus* Lambe, 1902 [1]) Lambe, 1910 [2], than to any other North American ankylosaurid to date. As such, this taxon features prominently in discussions of ankylosaurid anatomy, systematics, and paleobiology [3]–[17]. *Euoplocephalus tutus* is identified primarily from Alberta (Fig. 1), but a few referred specimens have been recovered from the Two Medicine and Judith River formations of Montana. Compared to most other dinosaurs from the Late Cretaceous of Alberta, specimens identified as *Euoplocephalus tutus* have an unusually long stratigraphic range, spanning both the Dinosaur Park and Horseshoe Canyon formations, from about 76 to 67 Ma. In contrast, species of nodosaurid ankylosaurs, ceratopsians, hadrosaurs, and tyrannosaurs all have relatively restricted stratigraphic ranges within the Dinosaur Park Formation [18].

Understanding variation in *Euoplocephalus tutus* is important for two reasons. First, the number of taxa represented by specimens referred to *Euoplocephalus tutus* has important implications for understanding biogeographic and biostratigraphic patterns of dinosaur diversity in the Upper Cretaceous of North America; either *Euoplocephalus tutus* differs from other Albertan ornithischian genera in having an unusually long stratigraphic range, or ankylosaurid diversity in Alberta is greater than generally thought. Second, variation in *Euoplocephalus tutus* (one of only a few ankylosaurid taxa represented by a reasonably large sample size) could provide support for (or against) morphological features used to diagnose other ankylosaurid taxa by clarifying which features are most likely to result from intraspecific variation.

Coombs [7] synonymized several taxa with *Euoplocephalus tutus*: *Anodontosaurus lambei* Sternberg, 1929 [19], *Dyoplosaurus acutosquameus* Parks, 1924 [20], and *Scolosaurus cutleri* Nopcsa, 1928 [21]. Lambe [1] named *Euoplocephalus tutus* (as *Stereocephalus tutus*) on the basis of CMN 0210 (institutional abbreviations in Table 1), a fragmentary skull roof (Fig. S1), partial first cervical half ring, and rib (Vickaryous and Russell [16] note that an unprepared right mandible is associated with this specimen). Lambe [1] also referred a tooth and two large osteoderm spikes to *Euoplocephalus tutus*, but provided no specimen numbers. Vickaryous and Russell [16] provided specimen numbers for the rib fragment (CMN 1463), tooth (CMN 1772), and large spiked osteoderms (CMN 0317, CMN 0608), and suggested that the tooth belonged to a nodosaurid ankylosaur. The figured osteoderm spike (CMN 0317) appears to belong to a nodosaurid ankylosaur such as *Edmontonia* (e.g. AMNH 5665, USNM 11868); no ankylosaurid is known to possess a solid, narrow, conical spike such as CMN 0317. Lambe [1] referred *Euoplocephalus tutus* to the Stegosauridae on the basis of T-shaped rib cross-sections and noted that the skull was unlike any dinosaur described up to that time. Although Lambe [1] did not explicitly state any diagnostic characters, the cranial ornamentation pattern and first cervical half ring would have been unknown in any other dinosaur at the time. In fact, Lambe [1] interpreted the cervical half ring as perhaps belonging to the posterior border of a cranial crest. A second, better preserved skull (UALVP 31; Fig. S2) was referred to *Euoplocephalus tutus* (although incorrectly called “*Europlocephalus*” *tutus* throughout) by Gilmore [22], based on the shape and arrangement of the cranial ornamentation.

**Table 1:** Institutional abbreviations and locations.

Abbreviation	Institution	Location
AMNH	American Museum of Natural History	New York, New York, USA
CMN	Canadian Museum of Nature	Ottawa, Ontario, Canada
FPDM-V	Fukui Prefectural Dinosaur Museum (Vertebrate Collection)	Katsuyama, Fukui Prefecture, Japan
NHMUK	Natural History Museum	London, U.K.
MACN Pv	Colección nacional de Paleontología de Vertebrados del Museo Argentino de Ciencias Naturales “Bernardino Rivadavia”	Buenos Aires, Argentina
MOR	Museum of the Rockies	Bozeman, Montana, USA
MPC	Mongolian Paleontological Center	Ulaanbaatar, Mongolia
NSM PV	National Museum of Nature and Science	Tokyo, Japan
PIN	Paleontological Institute, Russian Academy of Sciences	Moscow, Russia
ROM	Royal Ontario Museum	Toronto, Ontario, Canada
TMP	Royal Tyrrell Museum of Palaeontology	Drumheller, Alberta, Canada
UALVP	University of Alberta Laboratory for Vertebrate Paleontology	Edmonton, Alberta, Canada
USNM	Smithsonian National Museum of Natural History	Washington, DC, USA
ZPAL	Zoological Institute of Paleobiology, Polish Academy of Sciences	Warsaw, Poland

The holotype of *Dyoplosaurus acutosquameus* [20] (ROM 784), includes a fragmentary skull (Fig. S3), a partial pelvis, a well preserved caudal series including the tail club and ossified tendons, and forelimb and hindlimb elements. This was the first description of the unique ankylosaurid tail club in the scientific literature. Parks [20] noted that the fragmentary skull was unsatisfactory for comparison with *Euoplocephalus tutus*, but observed that the cranial ornamentation in ROM 784 differed from that of *Euoplocephalus tutus*.

The holotype of *Scolosaurus cutleri* [21] is a remarkable specimen that preserves nearly the entire skeleton as well as *in situ* osteoderms and skin impressions, but lacks the skull and distal half of the tail. Nopcsa [21] made numerous comparisons with *Dyoplosaurus acutosquameus* in his description of *Scolosaurus cutleri*, but because at the time only the skull and first cervical ring of *Euoplocephalus tutus* were known, and because *Scolosaurus cutleri* lacks a skull, no comparisons were made with *Euoplocephalus tutus*.

*Anodontosaurus lambei* [19] includes a skull and left mandible (Fig. S4), caudal vertebra, phalanx, and osteoderms. Sternberg [19] listed several diagnostic features of *Anodontosaurus lambei*, including the absence of teeth (and the development of ‘bony plates’ on the maxilla and dentary instead), a reduced mandible, dorsoventrally flattened skull, and thin-walled osteoderms. Sternberg [19] acknowledged that the skull of *Anodontosaurus lambei* was similar to that of UALVP 31 (*Euoplocephalus tutus*), but noted that *Anodontosaurus lambei* lacked the large central nasal ornamentation present in *Euoplocephalus tutus*.

Several ankylosaurid specimens from the Two Medicine Formation of Montana have also been referred to *Euoplocephalus* and its synonyms. Gilmore [23] described USNM 11892, a partial, crushed skull (Fig. S5), and referred it to *Dyoplosaurus* on the basis of similar tooth morphology. This specimen was later referred to *Euoplocephalus* by Coombs [7], who considered *Dyoplosaurus* as a junior synonym. Arbour et al. [24] did not reclassify USNM 11892 as *Dyoplosaurus* in their revision of that genus. Penkalski [13] described MOR 433, which includes a skull (Fig. S6) and partial postcranium, in a review of variation in *Euoplocephalus*; differences between MOR 433 and other *Euoplocephalus* specimens prompted Penkalski [13] to consider MOR 433 a distinct taxon, but no new name was erected at that time. Most recently, MOR 433 has been assigned as the holotype specimen of *Oohkotokia horneri* Penkalski, 2013 [25]. *Oohkotokia* includes all diagnostic ankylosaurid material from the Two Medicine Formation of Montana.

*Anodontosaurus lambei*, *Dyoplosaurus acutosquameus*, and *Scolosaurus cutleri* were synonymized with *Euoplocephalus tutus* by Coombs [7], but he did not formally rediagnose *Euoplocephalus tutus* or provide any justification for these synonymies. In his Ph.D. thesis, Coombs [4] explained his reasoning for these synonymies, and provided a diagnosis for *Euoplocephalus*. *Euoplocephalus tutus*, however, was not diagnosed, because Coombs [4] could find no characters separating it from the Mongolian species *Euoplocephalus giganteus* (previously *Dyoplosaurus giganteus* Maleev, 1956 [26], and currently accepted as *Tarchia gigantea* by Maryańska [27]). Coombs [4] noted that variation in skull size and overall shape, squamosal and quadratojugal horn sizes and shapes, and cranial ornamentation pattern could not split Judithian/Edmontonian ankylosaurids into subgroups; so either each skull must represent a distinct species, or all of the skulls must represent one species (*Euoplocephalus tutus*). Although no skull was known for *Dyoplosaurus acutosquameus* or *Scolosaurus cutleri*, Coombs [4] reasoned that if only one ankylosaurid species was valid in the Campanian of North America, then these two species must be junior synonyms of *Euoplocephalus tutus*. Coombs maintained the synonymy of *Anodontosaurus lambei*, *Dyoplosaurus acutosquameus*, and *Scolosaurus cutleri* with *Euoplocephalus tutus* throughout his publications on ankylosaurid anatomy [5]–[10]. Features considered diagnostic of *Euoplocephalus tutus* by Coombs [4] included premaxillae that are not covered by expanded nasals, long and slit-like nostrils, a premaxillary width that is equal or greater than the width between the most posterior maxillary teeth, a palate that does not taper anteriorly, and squamosal horns that are less prominent than those in *Ankylosaurus magniventris* Brown, 1908 [28].

Although Parks [20] presented skeletal and life restorations of the preserved material of *Dyoplosaurus acutosquameus*, the first attempt to restore the skeleton and life appearance of *Euoplocephalus tutus* was by Carpenter [3]. Carpenter [3] accepted the synonymy of *Anodontosaurus lambei*, *Dyoplosaurus acutosquameus*, and *Scolosaurus cutleri* with *Euoplocephalus tutus*. In particular, he noted the similarity between the skulls of *Anodontosaurus lambei* and *Euoplocephalus tutus*, but also noted that the cervical half ring of *Anodontosaurus lambei* was more similar to that of *Scolosaurus cutleri* than to that of *Euoplocephalus tutus*.

Penkalski [13] documented variation among the skulls and postcranial elements of *Euoplocephalus tutus*. A morphometric analysis of skull proportions did not yield discrete clusters of skulls, but did suggest that certain features (squamosal horn height, supraorbital ornamentation, location of apex of quadratojugal horn, and textures of cranial ornamentation) may be associated with overall skull size. Cervical half ring morphology was divided into two categories based on the number of osteoderms fused to the underlying band of bone [13]. Other features were more difficult to cluster, partly because of the lack of overlapping material among many specimens referred to *Euoplocephalus*. Although Penkalski [13] did not formally resurrect any of the synonymized taxa, he did strongly suggest that *Scolosaurus cutleri* was distinct from *Euoplocephalus tutus*.

Vickaryous and Russell [16] described and figured two new skulls (TMP 1991.127.1, Fig. S7, and TMP 1997.132.1, Fig. S8) from the Dinosaur Park Formation, and provided a revised diagnosis of the cranium for *Euoplocephalus tutus*. New diagnostic features included the presence of a ciliary osteoderm (referred to as a modified palpebral by Vickaryous and Russell [16], but see [29]), a shallow nasal vestibule, a vertical process of the premaxilla forming an intranarial septum (also present in *Tsagantegia longicranialis* Tumanova, 1993 [30]), and medially convergent, anteriorly and posteriorly divergent maxillary tooth rows. Vickaryous and Russell [16] supported the synonymy of *Anodontosaurus lambei* with *Euoplocephalus tutus*, finding no significant morphological differences between the holotype of *Anodontosaurus lambei,* the holotype of *Euoplocephalus tutus,* and referred *Euoplocephalus tutus* specimens. They suggested that many of the differences among *Euoplocephalus tutus* specimens can be attributed to taphonomic deformation, a hypothesis largely supported by Arbour and Currie [31].

Arbour et al. [24] reassessed the holotype specimen of *Dyoplosaurus acutosquameus* (ROM 784) and concluded that this represented a distinct species from *Euoplocephalus tutus sensu lato*, based on features of the pelvis and pes. The separation of *Dyoplosaurus acutosquameus* from *Euoplocephalus tutus* was supported by a phylogenetic analysis by Thompson et al. [32]; *Dyoplosaurus acutosquameus* was recovered as the sister taxon of *Pinacosaurus mephistocephalus* Godefroit et al., 1999 [33], and is well removed from *Euoplocephalus tutus*.

Penkalski and Blows [34] reassessed the holotype of *Scolosaurus cutleri* and found it to be distinct from *Euoplocephalus tutus* and *Dyoplosaurus acutosquameus* as well. These authors also noted that *Scolosaurus* differed from *Euoplocephalus* in the morphology of the cervical half rings, osteoderms, humerus, and radius, in the texture of the osteoderms, and in overall size. *Scolosaurus* differed from *Dyoplosaurus* in the morphology of the osteoderms, pelvis, and pedal unguals.

Vickaryous and Russell ([16]∶161), like Coombs [4], found that variable morphological features in specimens referred to *Euoplocephalus* did not co-occur exclusively in some specimens and not others; in other words, variable features occur randomly among *Euoplocephalus* specimens. This constitutes a testable hypothesis for the variation in *Euoplocephalus tutus*; if the same combination of variable features is present in some specimens but not others, then there may be justification for the segregation of *Euoplocephalus tutus* into multiple species. Furthermore, if these combinations of variable features are stratigraphically separated, this would provide additional support for the hypothesis that more than one species is currently included in *Euoplocephalus tutus*. Continued collecting in western Canada and the USA has produced additional ankylosaurid specimens, which may provide new information about variation in *Euoplocephalus tutus*. Now, there is also a better understanding of the stratigraphic distribution of dinosaur faunas in Alberta [18], [35], [36] as well as the stratigraphic placement of ankylosaur specimens with which to assess stratigraphic variation in *Euoplocephalus tutus*.

In this paper, the apparent stratigraphic longevity of *Euoplocephalus tutus* is investigated by conducting a detailed review of all specimens referred to *Euoplocephalus tutus*, as well as specimens that were previously referred to *Euoplocephalus* but that are now identified as *Dyoplosaurus* and *Scolosaurus*. Variation in *Euoplocephalus tutus* is assessed by looking for morphological groupings among specimens referred to *Euoplocephalus tutus*, and looking for stratigraphic patterns that correspond to any of these morphological groupings. The taxonomic statuses of the junior synonyms of *Euoplocephalus tutus* are then reassessed. Finally, the phylogenetic relationships of *Euoplocephalus tutus* are investigated with any resurrected or new species within the Ankylosauridae.

## Materials and Methods

### Ethics Statement

No permits were required for the described study, which complied with all relevant regulations.

### Material Examined

Evaluating morphological variation in *Euoplocephalus tutus* is confounded by the fragmentary nature of the holotype specimen, CMN 210, which consists of only the skull roof of the antorbital region, and a partial first cervical half ring. Cervical half ring morphology has been considered taxonomically useful [13], [24], [34]. Based on the forms of the first cervical half rings, two additional specimens have recently been referred to *Euoplocephalus tutus*: AMNH 5406 [13], and UALVP 31 [24]. AMNH 5406 consists of the shoulder girdle and forelimbs, and UALVP 31 includes a skull, right scapula, partial pelvis, both humeri, femur, tibia, metatarsal, and osteoderms. Together these specimens increase the amount of definitive *Euoplocephalus tutus* skeletal material, which can then be compared to other referred specimens. When Arbour et al. [24] was published, UALVP 31 was still undergoing preparation; this specimen is now fully prepared and described herein.

All of the specimens collected by Canada Fossils Ltd. were prepared as display specimens and have been heavily reconstructed; it is difficult to determine the extent of real bone in FPDM V-31, NSM PV 20381, and TMP 2001.42.19. In this paper, only elements of these specimens that are obviously original fossils are described. Photographs of these specimens prior to reconstruction were provided by A. Dzindic.

The holotype of *Dyoplosaurus acutosquameus* (ROM 784) was redescribed in detail by Arbour et al. [24]. However, since that paper was published the skull has been removed from display, and the ventral surface has been revealed for the first time. Discussion of ROM 784 in this paper is limited to comparisons with other specimens referred to *Euoplocephalus*.

### Terminology

**Figure 2:**
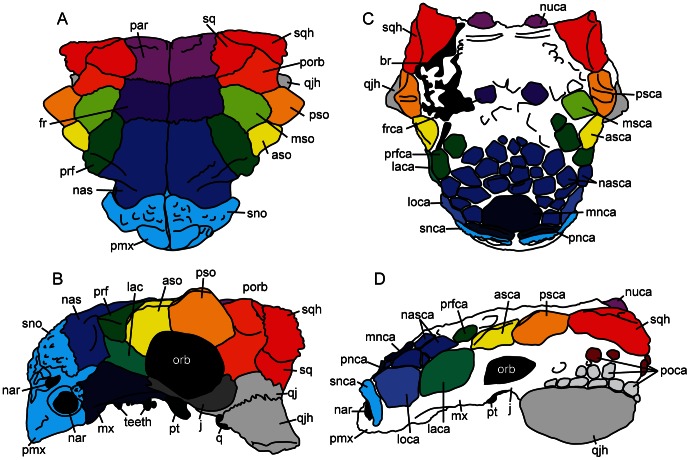
Cranial anatomy of ankylosaurids, including terminology for ornamentation patterns. ZPAL MgD II/1, juvenile *Pinacosaurus grangeri* in **A**) dorsal and **B**) left lateral views, showing boundaries of cranial bones. Boundaries between cranial bones are not visible in most adult ankylosaurids. **C**) UALVP 31, *Euoplocephalus tutus*, in dorsal view. **D**) CMN 8530, *Anodontosaurus lambei* (holotype), in left lateral view. Cranial ornamentation that is subdivided into discrete polygons (rather than generalized rugosity) are referred to as caputegulae. Abbreviations: asca, anterior supraorbital caputegulum; aso, anterior supraorbital; br, break or plaster; fr, frontal; frca, frontal caputegulum; j, jugal; lac, lacrimal; laca, lacrimal caputegulum; loca, loreal caputegulum; mnca, median nasal caputegulum; msca, middle supraorbital caputegulum; mso, middle supraorbital; mx, maxilla; nar, naris; nas, nasal; nasca, nasal caputegulum; nuca, nuchal caputegulum; orb, orbit; par, parietal; pmx, premaxilla; pnca, postnarial caputegulum; poca, postocular caputegulum; porb, postorbital; prf, prefrontal; prfca, prefrontal caputegulum; psca, posterior supraorbital caputegulum; pso, posterior supraorbital; pt, pterygoid; q, quadrate; qj, quadratojugal; qjh, quadratojugal horn; snca, supranarial caputegulum; sno, supranarial ornamentation; sq, squamosal; sqh, squamosal horn.

Cranial ornamentation is useful for identifying differences and similarities among ankylosaur taxa, but a brief review of relevant terminology is required before reviewing variation in specimens referred to *Euoplocephalus*. Ankylosaur cranial ornamentation may arise either through coossification of osteoderms to the underlying skull bones, through elaboration of the skull elements themselves, or through a combination of both processes [15], [37]. Many ankylosaurs have flat cranial ornamentation subdivided by shallow furrows (e.g. *Ankylosaurus*, *Edmontonia*), and in some ankylosaurs these discrete areas are bulbous (e.g., *Saichania chulsanensis* Maryanska, 1977 [27]). Blows [38] created the term caputegulum (Latin, “skull tile”; plural caputegulae) for the flat bones covering the skulls of ankylosaurs. This term is useful because it does not matter whether or not the discrete polygons of cranial ornamentation are formed by coossified osteoderms or cranial sculpturing (or both). The ability to identify and describe ornamentation patterns by naming discrete caputegulae facilitates the comparison of individual specimens and species. The term is here used with modifiers indicating the location (e.g. prefrontal caputegulum, supraorbital caputegulae), to compare cranial ornamentation patterns across ankylosaur taxa (Fig. 2). The pyramidal ornamentations of the squamosals and quadratojugals have been variously referred to as scutes [7], [22], bosses [16], horns [27], [39], and coronuces [38]. The term “horn” is used in this paper to refer either to the squamosal or quadratojugal ornamentation.

### Stratigraphic and Geographic Positions of Specimens

Evans [40] outlined methods for resolving the biostratigraphic distribution of lambeosaurine dinosaurs in Dinosaur Provincial Park, using high-precision differential GPS coordinates of known quarries and Oldman-Dinosaur Park Formation contacts that were published on a supplemental CD-ROM by Currie and Russell [35]. (This method in turn was derived from similar methods used by Ryan [41] to evaluate the stratigraphic position of centrosaurine dinosaurs in Dinosaur Provincial Park). In this way, the elevation above the Oldman-Dinosaur Park contact (and thus the stratigraphic position within the Dinosaur Park Formation) could be calculated for each specimen. Software updates to ArcGIS have unfortunately made the data on the CD-ROM unreadable, and so a modified version of the method proposed by Evans [40] using Google Earth is used here. Several *Euoplocephalus* specimens have also been collected from the Horseshoe Canyon Formation, but their quarries have not been relocated. Many *Euoplocephalus* quarries within Dinosaur Provincial Park have not been relocated, and so some specimen locality data are less precise.

Specimen locality data (Table S1, Locality Data S1) were collected from online collections databases (AMNH [42], TMP [43]), institutional catalogues (UALVP), specimen cards (AMNH, CMN, NHMUK, ROM, TMP, UALVP, USNM), field notes (CMN, also as the Geological Survey of Canada, GSC, or National Museum of Canada, NMC; ROM), from previously published coordinates in Currie and Russell ([35]: supplementary CD-ROM), from Steveville Map 969A [44], and from discussions with other researchers. Latitude and longitude coordinates (or UTM coordinates) were entered into Google Earth (Locality Data S1). Some specimen data are in the form of Township and Range coordinates, and these were converted to UTM coordinates using the Alberta Geological Survey’s online conversion tool [45]. Finally, the positions of specimens without township and range or GPS coordinates (largely those collected prior to 1980) were estimated from field notes and Google Earth measurement tools. For example, field notes by B. Brown and P. Kaisen for AMNH 5409 (available via the AMNH online Vertebrate Paleontology Archives [46]) indicate that this specimen was collected 20 feet above the left bank of the Red Deer River, 1.5 miles below the town of Steveville. Steveville was located in the northwest corner of Dinosaur Provincial Park, and the location 1.5 miles downstream can be estimated using the ruler tool in Google Earth. Then the appropriate elevation above river level can be determined. The position of AMNH 5409 has also been measured using differential GPS [35], and these coordinates correspond to estimates made based on Brown and Kaisen’s notes and Google Earth tools.

Elevation above the Oldman-Dinosaur Park formational contact was estimated for each specimen. Eberth [47] created a map showing the elevations of the contact throughout Dinosaur Park, and this was digitally overlaid in Google Earth. For each specimen, the plotted elevation was noted, as was the Oldman-Dinosaur Park contact elevation segment from Eberth [47]. Using Microsoft Excel, estimates for elevation above the contact were calculated for each specimen, and plotted to show the distribution of specimens in the Dinosaur Park Formation. For specimens that had both field note estimates and accurate GPS data, both elevations were plotted to demonstrate the potential range of error for specimens with only field note estimates.

### Phylogenetic Analyses

The phylogenetic relationships of *Euoplocephalus tutus,* as well as the resurrected ankylosaurid species *Anodontosaurus lambei*, *Dyoplosaurus acutosquameus*, and *Scolosaurus cutleri*, were investigated using T.N.T. v1.1 [48]. Three data matrices (Character Matrix S1–3) were prepared using the character matrix in Thompson et al. [32]:

1. the ‘original’ matrix in which all previous character codings were retained, except for moving data to *Anodontosaurus lambei and Scolosaurus cutleri* from *Euoplocephalus tutus*, in order to understand the effects of the addition of new taxa to the matrix;
2. an ‘updated codings’ matrix in which numerous character codings were revised (changes are explained in Character Statements S1), with many changes in particular to the codings for *Dyoplosaurus acutosquameus*, *Minotaurasaurus ramachandrani* Miles and Miles, 2009 [49] *Nodocephalosaurus kirtlandensis* Sullivan, 1999 [50], and *Tianzhenosaurus youngi* Pang and Cheng, 1998 [51], in order to correct incorrectly coded characters in the original matrix, and;
3. a ‘new characters’ matrix in which new characters identified in this paper were added to the ‘updated codings’ matrix.

The dataset was assembled in Mesquite version 2.72 [52], and a maximum of 177 characters (in analysis 3) and 18 taxa were used in the analysis. The analyses include 14 ingroup taxa consisting only of unequivocal ankylosaurine ankylosaurids, and the outgroup taxa *Lesothosaurus* (a basal ornithischian), *Scelidosaurus* (a basal thyreophoran), *Stegosaurus* (a stegosaur) and *Edmontonia* (a nodosaurid ankylosaur). Characters were treated as unordered and of equal weight. A parsimony analysis was conducted in T.N.T. using the Traditional Search option with one random seed and 1000 replicates of Wagner trees and the tree bisection reconnection (TBR) swapping algorithm. A strict consensus and a 50% majority rule consensus tree was created where more than one tree was recovered; for Analysis 3, a reduced consensus tree was also created using Mesquite. Because of the poor resolution of the strict consensus trees in Analysis 3, Matrix 3 was analyzed using the software program TAXEQ [53] to search for taxonomic equivalents that could be safely deleted and thereby reduce the amount of missing data in the analysis (“Safe Taxonomic Reduction” [54], [55]). The data were then subjected to a bootstrap analysis that was resampled with 1000 replicates to create a bootstrap tree using a heuristic search with the TBR swapping algorithm. Bremer supports were calculated in T.N.T., and the consistency and retention indices were calculated in Mesquite. Character state changes were investigated in Mesquite using the “Parsimony Ancestral States” analysis.

## Results

### Morphological Variation in Specimens Referred to *Euoplocephalus tutus*

The skull of *Euoplocephalus tutus* has been described and illustrated by several authors [4], [7], [11]–[17], and so only new observations of variable features are provided here. Descriptions of the postcrania of *Euoplocephalus tutus* by Coombs [6]–[10], Carpenter [3], Penkalski [13], and Arbour et al. [24] include information on most, but not all, aspects of the postcranial skeleton; in particular, the pre-caudal vertebral series has received relatively little attention. As such, more detailed descriptions and comparisons of the postcrania of specimens referred to *Euoplocephalus tutus* are presented. These descriptions include newly collected or newly prepared specimens in the TMP and UALVP collections, as well as a review of previously published specimens.

#### Cranium

**Figure 3:**
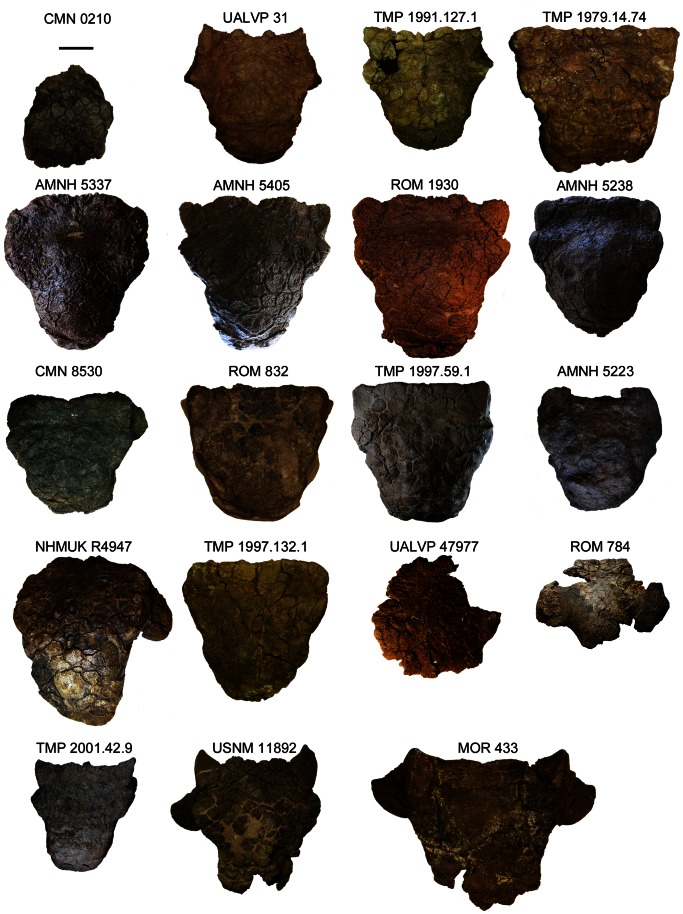
Skulls in dorsal view. CMN 0210 is the holotype of *Euoplocephalus tutus*, CMN 8530 is the holotype of *Anodontosaurus lambei*, MOR 433 is the holotype of *Oohkotokia horneri*, and ROM 784 is the holotype of *Dyoplosaurus acutosquameus*. AMNH 5337, AMNH 5405, CMN 0210, ROM 784, ROM 1930, TMP 1979.14.74, TMP 1991.127.1, TMP 1997.132.1, and UALVP 31 are from the Dinosaur Park Formation. AMNH 5238 and UALVP 47977 are of uncertain stratigraphic position within Dinosaur Provincial Park. AMNH 5223, CMN 8530, ROM 832, and TMP 1997.59.1 are from the Horseshoe Canyon Formation. NHMUK R4947 is from an unknown stratigraphic position in Alberta. MOR 433, TMP 2001.42.9 (much of the anterior rostrum in heavily reconstructed), and USNM 11892 are from the Upper Two Medicine Formation in Montana. Scale equals 10 cm. Photograph of ROM 832 by C. Brown, and of ROM 1930 by J. Arbour, and used with permission.

**Figure 4:**
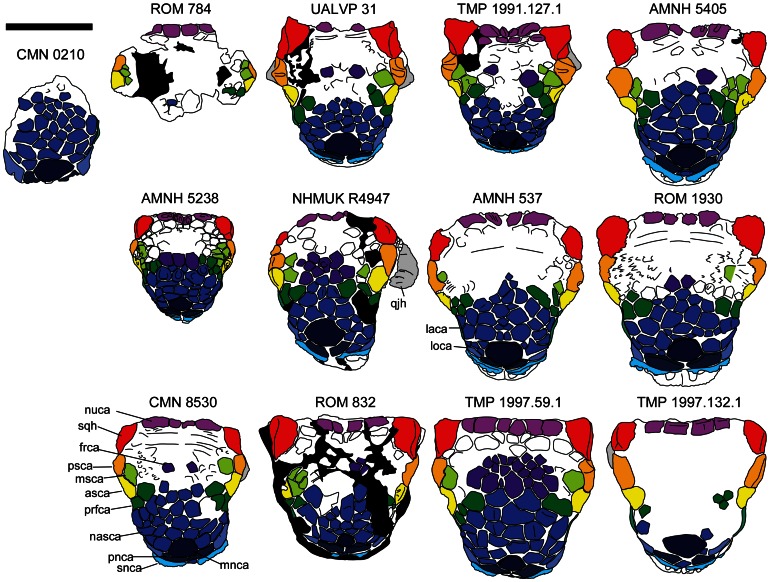
Cranial ornamentation patterns compared. CMN 0210 is the holotype of *Euoplocephalus tutus*, CMN 8530 is the holotype of *Anodontosaurus lambei*, and ROM 784 is the holotype of *Dyoplosaurus acutosquameus*. **Abbreviations:** asca, anterior supraorbital caputegulum; frca, frontal caputegulum; laca, lacrimal caputegulum; loca, loreal caputegulum; mnca, median nasal caputegulum; msca, middle supraorbital caputegulum; nas apt, nasal aperture; nasca, nasal caputegulum; nuca, nuchal caputegulum; orb, orbit; pnca, postnarial caputegulum; prfca, prefrontal caputegulum; psca, posterior supraorbital caputegulum; qjh, quadratojugal horn; snca, supranarial caputegulum; sqh, squamosal horn.

**Figure 5:**
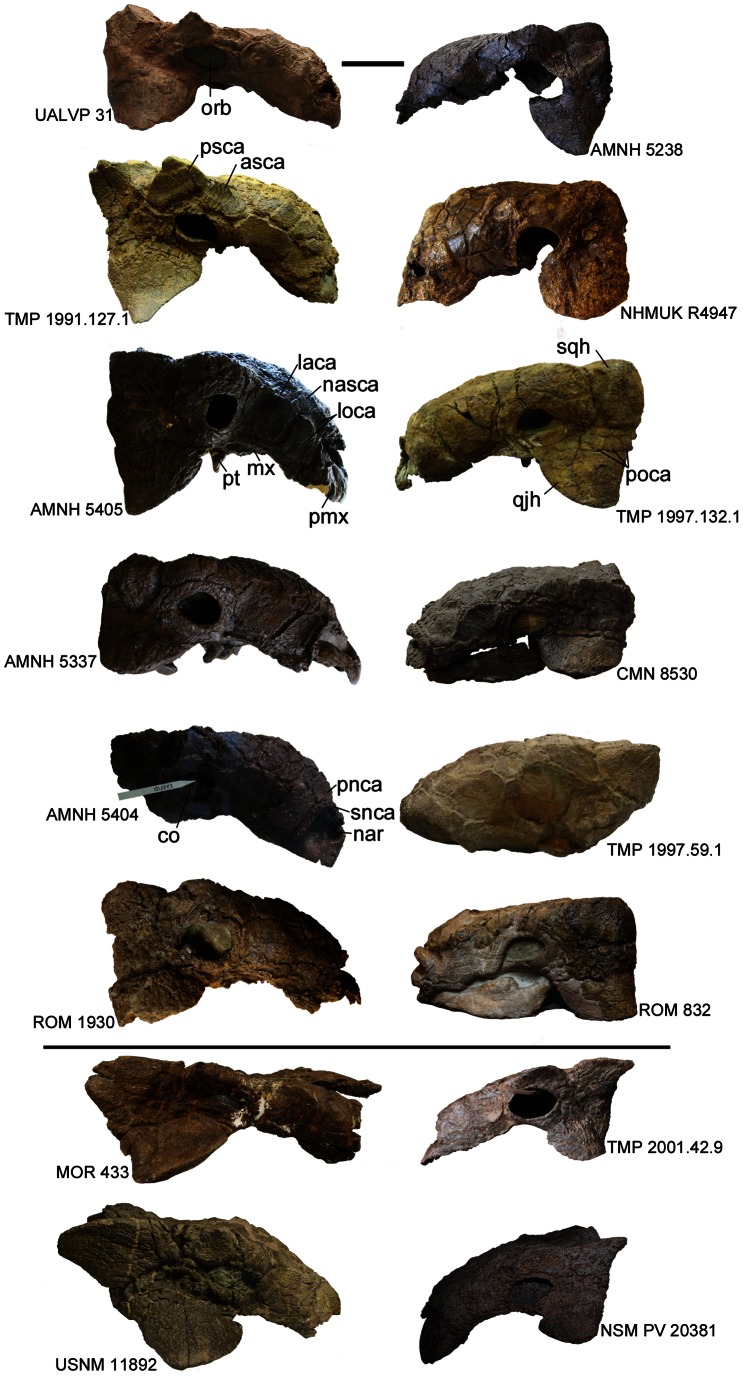
Skulls in lateral view. Skulls from Alberta appear above the horizontal line, and skulls from Montana below the line. The left column of skulls from Alberta includes skulls without postocular caputegulae around the base of the squamosal and quadratojugal horns, in right lateral view (AMNH 5337, AMNH 5404, AMNH 5405, ROM 1930, TMP 1991.127.1, and UALVP 31). The right column of skulls from Alberta includes skulls with postocular caputegulae around the base of the squamosal and quadratojugal horns, in left lateral view (AMNH 5238, CMN 8530 (*Anodontosaurus lambei* holotype), NHMUK R4947, ROM 832, TMP 1997.59.1, TMP 1997.132.1. Below the horizontal line are skulls from Montana (MOR 433 (*Oohkotokia horneri* holotype), NSM PV 20381, TMP 2001.42.9, and USNM 11892). The anterior rostrum of TMP 2001.42.9 and NSM PV 20381 are heavily reconstructed. AMNH 5404, AMNH 5405, and TMP 1991.127.1 are mirrored left lateral views, and AMNH 5238 is a mirrored right lateral view. Photograph of NSM PV 20381 by T. Miyashita and used with permission. Scale equals 10 cm. **Abbreviations:** asca, anterior supraorbital caputegulum; co, ciliary osteoderm; laca, lacrimal caputegulum; loca, loreal caputegulum; mx, maxilla; nar, naris; nasca, nasal caputegulum; orb, orbit; pmx, premaxilla; pnca, postnarial caputegulum; poca, postocular caputegulum; psca, posterior supraorbital caputegulum; pt, pterygoid; qjh, quadratojugal horn; snca, supranarial caputegulum; sqh, squamosal horn.

**Figure 6:**
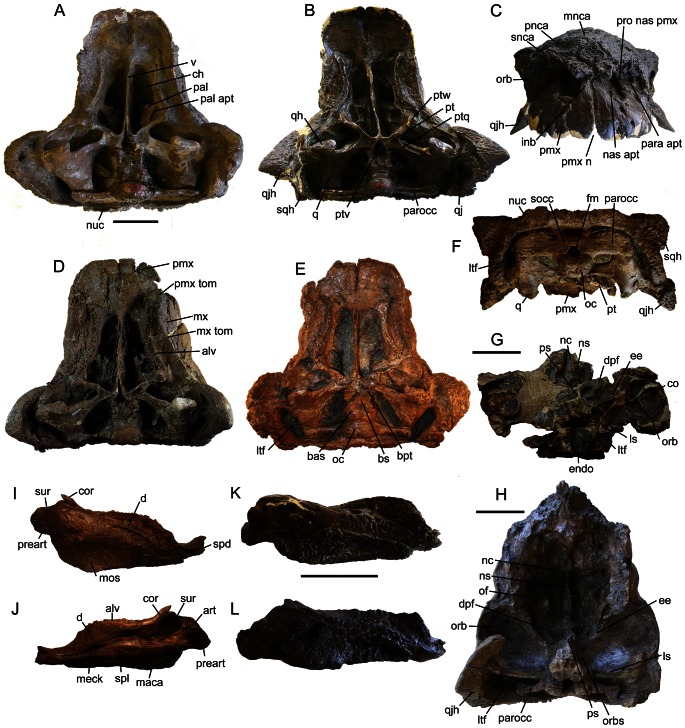
Cranial and mandibular anatomy. **A**) AMNH 5337 in ventral view. AMNH 5405 in **B**) ventral and **C**) anterior views. **D**) TMP 1997.132.1 in ventral view. ROM 1930 in **E**) ventral and **F**) posterior views. **G**) ROM 784 (holotype of *Dyoplosaurus acutosquameus*) in ventral view. **H**) AMNH 5238 skull in ventral view. Right mandible of UALVP 31 in **I**) lateral view and **J**) medial view. Right mandible in lateral view of **K**) AMNH 5405 and **L**) AMNH 5403. Scale bars equal 10 cm. **Abbreviations:** alv, tooth alveolus; art, articular; bas, basioccipital; bpt, basipterygoid process; bs, basisphenoid; ch, choana; co, ciliary osteoderm; cor, coronoid; d, dentary; dpf, descending process of frontal; ee, ectethmoid; endo, endocranial cavity; fm, foramen magnum; inb, internarial bar; ls, laterosphenoid; ltf, laterotemporal fenestra; maca, mandibular caputegulum; meck, Meckelian groove; mnca, median nasal caputegulum; mx, maxilla; mx tom, maxillary tomium; nas apt, nasal aperture; nc, nasal canal; ns, nasal septum; nuc, nuchal crest; oc, occipital condyle; of, olfactory region of nasal canal; orb, orbit; orbs, orbitosphenoid; pal, palatine; pal apt, palatal aperture; para apt, paranasal aperture; parocc, paroccipital process; pmx, premaxilla; pmx n, premaxillary notch; pmx tom, premaxillary tomium; pnca, postnarial caputegulum; preart, prearticular; pro nas pmx, intranasal process of premaxilla; ps, parasphenoid (cultriform process); pt, pterygoid body; ptq, quadrate ramus of pterygoid; ptv, interpterygoid vacuity; ptw, pterygoid wing; q, quadrate; qh, quadrate head; qjh, quadratojugal horn; snca, supranarial caputegulum; socc, supraoccipital; spd, sulcus for predentary; spl, splenial; sqh, squamosal horn; sur, surangular; v, vomer.

Skulls referred to *Euoplocephalus tutus* (Figs. 2, 3, 4, 5, 6, Figs. S1, S2, S3, S4, S5, S6, S7, S8, S9, S10, S11, S12, S13, S14, S15, S16, S17, S18, S19, Tables S2 and S3) have received a great deal of attention in the literature, but less attention has been paid to the shapes and patterns of the cranial caputegulae. Examination of 22 complete or partial skulls, and numerous cranial fragments (such as isolated quadratojugal horns or small skull fragments), shows that some caputegulae are consistent in form and location, and homologies can be proposed for these elements (Figs. 2, 3, 4, 5). These include the supranarial (sensu 16), postnarial, median nasal, loreal (anterior to the orbit, e.g. 56), prefrontal, supraorbital, and nuchal caputegulae, and the squamosal and quadratojugal horns. The arched supranarial caputegulae form the rim of the external nares, and are usually more rugose than the other caputegulae. The postnarial caputegulae are paired, subrectangular, flat caputegulae posterior to the supranarial caputegulae. Posterior to the postnarial caputegulae, and centered on the midline of the skull, is the large, hexagonal, median nasal caputegulum. A large, keeled caputegulum posterior to the postnarial caputegulae (loreal caputegulum) forms the lateral edge of the snout and extends onto the dorsal surface of the skull. A similar caputegulum is found posterior to the loreal caputegulum, on the lacrimal, but this does not extend as far onto the dorsum. There are two supraorbital caputegulae, an anterior one and a posterior one, each of which is triangular in dorsal view and has a keel approximately in line with the keel of the squamosal horn. The supraorbital caputegulae do not have distinct peaks, but instead the lateral keel of each forms a continuous edge with the adjacent supraorbital. The posterior supraorbitals of TMP 1991.127.1 (Fig. 3, Fig. S7) and UALVP 31 (Fig. 3, Fig. S2) each have a prominent transversely-oriented sulcus, which is not visible on any other specimens. In lateral view, the posterior supraorbitals of TMP 1991.127.1 (Fig. 5, Fig. S7) and UALVP 31 (Fig. 5, Fig. S7) are prominent and triangular. In each specimen, the posterior supraorbital is lower and more rounded.

The frontals and nasals are completely obscured by the frontonasal caputegulae (Figs. 2, 3, 4). Each skull referred to *Euoplocephalus* has a unique pattern of frontonasal caputegulae, which are generally subcircular, hexagonal, or subrectangular. The posterior extents of distinct caputegulae vary between individual specimens, but in most specimens individual caputegulae are not visible in the parietal regions posterior to the supraorbitals. The nuchal caputegulae can also vary in size and shape; usually, there are four square-to-rectangular caputegulae, and the median pair is smaller than the lateral pair (Figs. 3, 4).

The squamosal horn is one of the most variable features on the skull in specimens referred to *Euoplocephalus tutus*, but is generally triangular in dorsal and lateral views (Figs. 3, 4). The length and sharpness of the squamosal horn varies, as does the angle at which the squamosal horn projects from the skull. The longest, most pointed squamosal horns are found in FPDM V-31, MOR 433, NSM PV 20381, TMP 2001.42.19, and USNM 11892 (Fig. 5, Figs. S5, S6, S9). TMP 1991.127.1 (Fig. 5, Fig. S7) and UALVP 31 (Fig. 5, Fig. S2) have pointed squamosal horns that are relatively shorter, whereas the shortest, bluntest squamosal horns are found in AMNH 5337 and AMNH 5403 (Fig. 5, Figs. S10, S11). In dorsal view, the posterior edge of the squamosal horn is nearly continuous with the nuchal crest in some specimens (ROM 832, TMP 1997.59.1, TMP 1997.132.1; Figs. 3–4, Figs. S8, S12, S13). In other skulls (AMNH 5337, AMNH 5405, ROM 1930; Figs. 3–4, Fig. S10, S14, S15), the squamosal horn is distinct from the nuchal crest in dorsal view. The squamosal horns of FPDM V-31, MOR 433, NSM PV 20381, TMP 2001.42.19, and USNM 11892 (Fig. 5, Figs. S5, S6, S9) are back-swept, i.e., a line drawn from the center of the base of the squamosal horn through the apex of the horn in lateral view is more horizontal in these specimens compared to other referred *Euoplocephalus* specimens like ROM 1930 or UALVP 31 (Fig. 5, Figs. S2, S15). The squamosal horns extend well past the nuchal caputegulae in FPDM V-31, MOR 433, NSM PV 20381, TMP 2001.42.19, and USNM 11892 (Fig. 3, Figs. S5, S6, S9), a condition more similar to that observed in *Ankylosaurus* than in other specimens referred to *Euoplocephalus*.

The quadratojugal horn also varies considerably in terms of size, sharpness, and angle of projection from the skull. In dorsal and lateral views, the apex of the quadratojugal horn may be sharp (AMNH 5405, TMP 1991.127.1, UALVP 31; Figs. 3, 4, 5, Figs. S2, S7, S14) or round (CMN 8530, NHMUK R4947; Figs. 3, 4, 5, Figs. S4, S16). The apex may be centrally positioned, so that the quadratojugal horn is an equilateral triangle in dorsal or lateral view (AMNH 5405, TMP 1991.127.1, UALVP 31; Figs. 3, 4, 5, Figs. S2, S7, S14), or posteriorly offset, so that the horn is a right-angle triangle (ROM 832, TMP 1997.132.1, USNM 11892; Figs. 3, 4, 5, Figs. S5, S8, S12). The orientations of the squamosal and quadratojugal horns are likely controlled by the taphonomic deformation of the skulls [31]. Some specimens referred to *Euoplocephalus tutus* have small circular caputegulae at the bases of the squamosal and quadratojugal horns postocular caputegulae (CMN 8530, TMP 1997.132.1; Fig. 5), and other specimens lack these caputegulae (AMNH 5405, UALVP 31; Fig. 5).

The shape of the posterior edge of the nuchal crest in dorsal view varies among specimens referred to *Euoplocephalus* (Figs. 3, 4). In most specimens, shallow notches separate the medial pairs of nuchal caputegulae (AMNH 5238, AMNH 5405, TMP 1991.127.1; Figs. S7, S14, S17). In other specimens, the posterior edges of the nuchal crests are straight (ROM 832, TMP 1997.59.1, TMP 1997.132.1; Figs. S8, S12, S13), as they are in *Dyoplosaurus* (ROM 784; Fig. S3).

AMNH 5405 and TMP 1991.127.1 have arched skulls in lateral view (Fig. 5, Figs. S7, S14), but in other specimens (AMNH 5403, CMN 8530, MOR 433; Fig. 5, Figs. S4, S6, S11) the anterodorsal profiles of the skulls are nearly flat. It is likely, but not certain, that these differences are the results of taphonomic deformation [31]. Posterior to the orbits, the parietal region varies from flat to concave in lateral profile.

Cranial sutures are generally undetectable in adult ankylosaurids [37]. Although cranial sutures on the dorsum are obliterated by ornamentation in all referred *Euoplocephalus* skulls, sutures are occasionally visible on the ventral surfaces of some specimens. For example, the contacts between the premaxilla and maxilla, pterygoid and palatine, pterygoid and quadrate, and quadrate and quadratojugal are visible in AMNH 5405 (Fig. 6B). In the palatal region (Fig. 6A, B, D, E), a longitudinal furrow at the midline between the paired premaxillae may be present or absent. Some specimens have depressions lateral to the palatal apertures.

The skull of ROM 784 has been prepared recently to expose the ventral surface of the skull roof, which has never been described (Fig. 6G). Both the dorsal and ventral surfaces of the skull have been eroded, including the braincase. Ciliary osteoderms are preserved adjacent to the dorsal surface of the orbital cavity. Ciliary osteoderms are also preserved in AMNH 5238, AMNH 5337, AMNH 5403, AMNH 5404, and AMNH 5405 [12],[57].

#### Mandible

The mandible of *Euoplocephalus* is described in detail by Vickaryous and Russell 16. Much of the variation in mandibular morphology in specimens referred to *Euoplocephalus* (Fig. 6I–L) results from taphonomic distortion. The mandible of AMNH 5403 is much lower and flatter than those of AMNH 5337, AMNH 5405, and UALVP 31, but the cranium of AMNH 5403 has clearly been taphonomically crushed. The coronoid projects markedly from the dorsal border of the mandible in UALVP 31 (Fig. 6I, J), but not in AMNH 5337, AMNH 5403 (Fig. 6L), or AMNH 5405 (Fig. 6K). The significance of this difference is unclear, but does not appear to be taphonomically related, as the coronoid is not abraded in AMNH 5337, AMNH 5403, or AMNH 5405, and AMNH 5405 does not appear taphonomically distorted.

#### Vertebral Column

**Figure 7:**
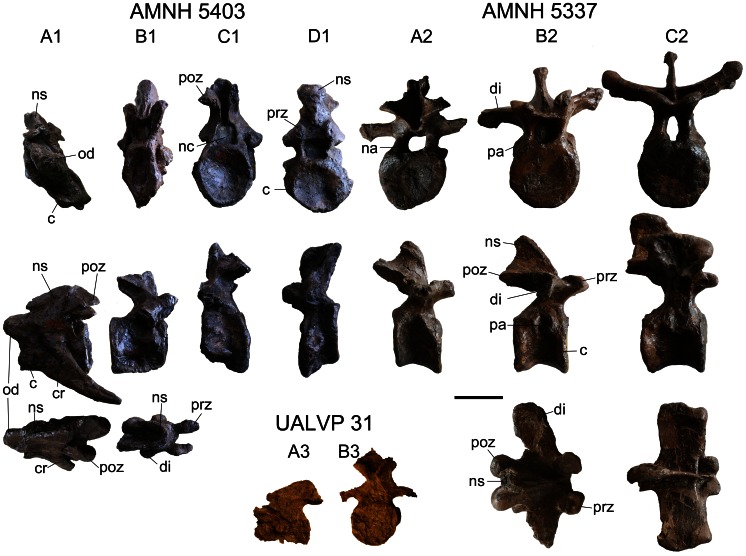
Cervical and dorsal vertebrae. AMNH 5403 axis (**A1**) in anterior, left lateral, and dorsal views; cervical (**B1**) in anterior, right lateral, and dorsal views; cervical (**C1**) in posterior, left lateral, views; cervical (**D1**) in anterior, left lateral views. AMNH 5337 cervical (**A2**) in posterior, right lateral views; dorsals (**B2**,**C2**) in anterior, right lateral, and dorsal views. UALVP 31 axis (**A3**) in left lateral view, and cervical (**B3**) in posterior view. **Abbreviations:** c, centrum; cr, cervical rib; di, diapophysis; ns, neural spine; na, neural arch; nc, neural canal; od, odontoid; pa, parapophysis; poz, postzygapophysis; prz, prezygapophysis.

Associated cervical vertebrae (Fig. 7, Table S4) are only preserved in AMNH 5337, AMNH 5403, and NHMUK R5161. The cervicals of AMNH 5403 are taphonomically distorted and asymmetrical. The cervicals are only partly visible in dorsal view in NHMUK R5161 as this specimen is displayed as a panel mount.

The atlas is unknown for *Euoplocephalus tutus*, but an axis is preserved in AMNH 5403 (Fig. 7). The axial centrum is anteroposteriorly longer than those of other cervical centra in AMNH 5403. The odontoid is wide and massive, with a shallow U-shaped trough on the dorsal surface. Cervical ribs are fused to the centrum; because of extensive plaster reconstruction it is unclear if the ribs are dichocephalic or holocephalic. In dorsal view, the neural spine is V-shaped, with the arms of the V directed posteriorly. The neural spine slopes dorsoposteriorly. Prezygapophyses are not preserved. The large postzygapophyses are located on the posterolateral ends of the V-shaped neural spine, and overhang the posterior end of the centrum. The articular faces of the postzygapophyses are oval and anteroposteriorly long.

One posterior cervical is preserved in AMNH 5337 (Fig. 7), and three postaxial cervicals are preserved in AMNH 5403 (Fig. 7). The cervical centra are wider than long or approximately as long as wide, with subcircular to elliptical amphicoelus articular faces. The position of the anterior face relative to the posterior face (dorsal or ventral to, or in line with) varies among the three vertebrae. The neural spine is transversely oriented and is U-shaped in dorsal view. In anterior view, the neural spine is an inverted triangle. Although partly damaged in all specimens, a thin horizontal sheet of bone, of unknown anterior extent, occurred between the widely-separated prezygapophyses. The prezygapophyses overhang the anterior edge of the centrum (unlike the postzygapophyses, which do not overhang the posterior edge of the centrum). There are no epipophyses. The neural canal is square in cross-section. The transverse process is low on the neural arch and projects ventrolaterally. The parapophysis is a subcircular protuberance positioned anteriorly on the centrum, although the dorsoventral position varies. Variation in the position of the articular faces relative to each other, the positions and sizes of the transverse processes (diapophyses), and the proportions of the centra in AMNH 5403 reflect positional differences along the vertebral column.

**Figure 8:**
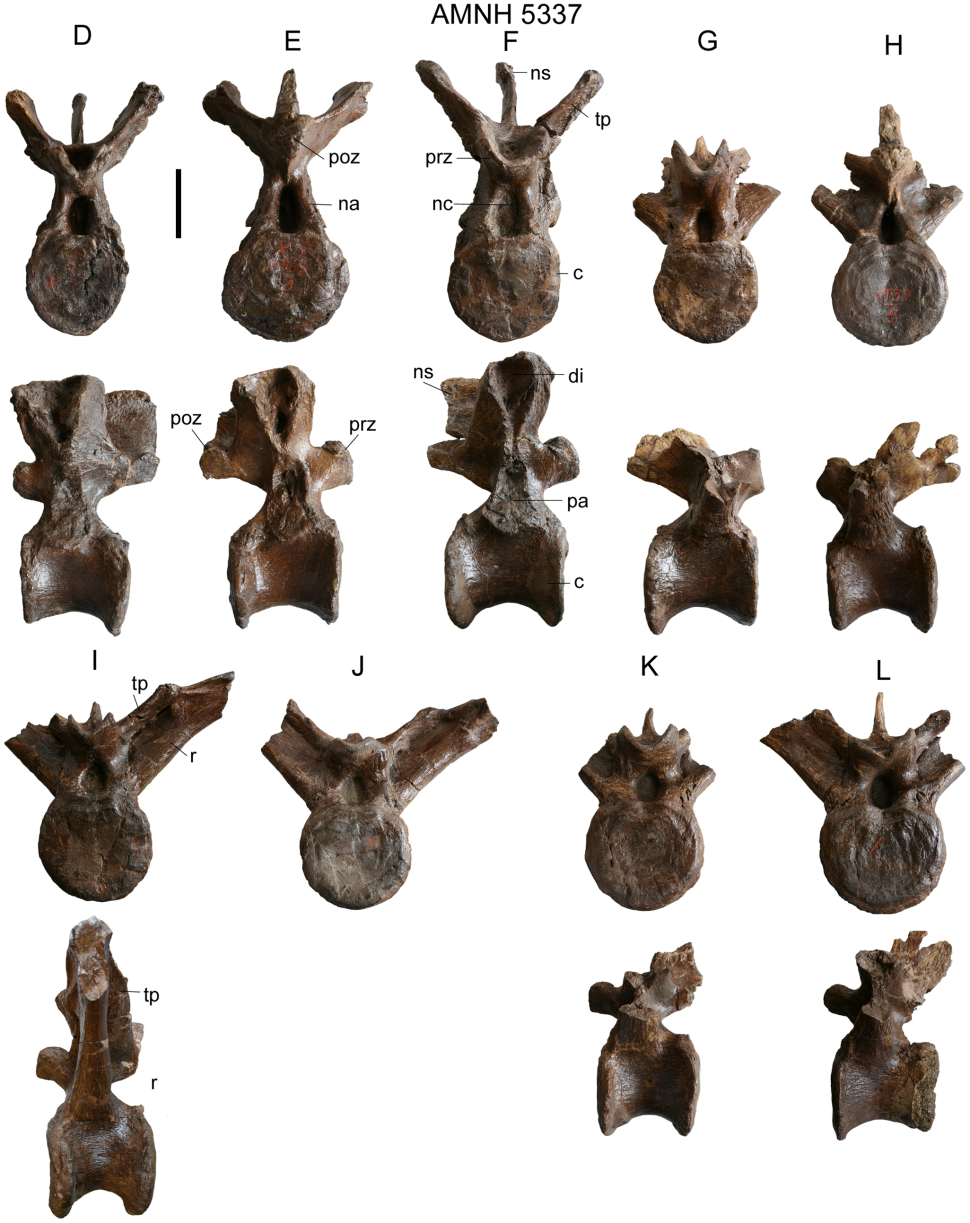
Dorsal vertebrae of AMNH 5337. AMNH 5337, **D** in anterior and left lateral views; **E** in posterior and right lateral views; **F** in anterior and right lateral views; **G** in anterior and right lateral views; **H** in posterior and left lateral views; **I** to **L** in anterior and left lateral views. **Abbreviations:** c, centrum; di, diapophysis; na, neural arch; nc, neural canal; ns, neural spine; pa, parapophysis; poz, postzygapophysis; prz, prezygapophysis; r, rib; tp, transverse process.

AMNH 5337 preserves the most complete presacral vertebral series of any specimen referred to *Euoplocephalus tutus*, and includes the final cervical vertebra (Fig. 7B1), eleven free dorsals (Figs. 7B2–3, 8), four dorsosacrals (dorsals incorporated into the sacral rod of the pelvis, with fused centra and neural spines), three sacrals, and one caudosacral. The parapophysis is located at the junction between the neural arch and centrum on one of the vertebrae (Fig. 7B2), and is transitional between the cervical and dorsal vertebrae; it is here considered as a dorsal vertebra because the morphology of the neural spine is more similar to those of the dorsals than cervicals. In addition to the location of the parapophysis, the dorsal vertebrae (Fig. 8, Table S4) can be differentiated from the cervicals based on morphological differences of the neural spines, which are anteroposteriorly-oriented and blade-like in the dorsals (rather than transversely-oriented and U-shaped, as in the cervicals). The shapes of the dorsal neural spines vary along the vertebral column; each is a mediolaterally thin and rectangular (in lateral view) plate that overhangs the posterior edge of the centrum. A rugose, mediolateral swelling occurs towards the distal end of the neural spine. The dorsal centrum is spool-shaped, with concave lateral sides and circular articular faces. The neural canal is tall and elliptical. The transverse processes are mediolaterally wide and anteroposteriorly long. The orientation at which they project from the neural arch varies from horizontal to dorsolateral. In some vertebrae, paired fossae occur at the junctions of the transverse processes, neural spines, and prezygapophyses (Fig. 7C2). The diapophysis is an inverted triangle on the end of the transverse process. The parapophysis is a subcircular to teardrop-shape articular surface in the anterior dorsals and sutural surface in the posterior dorsals. Posteriorly in the vertebral series, the dorsal ribs fuse to the dorsal vertebrae. The prezygapophyses are closely set and steeply angled, forming a U-shaped trough. The postzygapophyses are fused together along their lengths to form a peg-like, midline structure.

Features that vary among the dorsal vertebrae include the angles of projection of the transverse processes (more steeply inclined posteriorly in the series), the extents that the neural spines and postzygapophyses overhang the posterior ends of the centra (more overhanging posteriorly in the series), and whether or not the ribs are coossified to the transverse processes (unfused anteriorly, and fused posteriorly in the series). Because only one specimen preserves a relatively complete dorsal series (AMNH 5337), it is impossible to compare vertebrae in the same positions in different specimens; the dorsals of AMNH 5337 are similar in most respects, other than size, to those of *Ankylosaurus* [39]. Coombs [9], in describing the juvenile specimen AMNH 5266, noted that the dorsal centra were not as constricted midlength relative to the articular faces as in other *Euoplocephalus tutus* specimens, a difference he attributed to ontogenetic change. It is unknown at present how vertebral morphology may change throughout ontogeny in ankylosaurids. Additionally, Coombs [9] noted that the diapophyses of the preserved dorsal neural arch were less blade-like compared to *Ankylosaurus magniventris* and other *Euoplocephalus tutus* specimens.

**Figure 9:**
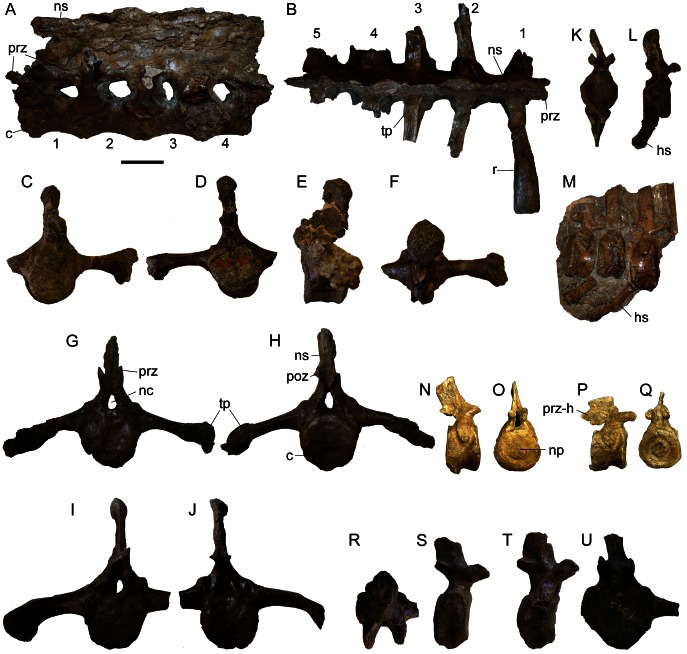
Dorsosacral, sacral, caudosacral, and caudal vertebrae. Partial sacrum of ROM 1930 in **A**) left lateral and **B**) dorsal (with anterior to the right) views. Sacrocaudal of AMNH 5245 in **C**) anterior, **D**) posterior, **E**) left lateral, and **F**) dorsal views. Sacrocaudal of ROM 1930 in **G**) anterior and **H**) posterior views. Sacrocaudal of ROM 1930 in **I**) anterior and **J**) posterior views; distal end of transverse process is partially reconstructed. Anterior free caudal vertebra of CMN 8530 (holotype of *Anodontosaurus lambei*) in **K**) anterior and **L**) right lateral views. **M**) Block of articulated anterior free caudal vertebrae of ROM 1930, in right lateral view. Penultimate free caudal vertebra of ROM 1930 in **N**) right lateral and **O**) anterior views. Transitional caudal vertebra (last free caudal vertebra before first handle vertebra of the tail club) of ROM 1930 in **P**) right lateral and **Q**) anterior views. AMNH 5404 free caudal vertebra in **R**) dorsal and **S**) right lateral views. Next most posterior AMNH 5404 free caudal vertebra in **T**) right lateral and **U**) anterior views. Scale equals 10 cm. **Abbreviations:** c, centrum; hs, haemal spine; nc, neural canal; np, notochordal prominence; ns, neural spine; poz, postzygapophysis; prz, prezygapophysis; prz-h, prezygapophysis of the first handle vertebra; r, rib; tp, transverse process.

**Figure 10:**
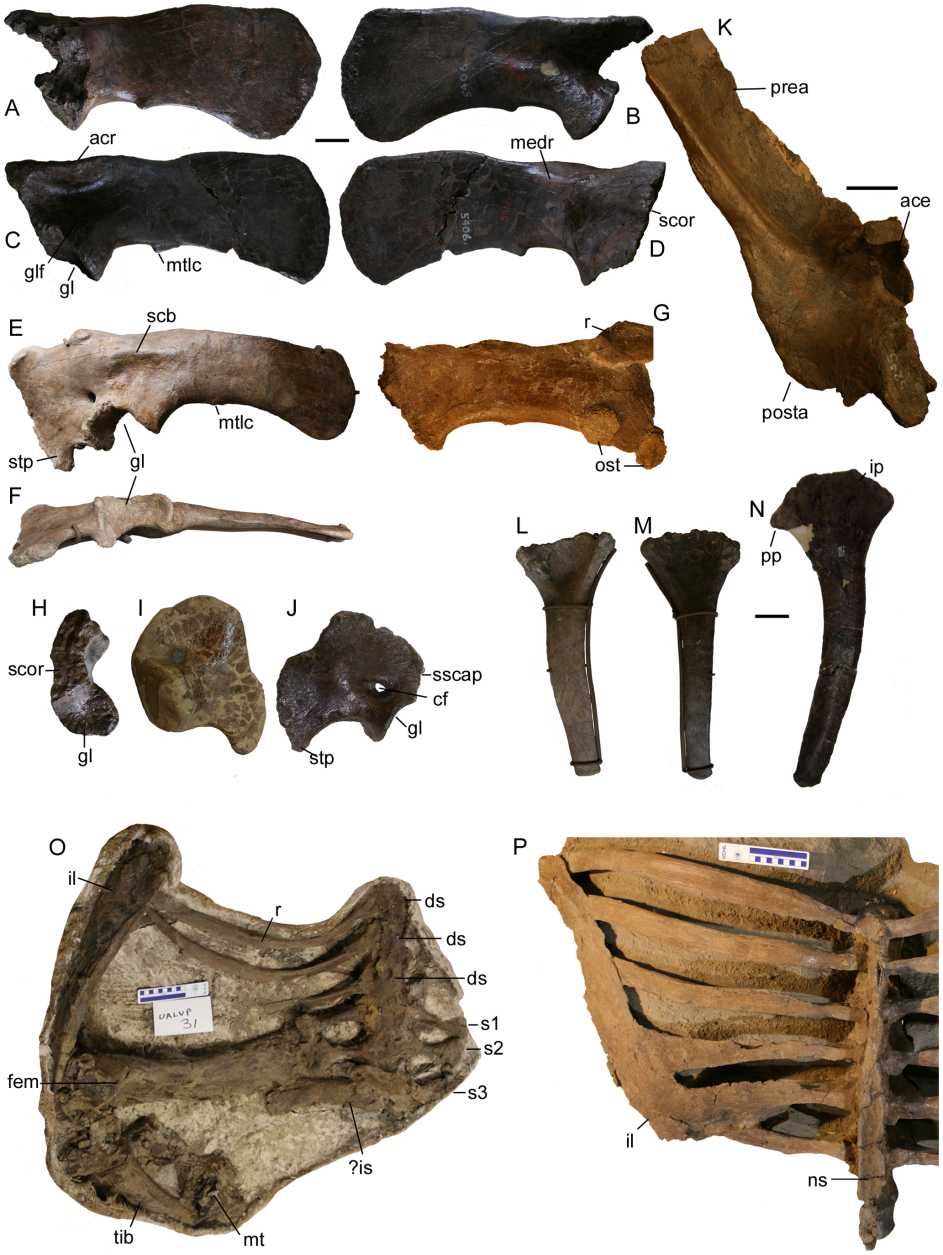
Pectoral and pelvic girdles. AMNH 5406 right scapula in **A**) medial and **B**) lateral views, left scapula in **C**) lateral and **D**) medial views. TMP 2001.42.19 left scapulocoracoid in **E**) lateral and **F**) ventral views. **G**) UALVP 31 right scapula in medial view. **H**) AMNH 5406 left scapula in anteroventral view. **I**) ROM 813 right coracoid in lateral view. **J**) AMNH 5404 left coracoid in lateral view. **K**) AMNH 5245 right ilium in ventral view, anterior is up. TMP 2001.42.19, **L**) right ischium and **M**) left ischium in medial views. **N**) CMN 8530 (*Anodontosaurus lambei* holotype) right ischium in medial view. **O**) UALVP 31 associated right ilium, sacrum, right femur and right tibia, with ilium in ventral view (anterior is up), and femur in medial view. **P**) TMP 1982.9.3 pelvis in dorsal view (the right half of the pelvis is reconstructed), anterior is up. Photograph of AMNH 5404 coracoid by R. Sissons and used with permission. Scale bar for A–H and J is 5 cm, scale bar for I is 10 cm. **Abbreviations:** ace, acetabulum; acr, acromion; cf, coracoid foramen; ds, dorsosacral; fem, femur; gl, glenoid; glf, glenoid fossa; il, ilium; ip, iliac peduncle; is, ischium; ost, osteoderm; medr, medial ridge; mt, metatarsal; mtlc, enthesis of M. triceps longus caudalis; ns, neural spine; posta, postacetabular process; pp, pubic peduncle; prea, preacetabular process; r, rib; s1–3, sacrals 1–3; scb, scapulocoracoid buttress; scor, surface for coracoid; stp, sternal process; sscap, surface for scapula; tib, tibia.

The synsacrum (Figs. 9, 10, Table S5) includes coossified dorsal, sacral, and caudal vertebrae. Currently, only the sacra of AMNH 5245, NHMUK R5161, and ROM 1930 can be observed in ventral view, as all of the other pelves are mounted for display with only the dorsal surface accessible. A full description of the pelvis of specimens referred to *Euoplocephalus tutus* is provided by Coombs [8]. Vickaryous et al. (2004) and Thompson et al. [32], only noted the presence or absence of the synsacrum, but did not fully describe it. Where sacral vertebrae are preserved, they are always coossified, except for AMNH 5266, a juvenile specimen [9]. The number of dorsosacrals and caudosacrals is variable. True sacrals are identified here as those that immediately bracket the acetabulum, and in all referred specimens there appear to have been no more than three. AMNH 5337 and AMNH 5409 each have four dorsosacrals, three sacrals, and one caudosacral. AMNH 5245 has two dorsosacrals, three sacrals, and one caudosacral, but the anterior end of the sacrum is broken and there were almost certainly additional dorsosacrals. The sacrum of ROM 1930 is in several pieces, but includes a block of five coossified vertebrae (with a sixth broken off), which appear to be dorsosacrals based on the flattened, T-shaped ribs (Fig. 9A, B). The most anterior vertebra in this section has free, unfused prezygapophyses, which indicates that this is the first vertebra in the fused sacral rod. The most posterior vertebra preserved in this section may be a sacral vertebra. A second section of fused vertebrae consists of two vertebrae that are most likely sacral vertebrae, based on the morphology of the centra and the large broken area representing the attachments of the sacral ribs. These two sections do not fit back together, so it is unclear if an additional vertebra is missing between them. In total, at least seven dorsosacral and sacral vertebrae formed the sacral rod of ROM 1930. There are an additional three unfused caudosacral vertebrae in ROM 1930 (Fig. 9 G–J). The distal ends of the transverse processes are large, not tapering, which suggests they contacted or fused with the ilia. This specimen also has three loose vertebrae, one of which is probably a true sacral, and two of which are probably caudosacrals. ROM 1930 may have had up to eleven vertebrae in the pelvis. NHMUK R5161 includes at least three dorsosacrals, three sacrals and three caudosacrals (see [21]:Pl. VI, Fig. 2). TMP 1982.9.3 preserves four dorsosacrals and two sacrals, with the posterior portion of the sacral rod broken (Fig. 10P).

The intervertebral facets of centra of all of the dorsosacral and sacral vertebrae are coossified in adult specimens (unfused sacral vertebrae are known in the juvenile specimen AMNH 5266), but the centra of the caudosacral vertebrae may not be coossified. The neural spines of all of the vertebrae of the sacrum fuse into a single continuous sheet of bone, such that the prezygapophyses and postzygapophyses become indistinct. In TMP 1982.9.3, the distal ends of the neural spines are laterally expanded, forming a flat to slightly concave dorsal surface (Fig. 10P). Although the neural spines are completely coossifed in ROM 1930, in dorsal view the individual neural spines form a repeating teardrop pattern (Fig. 9B). This region is unprepared in AMNH 5245, somewhat reconstructed with plaster in both AMNH 5337 and AMNH 5409, and obscured by skin impressions in NHMUK R5161. The centra of the dorsosacrals have lateral surfaces that are slightly more concave compared to the centra of the sacrals. Ventrally, the sacral vertebrae lack a midline groove (AMNH 5245) or have a shallow, discontinuous midline groove (ROM 1930). The transverse processes of the dorsosacrals are T-shaped in cross section, whereas those of the sacrals are more rectangular, and proportionately thicker in cross-section.

Arbour et al. [24] noted differences in the shapes of centra among caudal vertebrae referred to *Euoplocephalus tutus*, with most specimens having circular to subcircular cross-sections, and CMN 8530 being octagonal (Fig. 9K). Features of the caudal vertebrae (Fig. 9, Tables S5, S6) that do not appear to vary among specimens include the orientations of the neural and haemal spines, and the shapes of the neural and haemal spines (in all instances, the spines taper distally and are blade-like). The presence or absence of a notochordal prominence on the centrum varies among vertebrae within a single individual. The number of vertebrae incorporated into the tail club handle (terminology sensu [10]) may be a useful character, but few tail clubs are complete and this character cannot be coded in most specimens.

Penkalski [13] observed differences in the orientations of the articular faces of the zygapophyses in the caudal vertebrae, with ROM 784 (*Dyoplosaurus acutosquameus*) having more horizontal articular faces than those of AMNH 5404. However, the orientations of the articular faces vary along the caudal series in ROM 784, and the posterior caudals have more vertically oriented zygapophyseal articular faces.

#### Pectoral Girdle And Forelimb

All scapulae referred to *Euoplocephalus tutus* are dorsoventrally broad and paddle-shaped (Fig. 10A–G, Table S7). The posterior (distal) end of the scapular blade is weakly expanded, and the posteroventral edge of the blade is weakly concave. The distal end of the scapula is broader and rounder in AMNH 5406 (Fig. 10A–D) when compared to AMNH 5424 ([6]: Fig. 3). The acromion occurs on the dorsal border of each scapula, laterally overhangs the main body of the scapula, and is most prominent over the glenoid. The infraspinous fossa is approximately triangular and ventral to the acromion. The acromion gradually decreases in size along the posterior edge of the infraspinous fossa. A prominent enthesis probably marks the insertion of the M. triceps longus caudalis (as in *Ankylosaurus*, see [39]: Fig. 15) in AMNH 5406 (Fig. 10A–D), TMP 2001.42.19 (Fig. 10E), and UALVP 31 (Fig. 10G). On the medial side, a prominent horizontal ridge, the scapulocoracoid buttress, occurs at the junction of the scapula and coracoid. The scapula and coracoid are unfused in AMNH 5406 (Fig. 10H) but fused in the larger specimens AMNH 5337 and AMNH 5424 ([6]: Fig. 3) and in the smaller specimen TMP 2001.42.9 (Fig. 10E,F); the sutural edge of the scapula in ROM 1930 is broken, possibly indicating that it was fused to the coracoid. The right coracoid of ROM 813 (Fig. 10I) has been heavily reconstructed with plaster so that it is unclear if fusion with the scapula had occurred. In lateral view, the scapula has a triangular ventral projection at the glenoid. The scapula and the coracoid contribute about equally to the glenoid, and the coracoid sutural surface in AMNH 5406 is flat (Fig. 10H). The coracoid is approximately square in lateral view, with a straight anterior margin and a prominent, hooked ventral (sternal) process. The coracoid foramen is circular.

**Figure 11:**
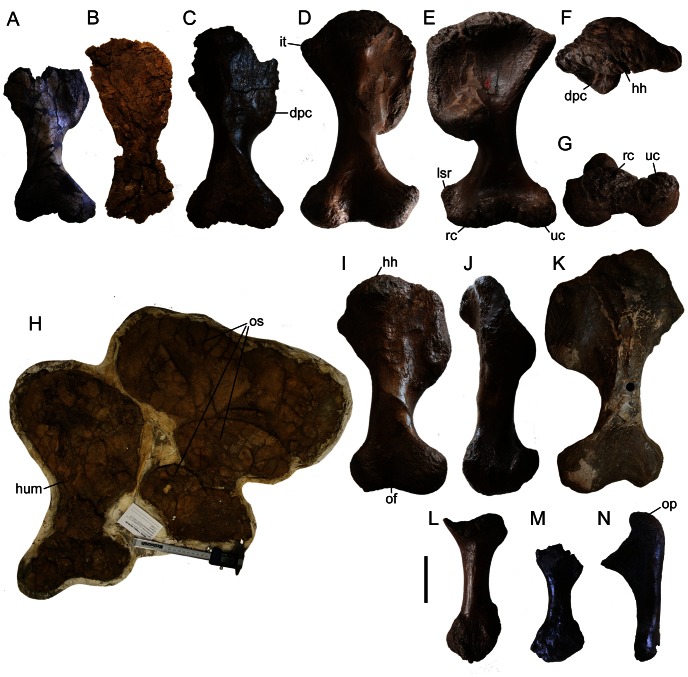
Forelimb elements. AMNH 5406 right humerus in **A**) posterior view. UALVP 31 right humerus in **B**) anterior view. ROM 1930 right humerus in **C**) posterior view. AMNH 5337 right humerus in **D**) posterior, **E**) anterior, and **F**) proximal and **G**) distal views. **H**) TMP 1997.132.1 left humerus and associated osteoderms with humerus in anterior view and osteoderms mostly in interior view. AMNH 5404 right humerus in **I**) posterior and **J**) anterior views. **K**) ROM 47655 left humerus in posterior view. AMNH 5337 right radius in **L**) medial view. AMNH 5406 **M**) right radius in medial view and **N**) right ulna in medial view. Scale bar equals 10 cm. **Abbreviations:** dpc, deltopectoral crest; hh, humeral head; hum, humerus; it, internal tuberosity; lsr, lateral supracondylar ridge; of, olecranon fossa; op, olecranon process; os, osteoderm; rc, radial condyle; uc, ulnar condyle.

All humeri referred to *Euoplocephalus tutus* (Fig. 11, Table S7) are stout and hourglass-shaped. The deltopectoral crest extends for more than 42% the length of the humerus (Table S7). Penkalski [13] noted that the deltopectoral crest in MOR 433 does not appear to extend as far down the shaft of the humerus compared to other *Euoplocephalus* specimens. This is difficult to quantify because the proximal and distal ends of both humeri are badly damaged in MOR 433, making the total length of each humerus impossible to determine. In all specimens where this feature is preserved, distally the lateral margin of the deltopectoral crest is rotated slightly anteriorly, and merges with the shaft of the humerus as a prominent, thick knob (e.g. AMNH 5337, Fig. 11E). Prominent striations on the deltopectoral crest represent the attachments for the M. supracoracoideus and M. pectoralis [6]. Humeri referred to *Euoplocephalus tutus* differ in the relative sizes of the deltopectoral crests (both in terms of length and width) and the lateral supracondylar crests. These crests are largest in AMNH 5337 (Fig. 11D, E) and smallest in AMNH 5406 (Fig. 11A) and UALVP 31 (Fig. 11B). The humerus of AMNH 5337 is longer than the humeri of AMNH 5406 or UALVP 31, and so the larger crests of AMNH 5337 may be size-related. The humeral head in proximal view is semicircular (Fig. 11F), and subcircular to slightly triangular in medial view. Anteriorly the broad, shallow, bicipital fossa is bounded by the deltopectoral crest and humeral head. The medial (internal) tuberosity is prominent, and the proximal margin posterior to the humeral head is flat. The radial (lateral) condyle is slightly larger than the ulnar (medial) condyle, although both are large and transversely expanded. The olecranon fossa is shallow and triangular, and the intercondylar notch is shallow and rounded. The radial and ulnar condyles and the humeral heads in the humeri of AMNH 5337, AMNH 5404, and ROM 1930 have networks of deep furrows covering the articular surfaces similar to those of large individuals of hadrosaurids, iguanodontids, ceratopsids, sauropods, and some theropods (Fig. 11F, G).

The radius is only known from a few specimens (Fig. 11L, M; Table S7). It is a stout bone with a flared, concave proximal articular surface, and a rugose, bluntly pointed distal end in anterior view (Fig. 11L, M). The proximal and distal ends of the radius of AMNH 5337 (Fig. 11L) are proportionately wider transversely than those of AMNH 5406 (Fig, 11M), ROM 784, and TMP 1997.132.1. In specimens where the ulnae are preserved, the proximal end has a prominent, rugose olecranon process (Fig. 11N, Table S7). A complete manus is not preserved in any specimen referred to *Euoplocephalus tutus*.

#### Pelvic Girdle And Hindlimb

**Figure 12:**
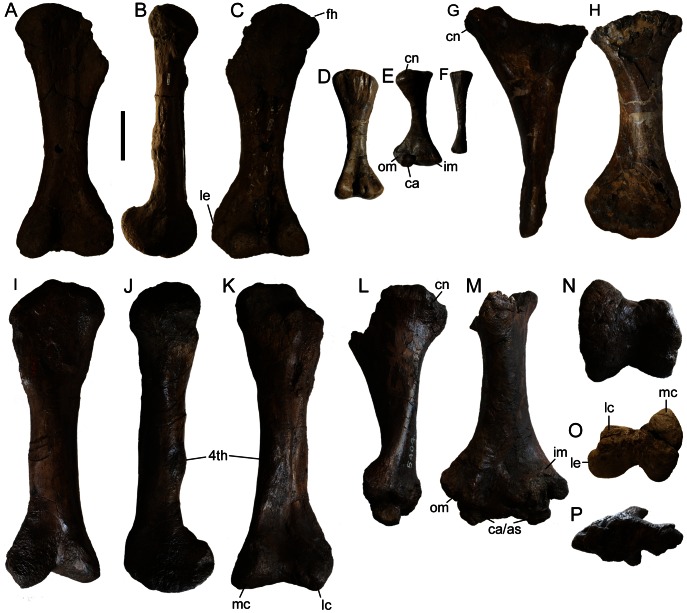
Hindlimb elements. TMP 1982.9.3 left femur in **A**) anterior, **B**) medial, and **C**) posterior views. **D**) AMNH 5266 right femur in posterior view. **E**) AMNH 5266 right tibia in anterior view. **F**) AMNH 5266 right fibula. ROM 813 left tibia in **G**) lateral and **H**) posterior views. AMNH 5404 right femur in **I**) anterior, **J**) medial, and **K**) posterior views, and right tibia in **L**) lateral and **M**) anterior views. **N**) AMNH 5404 right femur in distal view. **O**) TMP 1982.9.1 left femur in distal view. **P**) AMNH 5404 right tibia in distal view. **Abbreviations:** 4^th^, fourth trochanter; as, astragalus; ca, calcaneum; cn, cnemial crest; fh, femoral head; im, inner malleolus; lc, lateral condyle; le, lateral epicondyle; mc, medial condyle; om, outer malleolus.

**Figure 13:**
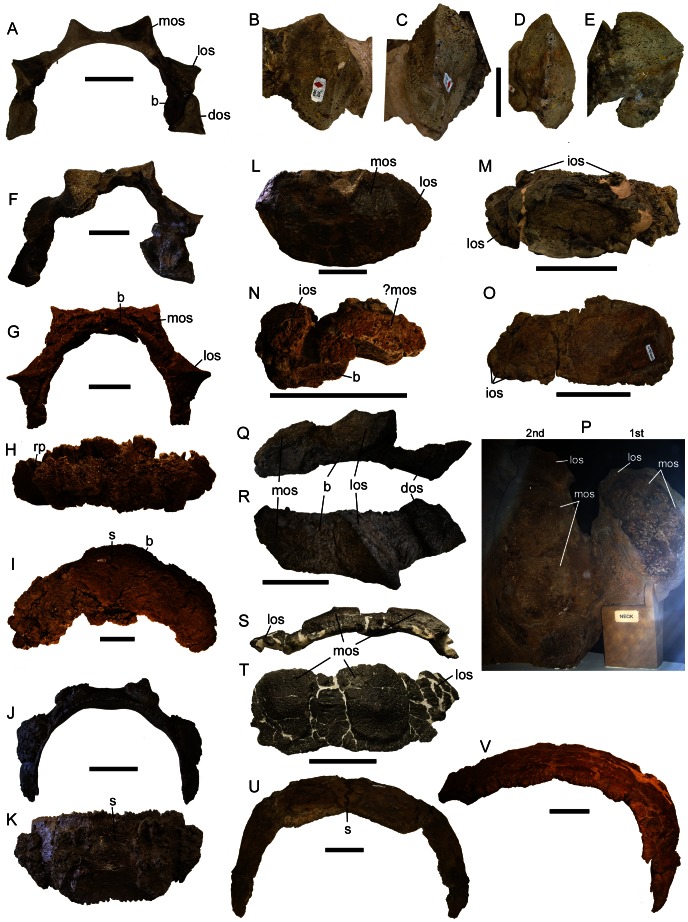
Cervical half rings. CMN 0210 (*Euoplocephalus tutus* holotype) first cervical half ring in **A**) anterior view; **B**) left medial osteoderm in superficial view; **C**) right lateral osteoderm in superficial view; **D**) right distal osteoderm in superficial view and **E**) dorsal view**. F**) First cervical half rings of AMNH 5406 in anterior view. UALVP 31, first cervical half ring in **G**) anterior and **H**) dorsal views, and second cervical half ring in **I**) dorsal view. AMNH 5337 first cervical half ring in **J**) anterior and **K**) dorsal views. **L**) AMNH 5404 first cervical half ring in dorsal view. **M**) CMN 8530 (holotype of *Anodontosaurus lambei*) first cervical half ring in dorsal view. **N**) Fragment of first cervical half ring of TMP 1982.9.3 in superficial view. **O**) Partial first cervical half ring of TMP 1997.132.1 in dorsolateral view. **P**) NHMUK R5161 in situ cervical rings in dorsal view, anterior is to the right. TMP 2001.49.2 partial first cervical half ring in **Q**) posterior and **R**) left lateral views. USNM 7943 partial first cervical half ring in **S**) anterior and **T**) dorsal views. **U**) TMP 2007.12.52 second cervical half ring in anterior view. **V**) UALVP 45931 partial second cervical half ring in anterior view. Scales in A, G–R equal 10 cm, scales in B–F equal 5 cm. **Abbreviations:** b, band; dos, distal osteoderm; ios, interstitial osteoderm; los, lateral osteoderm; mos, medial osteoderm; rp, resorption pit on medial osteoderm; s, suture between band segments.

The pelves of specimens referred to *Euoplocephalus* are mediolaterally broad, anteroposteriorly long, and have strongly divergent ilia (Fig. 10K, O, P; Table S8). Complete pelves are preserved in AMNH 5337, AMNH 5409 ([8]:Figs. 12, 13), and NHMUK R5161 ([21]:Pl VI, Fig.2, PL. VII, Fig. 1), and partial pelves are also known for AMNH 5245 (Fig. 10K), TMP 1982.9.3 (Fig. 10P) and UALVP 31 (Fig. 10O), as well as ROM 784 (*Dyoplosaurus,* [24]:Fig. 1). The postacetabular process of the ilium is proportionately longer in NHMUK R5161 compared to other referred specimens, and the process is longer than the maximum diameter of the acetabulum. The pubis is unknown. The ischium is wide proximally, and a sulcus on the lateral side contributes to the closed acetabulum (Fig. 10L, M). In medial view, the dorsal margin is rounded, and the iliac and pubic peduncles are not distinct from each other (Fig. 10N). The wide proximal end tapers abruptly into the ischial shaft. The ischial shaft is laterally compressed, and slightly sigmoidal in anterior and posterior views. The anterior and posterior margins are parallel for the length of the shaft, and the distal terminus is squared-off.

The femur (Figs. 12A–D, I–K, N, O) is stout and has a straight shaft with an oval cross-section. The femoral head is round, and the greater trochanter is neither prominent nor distinctly separated from the head. The fourth trochanter is a low and indistinct rugosity distal to the midlength of the femur. The distal condyles are posteriorly expanded, and the medial condyle is slightly larger than the lateral condyle. Posteriorly, the intercondylar groove is shallow. The lateral epicondyles are proportionately larger in AMNH 5266 (Fig. 12D) and TMP 1982.9.3 (Fig. 12C) than in AMNH 5404 (Fig. 12K).

The proximal and distal ends of the tibia (Figs. 12E–H, L, M, P) are greatly expanded relative to the shaft. In anterior view (Fig. 12H), the maximum dimension of the proximal end is slightly less than that of the distal end, whereas in lateral view (Fig. 12G), the proximal end is more than twice as wide as the distal end. In AMNH 5404 the astragalus is fused to the distal end of the tibia (Fig. 12L, M), but it is unfused in AMNH 5266 (Fig. 12E). Complete pedes are present in AMNH 5266 ([9]: Fig. 4) and ROM 1930; in each the pes is tridactyl, with U-shaped unguals (rather than triangular, as in ROM 784, *Dyoplosaurus acutosquameus*; [24]: Fig. 5) in dorsal view.

#### Osteoderms And Integument

The cervical half rings of ankylosaurids (Fig. 13, Table S9) are composed of two separate layers of ossification: a superficial (upper) layer of primary osteoderms similar to those found on the rest of the body (sometimes ringed by smaller interstitial osteoderms), and a deep (lower) layer of bone of unknown origin, referred to here as the band. The band is formed of several dorsoventrally arched, approximately rectangular segments joined by serrated sutures; most cervical rings have six segments. Each band segment may have zero (Fig. 13V), one (Fig. 13B), or more than one (Fig. 13N) osteoderm superficial to the band; most commonly a single large osteoderm is present and centered on the segment. In some specimens (AMNH 5337, AMNH 5404; Fig. 13J–L), the overlying osteoderm is fused to the underlying band, but in others (UALVP 31; Fig. 13G, H) the osteoderm is only partially fused or not fused at all to the band. Band segments are always smooth-textured and are more similar in appearance to endochondral bone than to osteodermal bone, which is typically pitted or rugose in ankylosaurids. Weathered band segments can have a fibrous, interwoven texture. In most ankylosaurids (*Ankylosaurus magniventris, Pinacosaurus mephistocephalus, and Saichania chulsanensis*), the morphologies of the first and second cervical half rings are similar, with the second half ring being larger and broader than the first.

Paired osteoderms on the cervical half ring share unique shapes, but the medial, lateral, and distal pairs differ from each other. In AMNH 5406, CMN 210 and UALVP 31 (Fig. 13A–H), the primary medial osteoderms have wide oval bases with anteroposteriorly-aligned keels, and the primary lateral osteoderms have narrower bases with sigmoidal keels [24]. The distal osteoderms (sensu [13]) are missing in UALVP 31 (Fig. 13G), but in CMN 210 (Fig. 13A) they are deeply excavated and compressed [24]. AMNH 5406, CMN 210, and UALVP 31 have the smallest known half rings referable to *Euoplocephalus tutus*. The half rings in AMNH 5337, AMNH 5403, AMNH 5404, and AMNH 5405 all have lower, more rounded and rugose osteoderms on the first half ring (Fig. 13J–L). The distal osteoderms are missing in all of these specimens, but because the distal osteoderms do not seem to be as strongly fused in AMNH 5406 and CMN 210, they may not have been preserved.

Several first cervical half rings referred to *Euoplocephalus tutus*, including CMN 8530, TMP 1982.9.3, TMP 1996.75.1, and TMP 1997.132.1, have small subcircular osteoderms present around the bases of the larger half ring osteoderms (Fig. 13M–O). These interstitial osteoderms are present even on small fragments of half rings (TMP 1982.9.3, TMP 1996.75.1; Fig. 13N). In CMN 8530 (Fig. 13M), only three of the interstitial osteoderms are preserved, but much of the dorsal surface of the half ring is broken. In TMP 1997.132.1 (Fig. 13O), the interstitial osteoderms ring the border of the preserved medial osteoderm, and are smaller and more irregularly distributed around the preserved lateral osteoderm. Unusually, AMNH 5404 has two knob-like projections on the ventral surface of the first cervical half ring, but these do not appear to be the same structures as the interstitial osteoderms found on other half rings.

The cervical rings in NHMUK R5161 (Fig. 13P) may have only four band segments rather than the six found in most other cervical rings referred to *Euoplocephalus tutus*. However, it is difficult to determine if the terminal edges of the half rings are broken or complete. No medial osteoderms are visible on the first cervical half ring, and if they are present, they are low and indistinct from the deep band. The lateral osteoderms have tall, laterally-directed keels and narrow bases, and are shaped like right-angle triangles in dorsal view. The second cervical ring also appears to have only four segments. The medial osteoderms are circular with posteriorly-directed apices. The lateral osteoderms are similar to those of the first cervical ring, but are somewhat more rectangular in dorsal view.

A partial first cervical half ring was found with TMP 2001.42.19 (Fig. 13Q, R), and preserves the right medial, lateral, and distal osteoderms. (Osteoderms associated with TMP 2001.42.19 have been mounted onto a curved armature for display over the skeleton, which also includes two fragments of either the first and/or second cervical ring.) The medial osteoderm is nearly flat. Although the keel on the lateral osteoderm is broken, it appears to have been tall and straight rather than sigmoidal, and the distal tip of the osteoderm overhangs the underlying band. The distal osteoderm has a tall keel, and envelopes the distal end of the band. The flat medial osteoderm is unlike the keeled, subconical medial osteoderms of AMNH 5406, UALVP 31, and many other referred *Euoplocephalus* specimens, but similar to that of NHMUK R5161. The apices of the osteoderm keels are usually more centrally positioned in specimens referred to *Euoplocephalus* (AMNH 5406, UALVP 31), and never overhang the band. An isolated first cervical half ring, USNM 7943 (Fig. 13S, T) also preserves nearly flat medial osteoderms with low, centrally positioned prominences.

Osteoderms along the body may also provide useful information, although few specimens preserve osteoderms in the original arrangements. Specimens that do retain *in situ* osteoderms include NHMUK R5161, ROM 813, ROM 1930, and TMP 1997.132.01. The *in situ* osteoderms of NHMUK R5161 were described in detail by Nopcsa [21] and Penkalski and Blows [34]. NHMUK R5161 has large, circular-based osteoderms covering most of the dorsal surface of the body, as well as paired, taller, conical osteoderms at the midline in the pectoral region.

ROM 813 is an exceptional specimen preserving abundant osteoderms, ossicles (<5 mm), and epidermal (soft-tissue) scale impressions [58]. Although it was referred to *Euoplocephalus tutus* by Penkalski [13], it preserves few diagnostic features of the Ankylosauridae, and none for the genus *Euoplocephalus tutus*. The straight shaft of the broken ischium, and the rugose, thin-walled osteoderms, suggest that ROM 813 is an ankylosaurid rather than a nodosaurid. The skeleton is disarticulated, but large portions of the integument remain intact. There are nine large blocks with *in situ* osteoderms. Two adjoining blocks contain a cluster of seven closely-packed large (length >25 cm) keeled osteoderms with rectangular bases. Each of these is surrounded by ossicles, and at the anterior edge of the cluster is a distinct crease similar to that found in NHMUK R5161. Another cluster of osteoderms surrounded by epidermal impressions and ossicles includes mostly osteoderms with subcircular bases, similar to those on the tail of NHMUK R5161. Unfortunately, it is difficult to determine the original positions on the body of many of the integument pieces, because the endochondral elements are disarticulated.

ROM 1930 includes abundant osteoderms that have been completely prepared from the surrounding matrix, as well as *in situ* osteoderms on a block containing several caudal vertebrae. Three large (width >15 cm) keeled osteoderms with oval bases are preserved, as well as hundreds of small (<5 mm) irregularly-shaped ossicles.

Two additional specimens (TMP 1997.132.01 and UALVP 31) include some osteoderms that may be close to their *in situ* positions. TMP 1997.132.01 preserves large (>20 cm diameter) osteoderms near the humerus, articulated radius and ulna, and tibia, as well as a second cervical half ring band with *in situ* (but not coossified) osteoderms. Osteoderms near the humerus are large, keeled, and have subcircular bases (Fig. 11H). Osteoderms near the radius and ulna are smaller, with peaked keels overhanging one end of the base, and with narrower bases compared to osteoderms near the humerus. The cervical ring osteoderms also have oval bases and low keels, and the peaks of the keels do not overhang the bases of the osteoderms.

**Figure 14:**
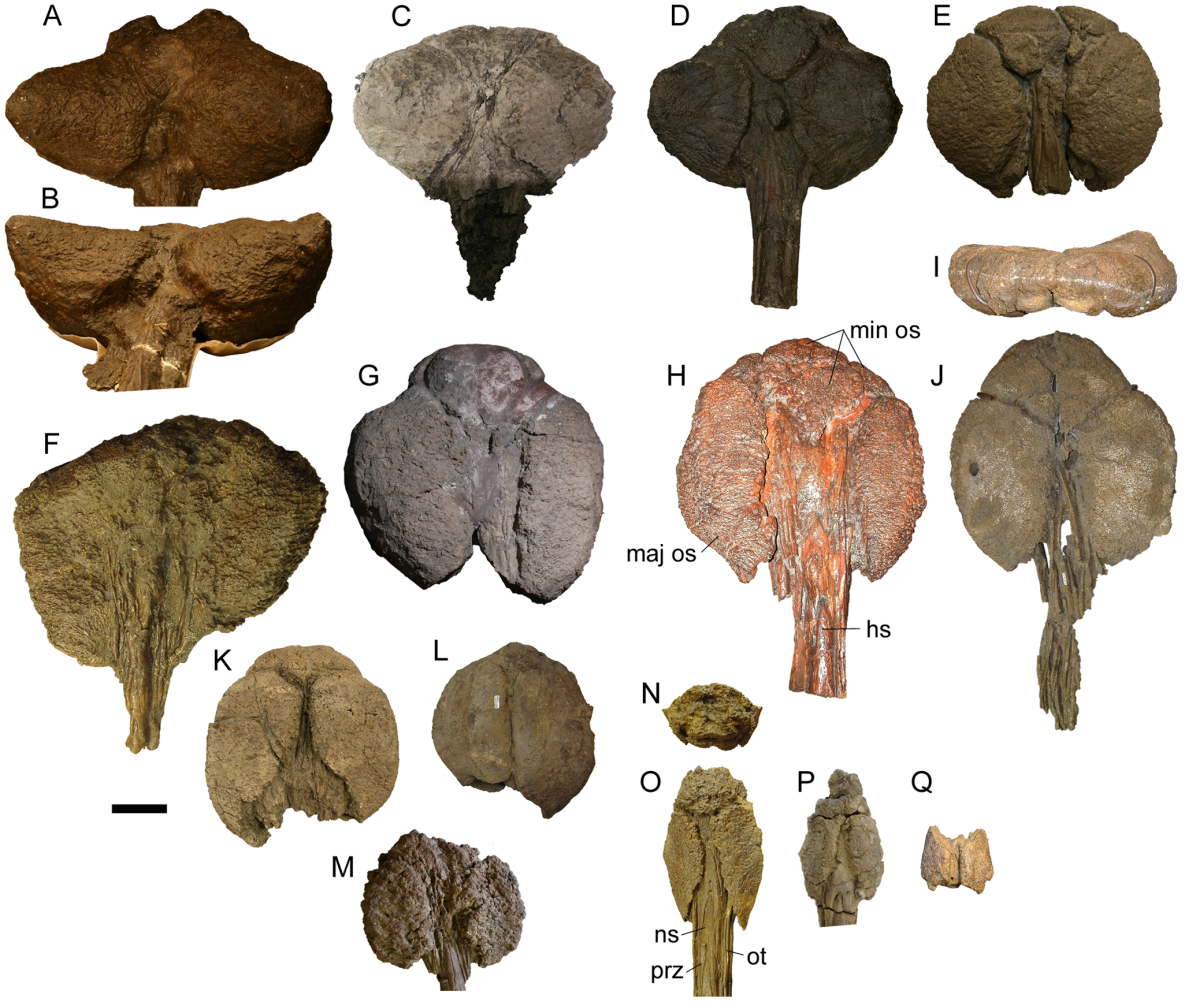
Tail clubs. Tail club knobs from the Horseshoe Canyon Formation in **A–E**: AMNH 5245 in **A**) dorsal and **B**) anterior views; **C**) TMP 1994.168.1 in dorsal view; **D**) AMNH 5216 in dorsal view; **E**) USNM 10753 in dorsal view. Tail club knobs from the Dinosaur Park Formation in **F–Q**: **F**) ROM 788 in ventral view; **G**) MACN Pv 12554 in ventral view; **H**) CMN 349 in ventral view; TMP 1983.36.120 in **I**) posterior and **J**) dorsal views; **K**) UALVP 16247 in dorsal view; **L**) CMN 135 in dorsal view; **M**) TMP 2001.42.9 in dorsal view; ROM 784 (holotype of *Dyoplosaurus acutosquameus*) in **N**) posterior and **O**) dorsal views; **P**) UALVP 47273 in dorsal view; **Q**) ROM 7761 in dorsal view. Scale bar equals 10 cm. Photograph of MACN Pv 12554 taken by E. Snively, photograph of CMN 349 taken by M. Burns, used with permission. Photograph of AMNH 5216 courtesy of the American Museum of Natural History. **Abbreviations:** hs, haemal spine; maj os, major osteoderm of the tail club knob; min os, minor osteoderm of the tail club knob; ns, neural spine; ot, ossified tendon; prz, prezygapophysis.

The tail club (Fig. 14, Tables S6, S10) is one of the most recognizable features of derived ankylosaurids, but has been represented by only a few characters that essentially code for the presence or absence of the tail club. Tail club absent/present (character 173 in [32] and this paper) refers to the presence or absence of terminal osteoderms that envelop the end of the tail (knob osteoderms sensu [10]). Two additional characters define the handle vertebrae (sensu [10]): shape of distal caudal postzygapophyses (character 115) and extent of pre- and postzygapophyses over their adjacent centra in posterior vertebrae (character 116). However, morphological variation in the handle vertebrae and knob osteoderms may have taxonomic and phylogenetic significance. There is always a pair of large osteoderms (major osteoderms sensu [10]), and a variable number of smaller osteoderms that envelop the end of the tail (minor osteoderms sensu [10]). Variations in tail club knob morphology have been noted by Coombs [10], Arbour [59], and Arbour et al. [24]. AMNH 5216, AMNH 5245, and TMP 1994.168.1 are all wider than long, and have relatively pointed, triangular (in dorsal view) major knob osteoderms (Fig. 14A–D). UALVP 47273 is longer than wide and one of the smallest tail club knobs from Alberta; it is similar to the tail club of ROM 784, *Dyoplosaurus acutosquameus* (Fig. 14N–P). The tail club knob of TMP 2001.42.19 (Fig. 14M) is also relatively small, but the length and width are nearly equal, unlike the condition in *Dyoplosaurus*. The major osteoderms of the knob are hemispherical in dorsal view. The distal part of the knob is somewhat damaged, making it difficult to determine how many minor osteoderms were present. The remaining tail clubs are usually equally as wide as long, or slightly longer than wide, and have major knob osteoderms that are semicircular in dorsal view. The number of minor osteoderms forming the terminus of the tail varies among specimens. Keels may be present at the mid-height of each major osteoderm (giving the knob a lenticular cross-section as in CMN 135 and ROM 7761), or near the dorsal surface of each osteoderm (giving the knob a semicircular cross-section as in AMNH 5245 and UALVP 16247).

### Stratigraphic Distribution Of Ankylosaurid Specimens From Alberta And Montana

Ankylosaurid remains have been recovered from several localities in southern Alberta, including the badlands along the Red Deer River from Tolman Bridge to Drumheller, and from the older strata within Dinosaur Provincial Park, to the east near Hilda, and to the south near Manyberries and Onefour (Fig. 1, Table S1, Locality Data S1). Ankylosaurids are represented primarily by isolated teeth in the Milk River [60],[61], Foremost, and Oldman formations, and by more complete material in the Dinosaur Park, Horseshoe Canyon, and Scollard formations [62].

The exact locality for the holotype of *Euoplocephalus tutus* (CMN 0210) is unknown. Field notes by L. Lambe (18 August 1897; CMN) state that it was collected from the east side of the Red Deer River near the mouth of Berry Creek, a region of Dinosaur Provincial Park that is today referred to as the Steveville area. The holotype of *Dyoplosaurus acutosquameus* (ROM 784) was found within a region of Dinosaur Provincial Park known today as the core area, and the location of the quarry is figured in Arbour et al. ([24]: Fig. 2). Good locality data are known for CMN 8530, the holotype of *Anodontosaurus lambei*, which was collected from the Horseshoe Canyon Formation along the Red Deer River, southwest of the town of Morrin.

There is uncertainty regarding the location of the quarry for NHMUK R5161, the holotype of *Scolosaurus cutleri*. Nopcsa [21] gave the location for NHMUK R5161 as one half mile below Happy Jack ferry on the Red Deer River, about halfway up a 400-foot-deep canyon; this information was passed on to Nopcsa from F. A. Bather (NHMUK), who had received this information from W. Parks (ROM), who in turn had received this information from L. Sternberg. W. E. Cutler, who had originally discovered NHMUK R5161, was badly injured during its excavation [63], and so either one or several members of the Sternberg family finished the excavation. The quarry location was marked on the Steveville topographic map [44], but frequent attempts to find the quarry between 1967 and 2007 failed to find a quarry stake. When this locality was visited in 2007, the quarry stake was found downstream and down-section from where it had been marked on the map. Furthermore, it was posted at an angle at the top of a vertical wall, which makes it unlikely that this represents the quarry for NHMUK R5161 (Tanke pers. comm. 2013). GPS coordinates for this quarry stake provided by Currie and Russell [35] were taken from the map position. No photographs of the quarry are known in either museum collections or archives. However, a potential quarry has been located a short distance away from where the original quarry stake was found in 2007, and the skyline matches that in a poor photograph of the quarry that was published in a magazine (Tanke pers. comm. 2013). Unfortunately, no definitive evidence such as newspaper scraps with dates (used to identify ‘lost’ quarries [64]), or ankylosaurid elements, have been recovered, and there is some ambiguity regarding whether or not this quarry lies within the lowest Dinosaur Park Formation or the Oldman Formation (Tanke pers. comm. 2013). Additional fieldwork and research is required to verify the geographic and stratigraphic position of NHMUK R5161. The stratigraphic position for NHMUK R5161 reported in this paper is from Currie and Russell [35], but it should be noted that this specimen may instead have come from the Oldman Formation.

**Figure 15:**
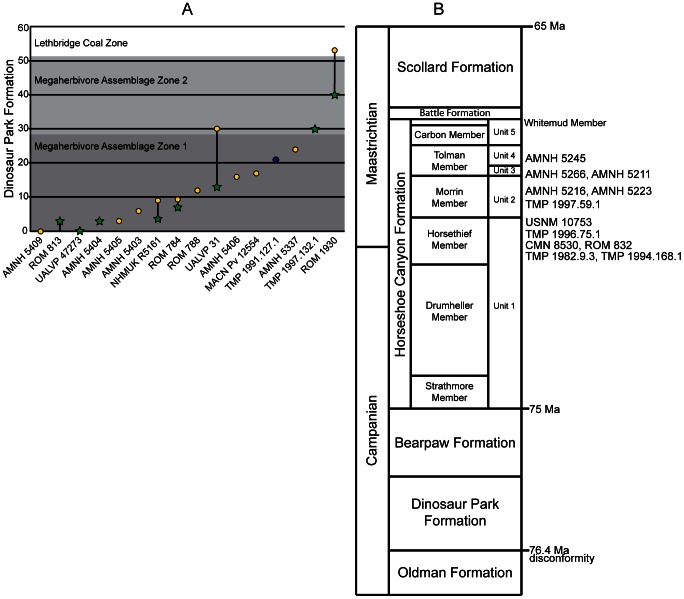
Stratigraphic distribution of ankylosaurid specimens in the Dinosaur Park and Horseshoe Canyon formations of Alberta. **A**) Distribution of ankylosaurid specimens within the Dinosaur Park Formation. Megaherbivore Assemblage Zones after Mallon et al. [18]. Specimens marked with green stars have GPS coordinates and accurate elevation data, specimens marked with yellow dots have elevations estimated from field notes, and the location of TMP 1991.127.1 (marked by a blue hexagon) was estimated from Alberta Township System coordinates. The elevation of some specimens with GPS coordinates was also estimated using field notes and Google Earth; the vertical lines associated with these illustrate the maximum elevation from using field note data only. Specimens marked by yellow dots, as such, could vary in elevation anywhere from three to seventeen meters. ROM 784 is the holotype of *Dyoplosaurus acutosquameus* and NHMUK R5161 is the holotype of *Scolosaurus cutleri*. Although the exact locality and elevation for the holotype of *Euoplocephalus tutus* (CMN 0210) is unknown, AMNH 5406 and UALVP 31 can be confidently referred to that taxon. **B**) Stratigraphic column showing Upper Cretaceous formations in southern Alberta. Nomenclature for the Horseshoe Canyon Formation follows Eberth and Braman [67]. CMN 8530, the holotype of *Anodontosaurus lambei*, occurs in the Horsethief Member.

In the Dinosaur Park Formation, nearly all ankylosaurid specimens that include more than a single isolated element (such as a tooth, isolated caudal vertebra, or osteoderm) have been recovered from within the lowest 30 meters of the formation (Fig. 15A). This is consistent with previous findings [65] that the proportion of ankylosaur teeth in microsite samples decreases in the upper part of the Dinosaur Park Formation. Exceptions to this are ROM 1930 (a skull with partial postcrania), and TMP 1997.132.01 (a skull with partial postcranium). TMP 1997.132.1 was not collected from Dinosaur Provincial Park, but from the area around Hilda, close to the Saskatchewan border. Within Dinosaur Provincial Park, numerous specimens are known from the western and central areas of the park, and fewer are known from the eastern end of the park and from the northern side of the Red Deer River (Locality Data S1).

Two specimens, CMN 8876 and TMP 2000.57.30, were collected between Manyberries and Onefour, near the Alberta-Montana border (Fig. 1). The Dinosaur Park Formation thins in this region, and the Oldman Formation is well exposed. The exact locality is unknown for CMN 8876, so it is not clear if this specimen derives from the Oldman or Dinosaur Park Formation.

Specimens from the Horseshoe Canyon Formation were collected from exposures along the Red Deer River between Tolman Bridge and the Royal Tyrrell Museum of Palaeontology in Drumheller, from Rosebud Creek, and from west of the Red Deer River at the Three Hills Creek Locality (Fig. 1). CMN 8530 and TMP 1994.168.1 were collected between Morrin and the Bleriot Ferry (Fig. 1). In their review of *Anchiceratops*, Mallon et al. [66] noted that specimens collected in this region occur within a large sediment package that includes Coal Seams 8 and 9, in the upper part of Unit 1 of the Horseshoe Canyon Formation, now defined as the Horsethief Member [67]. TMP 1982.9.3 was collected from Fox Coulee, between Coal Seams 7 and 8 (Eberth pers. comm.), placing this specimen within the Horsethief Member. AMNH 5266 and USNM 10753 were collected north of Morrin, and the original field notes do not include any distinctive lithostratigraphic or palaeontological features. However, these ankylosaur specimens occur south of *Anchiceratops* specimens that had good stratigraphic data constraining them to Unit 2 (Morrin Member sensu [67]) of the Horseshoe Canyon Formation [66], and north of *Anchiceratops* and ankylosaur specimens that are likely in the upper part of Unit 1 (Horsethief Member sensu [67]). AMNH 5266 and USNM 10753 were thus probably collected in the Horsethief or Morrin members of the Horseshoe Canyon Formation. AMNH 5211, 5216, 5223, and 5245 were collected between 2.4 km (1.5 mi) upstream and 5.6 km (3.5 mi) downstream of the Tolman Bridge (previously the Tolman Ferry). Unfortunately, the exact localities for these specimens are unknown, but the Morrin, Tolman and Carbon members (sensu [67]) of the Horseshoe Canyon Formation crop out in this region of the Red Deer River [66]. Two specimens were collected from localities other than those along the Red Deer River. TMP 1996.75.1 was collected from Three Hills Creek, from the Horsethief Member (Eberth pers. comm.), and TMP 1997.59.1 was collected from Rosebud Creek, from the Morrin Member (Eberth pers. comm.).

Only one species, *Ankylosaurus magniventris,* is found in the Scollard Formation in Alberta and no other ankylosaurids appear to have been contemporaneous with this taxon. At present, no definitive ankylosaurid fossils have been recovered from Judithian sediments in Alberta anywhere north of Dry Island Buffalo Jump Provincial Park, including the fossiliferous localities in the city of Edmonton, or around Grande Prairie in northwestern Alberta.

Trexler [68] noted the presence of cf. *Euoplocephalus* at two localities (Landslide Butte and Two Medicine River) in the Two Medicine Formation and fragmentary ankylosaurid remains from the Choteau/Bynum locality. At all three localities, ankylosaurids were only recovered from the upper part of the Two Medicine Formation. MOR 433 was collected from approximately 55 m below the contact with the Bearpaw Shale [25].

## Discussion

### Taxonomic Implications Of Variation In Specimens Previously Referred To *Euoplocephalus Tutus*

#### Status Of Anodontosaurus Lambei

Variation within a population can result from ontogenetic differences, individual differences (both heritable and acquired), sexual dimorphism, and pathologies (and, additionally for fossil organisms, from taphonomic changes). If *Euoplocephalus tutus* is monotypic, there should be no clusters of shared, distinctive morphological features, (unless there is sexual dimorphism) and there should be no stratigraphic separation of groupings of morphological features. A review of morphological variation in specimens previously referred to *Euoplocephalus tutus* shows that certain features previously considered to result from individual variation are associated with each other, and are stratigraphically separated (Table 2). These features include the presence or absence of small circular caputegulae at the base of the squamosal and quadratojugal horns (postocular caputegulae, Fig. 5), the presence or absence of similar small circular osteoderms (interstitial osteoderms) around the primary osteoderms of the first cervical half ring (Fig. 13), the width:length ratio of the tail club knob, and the shape (semicircular vs. triangular) of the tail club knob osteoderms in dorsal view (Fig. 14).

**Table 2:** Presence or absence of diagnostic features in *Anodontosaurus*, *Dyoplosaurus*, *Euoplocephalus*, and *Scolosaurus*.

Specimen #	Squamosal horn shape	Postocularcaputegulae absent/present	Interstitial osteodermson first cervical half ring absent/present	First cervical halfring medialosteoderm shape	Tail club knob shape,width:length	Formation	Taxonomic Assignment, this paper
AMNH 5216	X	x	x	x	pointed	HCF, Morrin Mbr	*Anodontosaurus lambei*
AMNH 5223	?	present	x	x	x	HCF, Morrin Mbr	*Anodontosaurus lambei*
AMNH 5238	rounded, straight	present	x	x	x	DPF	*Anodontosaurus lambei*
AMNH 5245	x	x	x	x	pointed, 1.88	HCF, Tolman Mbr	*Anodontosaurus lambei*
AMNH 5337	blunt	absent	absent	keeled	x	DPF, MAZ-1	*Euoplocephalus tutus*
AMNH 5403	blunt	absent	absent	keeled	round	DPF, MAZ-1	*Euoplocephalus tutus*
AMNH 5404	rounded, straight	absent	absent	keeled	x	DPF, MAZ-1	*Euoplocephalus tutus*
AMNH 5405	pointed, straight	absent	absent	keeled	round	DPF, MAZ-1	*Euoplocephalus tutus*
AMNH 5406	x	x	absent	keeled	x	DPF, MAZ-1	*Euoplocephalus tutus*
CMN 135	x	x	x	x	round, 0.74	DPF	Ankylosauridae
CMN 210	x	x	absent	keeled	x	DPF	*Euoplocephalus tutus*
CMN 349	x	x	x	x	round	DPF	Ankylosauridae
CMN 8530	blunt	present	present	? (eroded)	x	HCF, Horsethief Mbr	*Anodontosaurus lambei*
FPDM V-31	pointed, backswept	? (reconstructed)	x	x	x	Two Medicine	*Scolosaurus cutleri*
MACN Pv 12554	x	x	x	x	round	DPF, MAZ-1	Ankylosauridae
MOR 433	rounded, backswept	present	x	x	x	Two Medicine	*Scolosaurus cutleri*
NHMUK R4947	blunt	present	x	x	x	Alberta, unknownformation	*Anodontosaurus lambei*
NHMUK R5161	x	x	absent	flat, low centralprominence	x	*DPF, MAZ-1	*Scolosaurus cutleri*
NSM PV 20381	pointed, backswept	? (reconstructed)	x	x	x	Two Medicine	*Scolosaurus cutleri*
ROM 784	x	x	x	x	elongate, 0.69	DPF, MAZ-1	*Dyoplosaurus acutosquameus*
ROM 788	x	x	x	x	round, 1.1	DPF, MAZ-1	Ankylosauridae
ROM 832	rounded, straight	present	x	x	x	HCF, Horsethief Mbr	*Anodontosaurus lambei*
ROM 1930	rounded, straight	absent	x	x	x	DPF, MAZ-2	*Euoplocephalus tutus*
TMP 1983.36.120	x	x	x	x	round, 0.86	DPF; MAZ?	Ankylosauridae
TMP 1991.127.1	pointed, straight	absent	x	x	x	DPF, MAZ-1	*Euoplocephalus tutus*
TMP 1994.168.1	x	x	x	x	pointed, 1.35	HCF, Horsethief Mbr	*Anodontosaurus lambei*
TMP 1996.75.1	rounded, straight	present	present	?	x	HCF, Horsethief Mbr	*Anodontosaurus lambei*
TMP 1997.132.1	blunt	present	present	? (uncertain position)	x	DPF, MAZ-2	*Anodontosaurus lambei*
TMP 1997.59.1	rounded, straight	present	x	x	x	HCF, Morrin Mbr	*Anodontosaurus lambei*
TMP 2001.42.19	pointed, backswept	present	absent	flat, low centralprominence	round, 1.09	Two Medicine	*Scolosaurus cutleri*
UALVP 31	pointed, straight	absent	absent	keeled	x	DPF, MAZ-1	*Euoplocephalus tutus*
UALVP 16247	x	x	x	x	round, 0.86	DPF	Ankylosauridae
UALVP 47273	x	x	x	x	elongate, 0.62	DPF, MAZ-1	*Dyoplosaurus acutosquameus*
USNM 7943	x	x	absent	flat, low centralprominence	x	Two Medicine	*Scolosaurus cutleri*
USNM 10753	x	x	x	x	round, 1.07	HCF, Horsethief Mbr	*Anodontosaurus lambei*
USNM 11892	pointed, backswept	present	x	x	x	Two Medicine	*Scolosaurus cutleri*

Abbreviations: DPF, Dinosaur Park Formation; HCF - Horseshoe Canyon Formation, MAZ - Megaherbivore Assemblage Zone (sensu 18). Horseshoe Canyon Formation members sensu Eberth and Braman [67].

doi:10.1371/journal.pone.0062421.t002

Specimens previously referred to *Euoplocephalus tutus* that lack postocular caputegulae never have interstitial osteoderms on the first cervical half ring (Table 2). Specimens referred to *Euoplocephalus tutus* that have postocular caputegulae may or may not have interstitial osteoderms on the first cervical half ring. This is a subtle difference and could be attributed to intraspecific variation; however, the presence or absence of small caputegulae near the squamosal and quadratojugal horns and on the cervical half rings correlates with the stratigraphic position of the specimen. All specimens from the Horseshoe Canyon Formation have small caputegulae at the bases of the squamosal and quadratojugal horns and interstitial osteoderms on the cervical half rings (these are visible even on highly fragmentary cervical half rings such as the one preserved with TMP 1982.9.3). Only two specimens from the Dinosaur Park Formation have these small caputegulae: AMNH 5238, from Dinosaur Provincial Park, and TMP 1997.132.1, from the area around Hilda, Alberta (near the Alberta-Saskatchewan border). TMP 1997.132.1 is from the upper 30 m of the Dinosaur Park Formation, but the stratigraphic position of AMNH 5238 is unknown. No specimens from the lower 30 m of the Dinosaur Park Formation have small caputegulae at the bases of the squamosal and quadratojugal horns or secondary osteoderms on the cervical half rings. The stratigraphic separation of the presence or absence of these caputegulae and osteoderms suggests that specimens from the Horseshoe Canyon Formation are not the same species as those from the lower part of the Dinosaur Park Formation. TMP 2001.49.2, from the Two Medicine Formation of Montana, has postocular osteoderms on the skull, but does not have interstitial osteoderms on the first cervical half ring; an isolated half ring (USNM 7943) from the Two Medicine Formation also lacks interstitial osteoderms.

The size and shape of the tail club knob (Fig. 14, Table S10) varies significantly among specimens referred to *Euoplocephalus tutus*, as reviewed by Coombs [10]. However, tail club knobs that are wider than long (AMNH 5216, AMNH 5245; Figs. 14A, D) also tend to have major knob osteoderms that are triangular (“bluntly pointed” sensu [10]) in dorsal view. Tail club knobs that are longer than wide (UALVP 47273; Fig. 14P) or approximately as wide as long (TMP 1983.36.120, TMP 2001.49.2; Figs. 14J, M) have major knob osteoderms that are semicircular in dorsal view. Again, these differences are stratigraphically separated, with wide, pointed tail club knobs found in the Horseshoe Canyon Formation, and round or elongate, semicircular tail club knobs found in the Dinosaur Park and Two Medicine formations. Differences in proportions are not entirely related to absolute size, as both ROM 788 (from the Dinosaur Park Formation) and AMNH 5245 (from the Horseshoe Canyon Formation) are almost the same width, but AMNH 5245 is not as long as ROM 788 (Table S10). Unfortunately, no tail club knobs from the Horseshoe Canyon Formation are associated with cranial material. Only a few tail club knobs from the Dinosaur Park Formation have associated cranial material: AMNH 5403, AMNH 5405, and the holotype of *Dyoplosaurus acutosquameus*, ROM 784.

The morphology of osteoderms, and their distribution on the body, is known to vary in several extant animals. The number of moveable thoracic carapace segments in several species of armadillos can vary by one to three bands; in the nine-banded armadillo (*Dasypus novemcinctus*), variation in number of segments is associated with geographic occurrence [69]. The number of osteoderms in the cervical region of crocodilians differs between species, but also varies among individuals of each species [70]. Ornamentation on the osteoderms of the broad-headed skink *Eumeces laticeps* increases with an increase in body size [71]. Given the documented variation in extant taxa, caution should be used when identifying potential taxonomically useful features in the osteoderms and cranial ornamentation of ankylosaurs. Some caputegulae positions and shapes are consistent in all known skulls previously referred to *Euoplocephalus*: all skulls have rugose, arched supranarial caputegulae, all have a roughly hexagonal median nasal plate that is larger than all of the other frontonasal caputegulae, and all have rectangular lacrimal and loreal caputegulae. Variation in cranial ornamentation also exists within specimens previously referred to *Euoplocephalus*, most notably in the number, shapes, and sizes of the caputegulae of the frontals, parietals, and nasals. However, discontinuous, stratigraphically-separated variation in the presence or absence of postocular caputegulae, the presence or absence of interstitial osteoderms on the first cervical half ring, and tail club knob osteoderm shape, is more likely the result of taxonomic variation rather than intraspecific or ontogenetic variation in *Euoplocephalus*.

The stratigraphic separation of unique sets of morphological features in specimens referred to *Euoplocephalus tutus* indicates that more than one species is currently represented by material referred to *Euoplocephalus tutus*. Although the exact type locality for *Euoplocephalus tutus* (CMN 210) is unknown, the localities for AMNH 5406 and UALVP 31 (which can be confidently referred to *Euoplocephalus tutus* based on cervical half ring morphology) are known, and both are from the lower Dinosaur Park Formation. The skull of UALVP 31 does not have postocular caputegulae (Fig. 5), and the cervical half rings of AMNH 5406, CMN 0210, and UALVP 31 do not have interstitial osteoderms (Fig. 13). As such, the Horseshoe Canyon Formation morphotype, which has these caputegulae and osteoderms, should not be referred to *Euoplocephalus tutus*. The holotype of *Anodontosaurus lambei* (CMN 8530) was collected from section 3, township 21, range 31, W 4^th^ Meridian, placing this specimen within the Horseshoe Canyon Formation. CMN 8530 has postocular caputegulae at the base of the squamosal and quadratojugal horns (Fig. 5) and interstitial osteoderms on the first cervical half ring (Fig. 13). The presence of these caputegulae and osteoderms on the skull and cervical half ring suggest that *Anodontosaurus lambei* is distinct from *Euoplocephalus tutus*, and specimens from the Horseshoe Canyon Formation should be referred to *Anodontosaurus lambei* rather than *Euoplocephalus tutus*. It could be argued that these differences are insufficient for resurrecting the genus *Anodontosaurus*, and instead the Horseshoe Canyon Formation specimens should be referred to as a second species of *Euoplocephalus*, *E. lambei*. Given that the phylogenetic resolution of derived ankylosaurids is poor at present ([32],[72], this paper), it is here considered best to simply resurrect *Anodontosaurus lambei* rather than create additional taxonomic confusion by creating a new combination.

Although no tail club knobs are associated with diagnostic cranial material, the consistent morphology of tail club knobs from the Horseshoe Canyon Formation suggests that a single taxon is represented, and so it is best to refer them to *Anodontosaurus lambei* as well. As such, *Anodontosaurus lambei* also differs from *Euoplocephalus tutus* in the morphology of the tail club knob. In *Anodontosaurus lambei*, the tail club knob is wider than long, and the major knob osteoderms are bluntly pointed and triangular in dorsal view (Fig. 14).

Another potential difference between *Anodontosaurus lambei* and specimens referred to *Euoplocephalus tutus* is the shape of the free caudal vertebrae. CMN 8530 includes a free caudal vertebra that differs from ankylosaurid free caudal vertebrae from the Dinosaur Park Formation, as the centrum has octagonal anterior and posterior faces, rather than circular (Fig. 9K). The only other specimen from the Horseshoe Canyon Formation that preserves free caudal vertebrae is AMNH 5245, and in this specimen the vertebrae are pathological and their original shapes are obscured. It is therefore not possible to determine if the centrum shape in CMN 8530 represents a taxonomic difference or individual variation, although it should be noted that centrum shape does not appear to vary among specimens from the Dinosaur Park Formation.

Although the sample size is small, femoral morphology appears to differ between specimens from the Horseshoe Canyon Formation and Dinosaur Park Formation (Fig. 12). In the femora of AMNH 5266 and TMP 1982.9.3 from the Horseshoe Canyon Formation (Figs. 12C, D), the lateral epicondyles are more prominent than that of AMNH 5404, from the Dinosaur Park Formation (Fig. 12K). This does not appear to be size-related, as the lateral epicondyle is proportionately larger in AMNH 5266 even though this femur is less than half the length of AMNH 5404.

Within the Dinosaur Park Formation, most specimens that include more than just teeth or isolated osteoderms have been collected from the lowest 30 m of the formation. Two notable exceptions to this are ROM 1930 and TMP 1997.132.1, which were collected from the upper 30 m of the formation. TMP 1997.132.1 has postocular caputegulae, but ROM 1930 does not (Fig. 5), and, TMP 1997.132.1 has interstitial osteoderms on the first cervical half ring (Fig. 13O). Based on the presence of these osteoderms, TMP 1997.132.1 is referred to *Anodontosaurus lambei*, which extends the stratigraphic range of this species into the upper Dinosaur Park Formation. This makes TMP 1997.132.01 by far the most complete specimen of *Anodontosaurus lambei*, as this specimen includes a complete skull, right mandible, three dorsal vertebrae, ribs, scapula, left humerus, ulna, radius, and tibia.

AMNH 5266, a partial juvenile skeleton, was referred to *Euoplocephalus tutus* by Coombs [9]; because Coombs had previously synonymized *Anodontosaurus lambei* with *Euoplocephalus tutus*, his comparison focused on differences between *Ankylosaurus magniventris* and *Euoplocephalus tutus* only. This specimen derives from either the Morrin or Tolman member of the Horseshoe Canyon Formation, and thus is most likely referable to *Anodontosaurus lambei*. AMNH 5266 lacks a skull, first cervical half ring, and tail club, and so it preserves no diagnostic features of *Anodontosaurus lambei*. However, the femur has a prominent lateral epicondyle, similar to that of TMP 1982.9.3 but different from that of AMNH 5404 (Fig. 12). Because this feature is subtle and the sample size is limited, the relative prominence of the lateral epicondyle is not here considered a diagnostic feature. However, the similarity of the femora of AMNH 5266 and TMP 1982.9.3 suggests that AMNH 5266 can be referred to *Anodontosaurus*.

#### Status Of *Scolosaurus Cutleri*

With the recognition of *Dyoplosaurus acutosquameus* [24], and now *Anodontosaurus lambei* as species distinct from *Euoplocephalus tutus*, only *Scolosaurus cutleri* remains from the list of taxa synonymized by Coombs [7]. Penkalski and Blows [34] have argued for the separation of *Scolosaurus* from *Dyoplosaurus* and *Euoplocephalus* based on several morphological features. The holotype of *Scolosaurus cutleri* (NHMUK R5161) is one of the most complete ankylosaurs ever collected, preserving nearly the entire skeleton as well as *in situ* osteoderms and skin impressions. However, it is challenging to compare this specimen with other specimens for several reasons. First, it lacks a skull and tail club, which contain important taxonomic information. Second, although the *in situ* osteoderms and skin impressions provide important information on the integument of ankylosaurs, they also obscure certain skeletal elements such as the scapula and pelvis. Third, the specimen is currently on display tipped onto its right side, in a relatively dark area, in a glass cabinet that cannot be easily opened, which makes detailed examination of the specimen difficult, especially the anterior and left side of the animal. Nevertheless, it is possible to assess the taxonomic status of NHMUK R5161 as it preserves the first cervical half ring, and thus can be compared to both *Anodontosaurus lambei* and *Euoplocephalus tutus*.

The first cervical half ring of NHMUK R5161 (Fig. 13P) lacks interstitial osteoderms ringing the larger primary osteoderms, which indicates that NHMUK R5161 is not referable to *Anodontosaurus lambei*. Although these may appear to be present on the second cervical half ring, these are epidermal scales and not osteoderms (see [34]). NHMUK R5161 differs subtly from *Euoplocephalus tutus* (AMNH 5406, CMN 0210, and UALVP 31) in the shape of the first cervical ring osteoderms, as it has low medial osteoderms, each of which lacks a distinct keel but has a low, somewhat posteriorly placed prominence (Fig. 13P). The lateral osteoderms appear to have a prominent, laterally-directed keel. In contrast, AMNH 5406, CMN 0210, and UALVP 31 have tall medial osteoderms with prominent keels (Fig. 13A, B, F, G). Some referred *Euoplocephalus tutus* first cervical half rings (AMNH 5337, AMNH 5404) also have low medial osteoderms (Fig. 13J–L), but in these specimens the medial osteoderms still have a keel, and the lateral osteoderms are also low, which differs from the condition in NHMUK R5161 where the lateral osteoderms are tall.

Penkalski and Blows [34] also note differences in shape between the medial osteoderms of NHMUK R5161 and other referred *Euoplocephalus* specimens. They point out that the anteroposterior length of the cervical half ring band was larger in NHMUK R5161 than in *Euoplocephalus* specimens AMNH 5406 and UALVP 31. However, the humerus of NHMUK R5161 is also larger than those of AMNH 5406 and UALVP 31 (Table S7), and so the greater anteroposterior band length in NHMUK R5161 may simply be a result of NHMUK R5161 being a larger individual than either AMNH 5406 or UALVP 31.

The first cervical half ring in NHMUK R5161 is not as well preserved as that of the second half ring, which at first seems to differ greatly from second cervical half rings referred to *Euoplocephalus tutus* (AMNH 5403, TMP 2007.12.52). No other second cervical half rings referred to *Euoplocephalus tutus* preserve the superficial primary osteoderms (Fig. 13U, V), but these are present on NHMUK R5161 (Fig. 13P). However, it appears that the cervical half ring osteoderms do not always fuse to the band; matrix separates the osteoderms from the band in the first cervical half ring of UALVP 31 (Fig. 13G). As such, the presence or absence of osteoderms on the second cervical half ring is not taxonomically informative. The morphology of the osteoderms on the second cervical half ring in NHMUK R5161 can, however, be used to corroborate the morphology of the more poorly preserved first cervical half ring. In the ankylosaurids *Pinacosaurus mephistocephalus* [33], *Saichania chulsanensis* (MPC 100/151), and *Shamosaurus scutatus* Tumanova, 1983 [73] (PIN 3779/2), the first and second cervical half rings are nearly identical except in terms of overall size. In the second cervical half ring of NHMUK R5161, the medial osteoderms are nearly flat with low posterior prominences and circular bases, and the lateral osteoderms are tall and sharply keeled, a morphology unknown in any other referred *Euoplocephalus tutus* half ring from Alberta. The morphology of the cervical half rings in NHMUK R5161 supports the Penkalski and Blows [34] interpretation of *Scolosaurus cutleri* as a species distinct from *Euoplocephalus tutus*, and also separates it from *Anodontosaurus lambei.* As discussed for *Anodontosaurus lambei*, it is preferred to maintain separation at the generic level rather than creating the new combination *E. cutleri*.

*Scolosaurus cutleri* can also be differentiated from *Dyoplosaurus acutosquameus* by the orientation of the anterior sacral ribs, which are anteroventrally directed in *Dyoplosaurus* but laterally directed in *Scolosaurus*. NHMUK R5161 also has a proportionally longer postacetabular process of the ilium. *Scolosaurus cutleri* may also have incorporated more caudals into the sacrum (but not necessarily sacral rod – caudosacrals may not fuse at the centra, but their transverse processes fuse to the ilium) compared to *Anodontosaurus lambei*, *Dyoplosaurus acutosquameus*, and specimens referred to *Euoplocephalus tutus*. NHMUK R5161 has three caudosacrals, whereas *Dyoplosaurus* ROM 784 preserves two, and AMNH5337 and AMNH5409 each preserve one (the sacra for AMNH 5245 and TMP 1982.9.3 are incomplete). However, it is unclear if the number of caudosacrals is associated with absolute size or ontogeny. The pelves of AMNH 5409 and NHMUK R5161 are nearly the same length (length of ilium in NHMUK R5161 = 96 cm, from [21]; length along midline of pelvis in AMNH 5409 = 92 cm). ROM 1930 may have had three caudosacrals, but these are not preserved in association with a complete pelvis, so it is not possible to determine for certain if these vertebrae were fused to the ilia. It is possible that fewer sacral vertebrae are present in specimens other than NHMUK R5161 because of post-depositional damage, although this seems unlikely for ROM 784 (*Dyoplosaurus acutosquameus*), which has a complete, articulated caudal series. At present, the number of dorsosacral, sacral, and caudosacral vertebrae cannot be used to support taxonomic distinctions among Albertan and Montanan ankylosaurids.

Penkalski and Blows [34] observed differences in the humeri and radii of AMNH 5406 and NHMUK R5161: AMNH 5406 is smaller, the deltopectoral crest does not extend as far down the shaft as that in NHMUK R5161, and the radial condyle extends farther distally than in other specimens (although these other specimens are not specified in [34]). The deltopectoral crest of AMNH 5406 (Fig. 11A) does not extend as far down the shaft as in AMNH 5337, a larger specimen, but seems to extend proportionately as far in ROM 47655 (Fig. 11K), the largest humerus encountered in this study. The radial condyle does extend somewhat further distally compared to AMNH 5404 (Fig. 11I), but is again similar to ROM 47655. It should be stressed that variations in the extent of the deltopectoral crest and radial condyle are both subtle, and size should not be used as a diagnostic character in the absence of ontogenetic data. For this reason, there is no reason to consider the morphology of the humerus in NHMUK R5161 significantly different than that of other referred *Euoplocephalus* specimens. As such, humeral morphology is not diagnostic for *Scolosaurus*. Penkalski and Blows [34] considered the radius of NHMUK R5161 to be more sigmoidal than those of any other referred *Euoplocephalus* specimens, or than that in *Dyoplosaurus*. The radius as figured by Nopcsa ([21]: plate VI) does have a weakly sigmoidal appearance that differs from the radii of AMNH 5337 (Fig. 11L) and AMNH 5406 (Fig. 11M), and so this may be a diagnostic character of NHMUK R5161.

NHMUK R5161 differs from ROM 784 (*Dyoplosaurus*) in the morphology of the pedal unguals, which are U-shaped in ventral view in NHMUK R5161 and triangular in ROM 784. *Scolosaurus* may also differ from *Dyoplosaurus* in the morphology and pattern of post-cervical osteoderms [34]. ROM 784 has triangular osteoderms on the lateral sides of the posterior region of the pelvis and anterior part of the tail, which are not present in NHMUK R5161. Although the integument is fairly complete dorsally in NHMUK R5161, osteoderms are not preserved lateral to the caudal vertebrae, and so it is possible that compressed, triangular osteoderms were present in NHMUK R5161 but not preserved.

#### Status Of *Oohkotokia Horneri*

Penkalski [25] identified several diagnostic features that separated ankylosaurids from the Two Medicine Formation from *Dyoplosaurus*, *Euoplocephalus* (including *Anodontosaurus*), and *Scolosaurus*: a proportionately small median nasal caputegulum not distinguished from surrounding caputegulae; keeled, trihedral squamosal horns with posteriorly-situated apices; quadratojugal horns with strong posterior curvature; nuchal crest not visible in lateral view; small occipital condyle; large orbit; basally excavated osteoderms with weakly ornamented surface texture; and steeply-pitched triangular caudal osteoderms.

Penkalski [25] emphasized the small median nasal caputegulum of specimens from the Two Medicine Formation as an important difference between *Oohkotokia* and *Euoplocephalus*. In all specimens referred to *Oohkotokia*, the anterior portion of the rostrum is broken, and so the median nasal caputegulum is either absent or only partially preserved. Although FPDM V-31, NSM PV 20381, and TMP 2001.42.19 appear to have complete skulls, the anterior portion of the rostrum in each of these specimens is reconstructed. The morphology of the median nasal plate does not provide strong evidence for the separation of the Two Medicine Formation specimens from *Euoplocephalus*. However, the distinctive morphology of the squamosal horns of the Two Medicine Formation ankylosaurids noted by Penkalski [25] differentiates the Two Medicine ankylosaurid from *Euoplocephalus*, and from *Anodontosaurus*. Skulls from the Two Medicine Formation share one feature that is not present in any other specimen referred to *Euoplocephalus* –a long, pointed, back-swept squamosal horn (Fig. 5M–P). As such, all of the skulls from this formation likely represent a single taxon. Although the squamosal horns of AMNH 5405, TMP 1991.127.1, and UALVP 31 are pointed (Fig. 5A–C), they are never as long as those in specimens from the Two Medicine Formation. No specimens from Alberta have the characteristic back-swept appearance in lateral view that is present in specimens from Montana. The Two Medicine ankylosaurid can be differentiated from *Dyoplosaurus* based on the morphology of the pedal unguals (U-shaped in dorsal view in TMP 2001.42.9, triangular in ROM 784).

Penkalski [25] noted two main differences between MOR 433 and NHMUK R5161 (*Scolosaurus*). First, the transverse processes were proportionately longer relative to centrum width in MOR 433 compared to NHMUK R5161. Furthermore, NHMUK R5161 does not preserve any low-keeled oval osteoderms or steeply pitched triangular osteoderms, two morphologies that were found associated with the holotype skull of MOR 433 (see [25]: Fig. 4D and F). In MPC 100/1305, a Mongolian ankylosaurid that preserves numerous *in situ* osteoderms, low-keeled oval osteoderms with off-centre keels are found only on the lateral sides of the trunk, and steeply pitched triangular osteoderms are found only on the lateral sides of the pelvis and tail. Low-keeled osteoderms are found on the dorsal side of the trunk and tail (see [74]). Although NHMUK R5161 preserves most of the dorsal integument, it does not preserve osteoderms on the flanks or lateral sides of the tail, and so it is conceivable that the absence of the unique MOR 433 osteoderm morphologies in NHMUK R5161 is a preservational artifact. The length of the transverse processes relative to the width of the centrum varies along the caudal vertebral column in ankylosaurids, with transverse processes decreasing in size posteriorly. In order to demonstrate that the relatively longer transverse process in MOR 433 is a taxonomic difference and not a positional difference, the position of this caudal vertebra would need to be known so it could be compared to the equivalent position in NHMUK R5161.

Neither osteoderm morphology nor vertebral proportions provide compelling evidence to separate *Oohkotokia* from *Scolosaurus*. However, *Oohkotokia* and *Scolosaurus* share a cervical half ring morphology that differs markedly from those of *Anodontosaurus* and *Euoplocephalus*. TMP 2001.42.19, from the Two Medicine Formation of Montana, includes a partial first cervical half ring (Fig. 13Q, R) that is similar to the cervical half rings of NHMUK R5161 (Fig. 13P), and an isolated half ring from the Two Medicine Formation, USNM 7943 (Fig. 13S, T), shares this morphology. In both of the Two Medicine specimens and *Scolosaurus*, the medial osteoderms are nearly flat and each has a low central prominence and a circular base. In contrast, the first cervical half rings of AMNH 5406, CMN 0210, UALVP 31, and all other referred *Euoplocephalus* half rings have medial osteoderms with longitudinal keels, even those in which the medial osteoderms are relatively low (e.g. AMNH 5404). (Gilmore [75] also remarked on the differences in osteoderm morphology between USNM 7943 and the holotype of *E. tutus*, CMN 0210.) The first cervical half ring of *Anodontosaurus* has small interstitial osteoderms that are not present in any Two Medicine specimens. There are no features that differ significantly between the Two Medicine Formation specimens and *Scolosaurus*, and for this reason the Two Medicine ankylosaur is best referred to *Scolosaurus*. TMP 2001.42.19 includes both a skull and tail club, which are both absent in the holotype of *Scolosaurus*.

TMP 2001.42.19 provides insight into the growth of the tail club knob and variation of ankylosaurid knobs. The maximum width across the supraorbitals in TMP 2001.42.19 is 26 cm, and the maximum width of the knob is 31 cm. In contrast, the preserved portion of the skull of ROM 784 (*Dyoplosaurus*) has a maximum width across the supraorbitals of 33 cm, and the tail club knob maximum width is 16.6 cm. TMP 2001.42.1 is a smaller individual than ROM 784 yet has a larger tail club knob (Fig. 14M, O); the ratio of knob width to length also differs between the two specimens (1.07 in TMP 2001.42.1 vs. 0.68 in ROM 784). This suggests that the small knob and low width:length ratio of *Dyoplosaurus* may not be entirely due to ontogeny, as a larger knob is known in a smaller individual of *Scolosaurus*. An alternate explanation is that the timing of knob osteoderm growth occurred later in *Dyoplosaurus* relative to *Scolosaurus*. However, even if the knobs of both taxa eventually grew to equivalent sizes, the difference in the timing of growth is an interesting taxonomic difference.

#### Status Of Other Specimens Previously Referred To *Euoplocephalus Tutus*

Although numerous well-preserved skulls have been referred to *Euoplocephalus tutus*, none of the holotypes of the Dinosaur Park Formation ankylosaurids (*Euoplocephalus tutus*, *Dyoplosaurus acutosquameus*, and *Scolosaurus cutleri*) include good cranial material. This makes the referral of skulls to any given species difficult, and means that postcranial elements must be used to identify specimens to species level. However, non-overlapping postcranial material among the holotype specimens also makes this challenging. Each of the holotypes of *Anodontosaurus lambei*, *Euoplocephalus tutus* and *Scolosaurus cutleri* includes a first cervical half ring, but none is preserved in *Dyoplosaurus acutosquameus*. *Dyoplosaurus acutosquameus* and *Scolosaurus cutleri* both include pelvic and anterior caudal regions, but *Scolosaurus cutleri* does not preserve the tail club; the holotype of *Euoplocephalus tutus* preserves no postcrania other than the first cervical half ring. AMNH 5406 and UALVP 31 can be referred to *Euoplocephalus tutus* based on cervical half ring morphology, and UALVP 31 includes a good skull. The skull of TMP 1991.127.1 is nearly identical to that of UALVP 31 and so can also be confidently referred to *Euoplocephalus*; each skull even has a distinct shallow furrow on the posterior supraorbital (Fig. 3; Figs. S2, S7).

The morphology of the pelvis can be used to differentiate *Dyoplosaurus acutosquameus* and *Scolosaurus cutleri*, and potentially *Euoplocephalus tutus* as well. The pelves of AMNH 5337 and AMNH 5409 differ from the pelvis of *Dyoplosaurus acutosquameus* in the orientation of the sacral transverse processes, which are anteroventrally directed in *Dyoplosaurus* but laterally directed in AMNH 5337 and AMNH 5409. AMNH 5337 and AMNH 5409 differ from NHMUK R5161 (*Scolosaurus*) in the relative length of the postacetabular process of the ilium. AMNH 5337 includes a skull that is generally similar to that of UALVP 31, but does have some notable differences. In particular, the squamosal horns of AMNH 5337 are much shorter and more rounded, and the cranial caputegulae are less distinct, compared to those of UALVP 31 (Figs. 3, 4). Coombs [4] noted that smaller skulls had more prominent and pointed squamosal and quadratojugal horns compared to larger skulls. Penkalski [13], in a morphometric analysis of referred *Euoplocephalus tutus* skulls, found that squamosal horn height decreased with increasing skull size. He also reported a positive correlation between skull size and rugosity of osteoderm sculpturing. If squamosal horn length and bluntness, and cranial caputegulum distinctness, are related to size, then they are probably a result of ontogenetic changes. Horner and Goodwin [76], in a discussion of ontogeny in the pachycephalosaurids *Dracorex*, *Stygimoloch*, and *Pachycephalosaurus*, suggested that the pyramidal nodes on the nasals and the squamosal horns of these taxa decreased in size and became more rounded through ontogeny. Scannella and Horner [77] also suggested that the epoccipitals of *Triceratops* become lower, and less distinct from the frill throughout ontogeny. It is possible that cranial ornamentation in *Euoplocephalus tutus* followed a similar trajectory as that observed for *Pachycephalosaurus* and *Triceratops*, with the squamosal horns being resorbed and the cranial sculpturing becoming less distinct. UALVP 31 appears to have resorption pits on the squamosal horns, which would support this hypothesis (Fig. 3B). Penkalski and Blows [34] state that none of the referred *Euoplocephalus* specimens, except for AMNH 5266 (herein considered *Anodontosaurus*) represented young juveniles. The ontogenetic stage of a dinosaur is best assessed using histological sections, and no studies have been published on histological sections of ankylosaur long bones for the purpose of determining ontogenetic stage. As such, it is not currently possible to confidently determine the relative ontogenetic stages of ankylosaurs, let alone specimens previously referred to *Euoplocephalus*. Histological sampling and analysis is required in order to test the hypothesis that changes in cranial ornamentation in *Euoplocephalus* are related to ontogeny.

Many of the diagnostic features of *Euoplocephalus tutus* proposed by Coombs [4] and Vickaryous and Russell [16] have broader distributions among *Anodontosaurus*, *Dyoplosaurus*, and *Scolosaurus*. Ciliary osteoderms are also preserved in the holotype of *Dyoplosaurus* (Fig. 6G) and a shallow nasal vestibule, intranarial septum formed by a vertical process of the premaxilla, and medially convergent but anteriorly and posteriorly divergent maxillary tooth rows occur in *Anodontosaurus* and *Scolosaurus*. Premaxillae that are not covered by expanded nasals and that are equal or wider than the width between the most posterior maxillary teeth, slit-like nostrils, and a palate that does not taper anteriorly occur in both *Anodontosaurus* and *Scolosaurus* as well.

If the differences between the skulls of AMNH 5337 and UALVP 31 are not taxonomically significant, and because the pelvis of AMNH 5337 differs from those of *Dyoplosaurus acutosquameus* and *Scolosaurus cutleri* (but is consistent with what is preserved in UALVP 31), then AMNH 5337 is probably referable to *Euoplocephalus tutus*. In turn, the cervical half ring morphology of AMNH 5337 is similar to those of AMNH 5403, AMNH 5404, and AMNH 5405, all of which include skulls. Postcranially, AMNH 5337 and AMNH 5404 are large, robust individuals, whereas AMNH 5406, CMN 210, and UALVP 31 are relatively small, gracile individuals. Compared to other specimens, AMNH 5337 and AMNH 5404 have relatively larger deltopectoral and lateral supracondylar crests of the humeri and have muscle scars that are more prominent (Fig. 11D, E, I, J). The first cervical half rings of AMNH 5337, AMNH 5403, AMNH 5404, and AMNH 5405 are anteroposteriorly longer than those of AMNH 5406, CMN 0210, and UALVP 31 (Fig. 13, Table S9). Penkalski [13] suggested that the cervical half ring of AMNH 5406 was similar in size to other referred *Euoplocephalus tutus* specimens, which is true in terms of the mediolateral width, but not in terms of anteroposterior length.

Larger first cervical half rings are also found in specimens with lower, more rugose, and less distinct primary osteoderms (AMNH 5337, AMNH 5403, AMNH 5404; Fig. 13J–L) compared to specimens with anteroposteriorly shorter cervical half rings (AMNH 5406, CMN 210, UALVP 31; Fig. 13A–H). As in the skulls, perhaps the cervical half ring osteoderms fused with the band but were resorbed during ontogeny. UALVP 31 has resorption pits on the apices of the medial osteoderms on the first cervical half ring (Fig. 13H). Larger cervical half rings with more rugose primary osteoderms that are completely fused to the cervical rings may belong to ontogenetically older individuals. Alternately, more robust individuals referred to *Euoplocephalus tutus* represent a distinct species; however, this seems unlikely given the continuum of morphologies observed in the referred specimens.

AMNH 5403 and AMNH 5405 include tail clubs with the round, semicircular morphology (e.g. Fig. 14H, J–L) that is distinct from the tail club knobs of *Anodontosaurus lambei* (AMNH 5245; Fig. 14A), and *Dyoplosaurus acutosquameus* (ROM 784, UALVP 47273; Fig. 14O, P); *Anodontosaurus* knobs are wider than long and have triangular major osteoderms, and *Dyoplosaurus* knobs are longer than wide. The tail club of TMP 2001.42.9 (Fig. 14M), here referred to *Scolosaurus cutleri*, has a similar round shape to those of AMNH 5403 and AMNH 5405. Because *Euoplocephalus* and *Scolosaurus* appear to overlap stratigraphically, and because their tail club morphology is similar, isolated round tail club knobs from the Dinosaur Park Formation can no longer be referred to *Euoplocephalus tutus*.

Penkalski [13] suggested that ROM 1930 may be referable to *Scolosaurus cutleri*, although he did not formally resurrect that species. In particular, he indicated that radially ribbed, perforate, conical osteoderms only occur in ROM 1930 and NHMUK R5161 ([13]∶270). Later in the same paper, he stated that AMNH 5337 has perforate osteoderms ([13]: 287) and that most referred *Euoplocephalus tutus* osteoderms have some degree of ribbing or fluting ([13]∶289). Penkalski and Blows [34] suggest that ROM 1930 might be referable to *Scolosaurus* (although do not list it as a referred specimen), citing the lack of low-keeled osteoderms found in other *Euoplocephalus* specimens like AMNH 5406, and the presence of conical osteoderms similar to those in NHMUK R5161. ROM 1930 includes a skull (Figs. 3, 4, 5), three dorsal vertebrae, partial sacrum (Fig. 9A, B), caudal vertebrae (Fig. 9M), fragmentary right scapula, right humerus (Fig. 11C), and osteoderms (including *in situ* osteoderms on two blocks of articulated free caudal vertebrae). Field notes by G. F. Sternberg (1914; CMN) indicate that cervical half rings may also have been collected, but these are not yet prepared. The skull lacks postocular osteoderms and the squamosal horns do not have the long, backswept morphology of those from the Two Medicine Formation. If the referral of the Two Medicine ankylosaurid material to *Scolosaurus* is correct, then ROM 1930 is not referable to *Scolosaurus*.

ROM 813 is a remarkable but problematic specimen that preserves keratinous scale impressions as well as the underlying (deep) ossicles and osteoderms [58]. This specimen was referred to *Euoplocephalus tutus* by Penkalski [13]. However, there are few features that allow it to be confidently assigned to Ankylosauridae, let alone to a particular genus or species. The straight shaft of the broken ischium, and the rugose, thin-walled osteoderms suggest that ROM 813 is an ankylosaurid rather than a nodosaurid. ROM 813 has rectangular, keeled osteoderms, unlike those present in NHMUK R5161; and because NHMUK R5161 preserves nearly the entire dorsal integument it is unlikely that ROM 813 is referable to *Scolosaurus cutleri*. ROM 813 is from the lowest levels of the Dinosaur Park Formation and as such is unlikely to be referable to *Anodontosaurus lambei*. It does not have any triangular osteoderms such as those present on the tail of *Dyoplosaurus acutosquameus*, but it is unclear if any of the preserved integument in ROM 813 is from the tail. At present, it is impossible to determine if ROM 813 is referable to *Dyoplosaurus* or *Euoplocephalus.* This unsatisfactory result can only be resolved by finding additional specimens with *in situ* integument.

### Systematic Paleontology

DINOSAURIA Owen, 1842 [78].

ORNITHISCHIA Seeley, 1887 [79].

THYREOPHORA Nopcsa, 1915 [80].

ANKYLOSAURIA Osborn, 1923 [81].

ANKYLOSAURIDAE Brown, 1908 [28].

ANKYLOSAURINAE Brown, 1908 [28].

### *Anodontosaurus Lambei* Sternberg, 1929 [19]

#### Holotype

CMN 8530, skull, lower jaws, caudal vertebra, ischium, pedal phalanx, and osteoderms(including first cervical half ring).

#### Referred Specimens

AMNH 5216 (tail club), AMNH 5223 (skull), AMNH 5245 (caudosacral and caudal vertebra, pelvis, tail club), NHMUK R4947 (skull), ROM 832 (fragmentary skull), TMP 1982.9.3 (two posterior dorsals with coossifed ribs, partial pelvis, right femur, osteoderms including cervical half ring fragments), TMP 1994.168.1 (tail club), TMP 1996.75.01 (partial skull, cervical vertebra, partial first cervical half ring, second cervical half ring), TMP 1997.59.1 (skull), TMP 1997.132.01 (skull, three dorsal vertebrae, ribs, scapula, left humerus, ulna, radius, tibia, first and possibly second cervical half rings), USNM 10753 (tail club).

#### Holotype Locality

“90 feet above Red Deer river, in sec. 3, tp. 21, range 31, W. 4^th^ prin. mer. This locality is about 8 miles southwest of Morrin, Alberta.” ([19]∶28).

#### Distribution

Red Deer River, from Tolman Bridge to Drumheller, Alberta; Dinosaur Provincial Park, Alberta; South Saskatchewan River near Hilda, Alberta.

#### Formations

Horseshoe Canyon Formation; holotype probably from within the Horsethief Member, but referred specimens found throughout the Horsethief, Morrin, and Tolman members. Also present in the upper Dinosaur Park Formation, more than 30 meters above the Oldman-Dinosaur Park contact.

#### Revised Differential Diagnosis

Differs from *Euoplocephalus tutus* and *Scolosaurus cutleri* in having subcircular caputegulae at bases of quadratojugal and squamosal horns (postocular caputegulae), and interstitial osteoderms at bases of primary osteoderms on first cervical half ring; differs from *Euoplocephalus tutus* and *Dyoplosaurus acutosquameus* in having pointed, triangular major osteoderms on tail club knob and in having tail club knob width greater than length; differs from *Dyoplosaurus acutosquameus* in having laterally-directed sacral ribs, and U-shaped pedal unguals; differs from *Scolosaurus cutleri* in having a proportionately shorter postacetabular process of the ilium; differs from *Ankylosaurus magniventris* in having anteriorly-directed nares, and in lacking a continuous keel between the squamosal horn and supraorbitals.

### *Dyoplosaurus Acutosquameus* Parks, 1924 [20]

#### Holotype

ROM 784, fragmentary skull, complete caudal series of vertebrae including tail club, ribs, pelvis, hindlimb including pes, osteoderms *in situ*.

#### Referred Specimens

UALVP 47273 (partial tail club).

#### Holotype Locality

Dinosaur Provincial Park, Quarry Q002, 12U 5622422.480N, 466786.580 E.

#### Distribution

Dinosaur Provincial Park, Alberta.

#### Formation

Lower part of Dinosaur Park Formation.

#### Revised Differential Diagnosis

Differs from *Anodontosaurus lambei*, *Euoplocephalus tutus, and Scolosaurus cutleri* in having anterolaterally-directed sacral ribs, in having triangular unguals in dorsal view, and in having a tail club knob that is longer than wide; differs from *Scolosaurus cutleri* in having a proportionately shorter postacetabular process of the ilium, and in having triangular osteoderms on the lateral sides of the anterior portion of the tail; differs from *Ankylosaurus magniventris* in having anteriorly-directed nares, and in lacking a continuous keel between the squamosal horn and supraorbitals.

### *Euoplocephalus Tutus* Lambe, 1910 [2]

 = *Stereocephalus tutus* Lambe, 1902 [1].

#### Holotype

CMN 210, fragmentary skull roof and partial first cervical half ring.

#### Referred Specimens

AMNH 5337 (skull, left mandible, one cervical vertebra, eleven dorsal vertebrae, humeri, scapulocoracoid, pelvis, osteoderms including first cervical half ring), AMNH 5403 (skull, both mandibles including predentary, four cervicals including axis, scapula, forelimbs, first and second cervical half rings, partial tail club knob), AMNH 5404 (skull, five caudals, ribs, right humerus, ischium, right femur, tibia, fibula, osteoderms, first cervical half ring), AMNH 5405 (skull, right mandible including predentary, handle vertebrae, humerus, ulna, osteoderms, first cervical half ring, tail club knob), AMNH 5406 (three dorsal vertebrae, ribs, scapulae, right humerus, ulna, radius, phalanges, osteoderms including first and second cervical half rings), CMN 842 (first cervical half ring), CMN 8876 (skull), ROM 1930 (skull, three dorsal vertebrae, two sacral vertebrae, twelve free caudals, transitional caudal, fragmentary right scapula, right humerus, osteoderms including *in situ* osteoderms and skin impressions on caudal vertebrae), TMP 1979.14.74 (partial skull), UALVP 31 (skull, right mandible, ribs, sacrum, scapula, humeri, right ilium, right ischium, right femur, tibia, pedal elements, osteoderms including first and second cervical half rings), UALVP 47977 (partial skull roof).

#### Holotype Locality

Dinosaur Provincial Park, exact locality unknown. Collected by L.M. Lambe in 1897 from the east side of the Red Deer River near the mouth of Berry Creek. This refers to the northwestern area of the park, near the old town of Steveville.

#### Distribution

Dinosaur Provincial Park, Alberta; near Manyberries, Alberta.

#### Formation

Dinosaur Park Formation, found primarily in the lower 30 m of the formation.

#### Revised Differential Diagnosis

Differs from *Anodontosaurus lambei* and *Scolosaurus cutleri* in lacking subcircular caputegulae at the bases of the quadratojugal and squamosal horns (postocular caputegulae); differs from *Anodontosaurus lambei* in lacking interstitial osteoderms at the bases of the primary osteoderms of the first cervical half ring, and in having semicircular major osteoderms in dorsal view on the tail club; differs from *Dyoplosaurus acutosquameus* in having laterally-directed sacral ribs; differs from *Scolosaurus cutleri* in having oval to subcircular-based keeled medial and lateral primary half ring osteoderms and in having a proportionately shorter postacetabular process of the ilium; differs from *Ankylosaurus magniventris* in having anteriorly-directed nares, and in lacking a continuous keel between the squamosal horn and supraorbitals.

### *Scolosaurus Cutleri* Nopcsa, 1928 [21]

 = *Oohkotokia horneri* Penkalski, in press [25].

#### Holotype

NHMUK R5161, nearly complete skeleton with *in situ* osteoderms and skin impressions, lacking skull, distal half of tail, right forelimb, and right hindlimb.

#### Referred Specimens

MOR 433 (partial skull, both humeri, free caudal vertebra, and osteoderms), FPDM V-31 (partial skull and partial, reconstructed, mounted skeleton), NSM PV 20381 (skull, dorsal and caudal vertebrae, including damaged handle vertebrae, ribs, both scapulae, both ilia, partial ischia, and both femora, tibiae, and fibulae), TMP 2001.42.19 (skull, partial first cervical half ring, dorsals, sacrals, caudals including complete tail club, left humerus, left scapula, right femur, right and left tibiae, osteoderms), USNM 7943 (partial first cervical half ring).

#### Holotype Locality

Dinosaur Provincial Park, Quarry Q080, 12U, 5,622,321.978 N, 471,365.051 E; there is uncertainty over whether this is the correct quarry or whether it is from several hundred meters farther north.

#### Distribution

Dinosaur Provincial Park, Alberta; northwestern Montana.

#### Formations

Lower part of the Dinosaur Park Formation (or possibly Oldman Formation if mapped quarry position is wrong), and upper part of the Two Medicine Formation.

#### Revised Differential Diagnosis

Differs from *Anodontosaurus lambei* and *Euoplocephalus tutus* in the morphology of the squamosal horns, which are proportionately longer, backswept, and with distinct apices; differs from *Euoplocephalus tutus* in having small circular caputegulae at the bases of the squamosals and quadratojugals; differs from *Anodontosaurus lambei*, *Euoplocephalus tutus*, and *Dyoplosaurus acutosquameus* in having a proportionately longer postacetabular process of the ilium; differs from *Anodontosaurus lambei* and *Euoplocephalus tutus* in having proportionately large circular medial osteoderms with a low central prominences, and compressed, half-moon shaped lateral/distal osteoderms on the cervical half rings; differs from *Dyoplosaurus acutosquameus* in having laterally-directed sacral ribs; differs from *Dyoplosaurus acutosquameus* in having conical, osteoderms with centrally positioned apices on the lateral sides of the anterior portion of the tail; differs from *Anodontosaurus* and *Dyoplosaurus* in having a circular tail club knob in dorsal view, rather than a tail club knob that is wider than long (*Anodontosaurus*) or longer than wide (*Dyoplosaurus*); differs from *Ankylosaurus magniventris* in having anteriorly-directed nares, and in lacking a continuous keel between the squamosal horn and supraorbitals.

### Indeterminate Ankylosauridae

#### Alberta

AMNH 5211 (tail club), AMNH 5266 (juvenile individual with vertebrae, ischium, right hindlimb with pes), CMN 125 (skull roof fragment), CMN 135 (tail club knob), CMN 268 (fragmentary first cervical ring), CMN 349 (tail club), CMN 2251 (partial tail club knob), CMN 2252 (partial tail club knob), CMN 2253 (partial tail club knob), MACN Pv 12554 (tail club), NHMUK R8265 (left quadratojugal horn), NHMUK R36629 (posterior supraorbital), NHMUK R36630 (quadratojugal horn), NHMUK R36631 (squamosal horn), ROM 788 (tail club), ROM 813 (partial skeleton with *in situ* osteoderms, skin impressions), ROM 7761 (tail club knob), TMP 1967.13.2 (tail club knob fragment), TMP 1967.19.4 (left squamosal horn), TMP 1967.20.20 (right quadratojugal horn), TMP 1979.14.164 (partial skull), TMP 1980.8.284 (supraorbital), TMP 1980.16.1685 (fragmentary right mandible), TMP 1983.36.120 (tail club), TMP 1984.121.33 (partial tail club knob), TMP 1985.36.70 (free caudal vertebra), TMP 1985.36.330 (highly fragmentary skull in numerous pieces), TMP 1988.106.5 (left supraorbital), TMP 1991.36.321 (fragmentary first cervical ring), TMP 1991.36.743 (portion of frontonasal region), TMP 1992.36.334 (free caudal vertebra), TMP 1992.36.421 (right mandible), TMP 1993.36.79 (left squamosal), TMP 1993.36.421 (tail club), TMP 1998.83.1 (skull, cervical half ring: indeterminate because unprepared as of 2012), TMP 1993.66.13 (quadratojugal horn), TMP 1996.12.15 (portion of supraorbital region), TMP 1997.36.313 (right mandible), TMP 1998.93.55 (free caudal vertebra), TMP 1998.93.65 (free caudal vertebra), TMP 2000.57.3 (phalanges, tail club), TMP 2000.57.30 (portion of lacrimal/frontonasal region), TMP 2003.12.166 (fragmentary second cervical ring), TMP 2003.12.169 (first cervical ring distal osteoderm), TMP 2003.12.311 (skull, cervical half ring: indeterminate because unprepared as of 2012), TMP 2004.98.06 (mandible), TMP 2005.09.75 (free caudal), TMP 2005.12.43 (free caudal vertebra), TMP 2005.49.178 (portion of frontonasal region), TMP 2007.020.0063 (small quadratojugal horn), TMP 2007.20.80 (free caudal vertebra), TMP 2007.12.52 (second cervical half ring), TMP 2007.20.100 (free caudal vertebra), TMP 2012.005.2 (portion of lacrimal/frontonasal region), UALVP 16247 (tail club), UALVP 45931 (partial first and second cervical half rings), UALVP 47273 (tail club), UALVP 49314 (anterior supraorbital), UALVP 52875 (partial tail club knob), UALVP 54685 (posterior supraorbital). Additionally, many isolated osteoderms and teeth from the Dinosaur Park Formation are in the TMP and UALVP collections.

#### Montana

AMNH 5470 (partial sacrum), AMNH 20870 (handle vertebrae), MOR 363 (braincase, both quadratojugal horns, and skull roof fragments), USNM 16747 (handle vertebrae).

### Phylogenetic Relationships Of Campanian-Maastrichtian Ankylosaurids From Alberta And Montana

**Figure 16:**
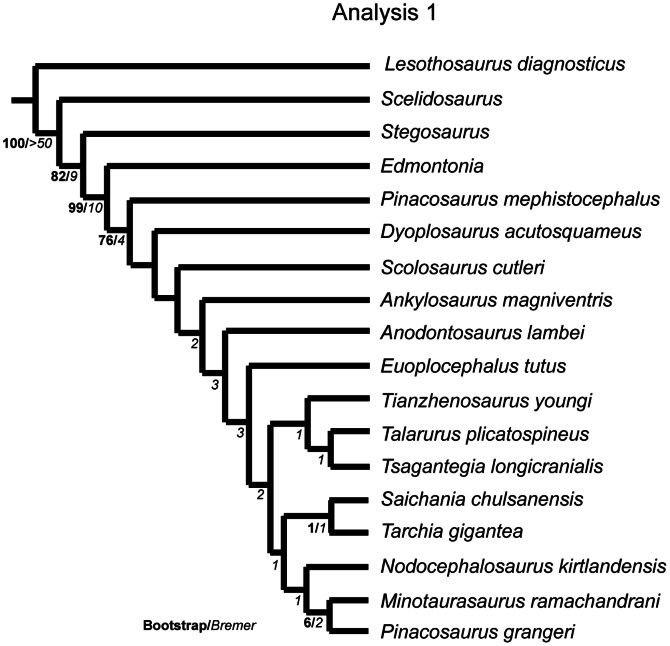
Results of phylogenetic analysis 1, retaining character state codings from Thompson et al. [32]. Strict consensus and 50% majority rule trees shown, with frequencies, bootstrap supports, and Bremer supports.

The analysis retaining the character codings from Thompson et al. [32], Matrix 1, produced two most parsimonious trees, with the best TBR score of 276 reached 150 times out of 192 (Fig. 16). The strict consensus tree has a consistency index (CI) of 0.62, and a retention index (RI) of 0.67. The Albertan ankylosaurids *Ankylosaurus*, *Anodontosaurus*, *Dyoplosaurus*, *Euoplocephalus*, and *Scolosaurus* did not form a clade, but instead formed a series of nested taxa leading towards a clade of Asian ankylosaurids (plus the North American *Nodocephalosaurus*). *Pinacosaurus grangeri* Gilmore, 1933 [82] was more closely related to *Minotaurasaurus* than to *Pinacosaurus mephistocephalus*. Bootstrap and Bremer supports were low for all ankylosaurid interrelationships.

**Figure 17:**
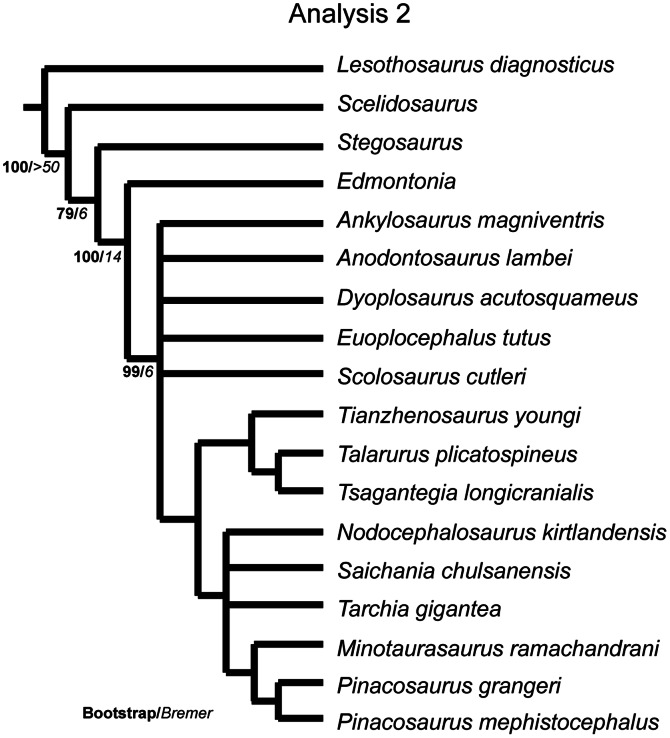
Results of phylogenetic analysis 2, modifying character state codings from Thompson et al. [32].

The analysis with updated codings (Matrix 2) produced six most parsimonious trees of length 254, with the best score reached one time out of eleven (Fig. 17). The strict consensus tree had a CI of 0.63 and a RI of 0.66. Again, bootstrap and Bremer supports for ankylosaurid interrelationships were low. The Albertan ankylosaurids form a polytomy that is the sister group to a clade containing Asian ankylosaurids and *Nodocephalosaurus*. *Pinacosaurus grangeri* and *Pinacosaurus mephistocephalus* were recovered as sister taxa.

**Figure 18:**
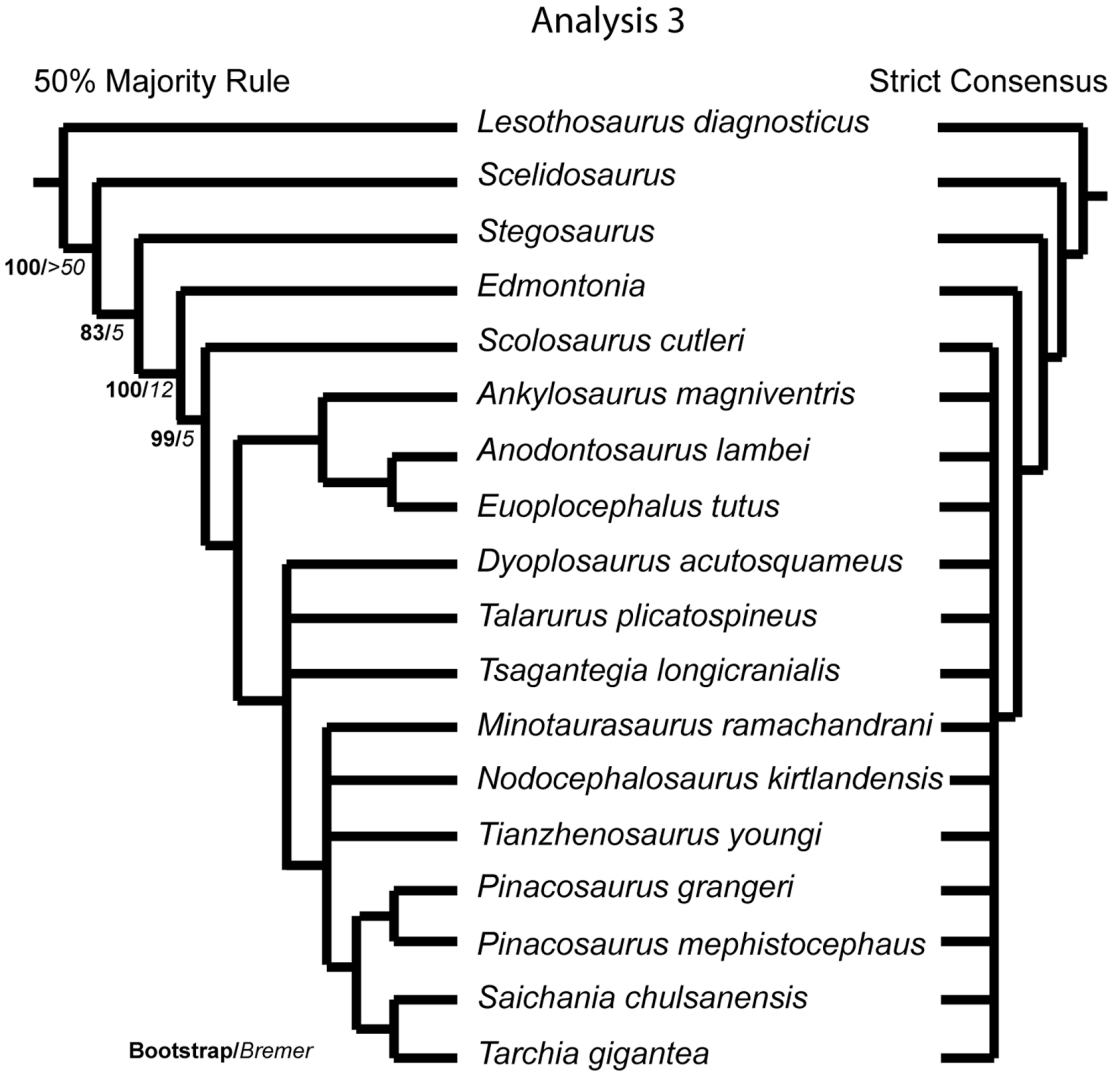
Results of phylogenetic analysis 3, with new characters added to modified character state codings from analysis 2.

The final analysis incorporating new characters identified in this analysis (Matrix 3) resulted in 50 most parsimonious trees with the best TBR score of 269 reached five out of ten times (Fig. 18), with a a CI of 0.65 and a RI of 0.69. Ankylosaurid interrelationships were completely unresolved in the strict consensus tree. A reduced consensus tree also had a completely unresolved Ankylosauridae. Matrix 3 was analyzed in TAXEQ [53] to determine the amount of missing data, and to search for taxonomic equivalents. *Euoplocephalus*, *Minotaurasaurus*, and *Pinacosaurus grangeri* had the least amount of missing data (each under 10%), and *Dyoplosaurus* and *Nodocephalosaurus* had the most missing data (each over 75%); overall, 36% of the character matrix was missing data. Six taxa were found to have potential taxonomic equivalents (*Dyoplosaurus*, *Minotaurasaurus*, *Nodocephalosaurus*, *Tarchia*, *Tianzhenosaurus*, and *Talarurus plicatospineus* Maleev, 1952 [83]), but in all cases the equivalency was asymmetric, and so no taxa could be safely removed from the analysis.

In the 50% majority rule tree, *Ankylosaurus*, *Anodontosaurus*, and *Euoplocephalus* formed a clade in 56% of all trees. *Pinacosaurus* was monophyletic in 72% of all trees. *Scolosaurus* was recovered as the most basal ankylosaurid, but *Dyoplosaurus* was recovered in a clade containing Asian ankylosaurids (plus *Nodocephalosaurus*). *Scolosaurus* has a relatively long postacetabular process (character state 142-0), a feature also present in nodosaurid ankylosaurs and basal thyreophorans, which may contribute to its relatively basal placement.

In all three analyses, the Asian ankylosaurids (plus *Nodocephalosaurus*) formed a monophyletic group in the strict consensus (Analyses 1 and 2) or majority-rule (Analysis 3) trees, but the North American ankylosaurids only partly formed a monophyletic group in Analysis 3. This indicates that, at present, it is best not to consider previous synonyms of *Euoplocephalus* as species of *Euoplocephalus*, but to treat them as distinct genera. The changing topology within the Ankylosauridae across these three analyses highlights the need for careful choice of characters and character codings and the identification of additional new characters.

### Biogeographic And Biostratigraphic Implications

**Figure 19:**
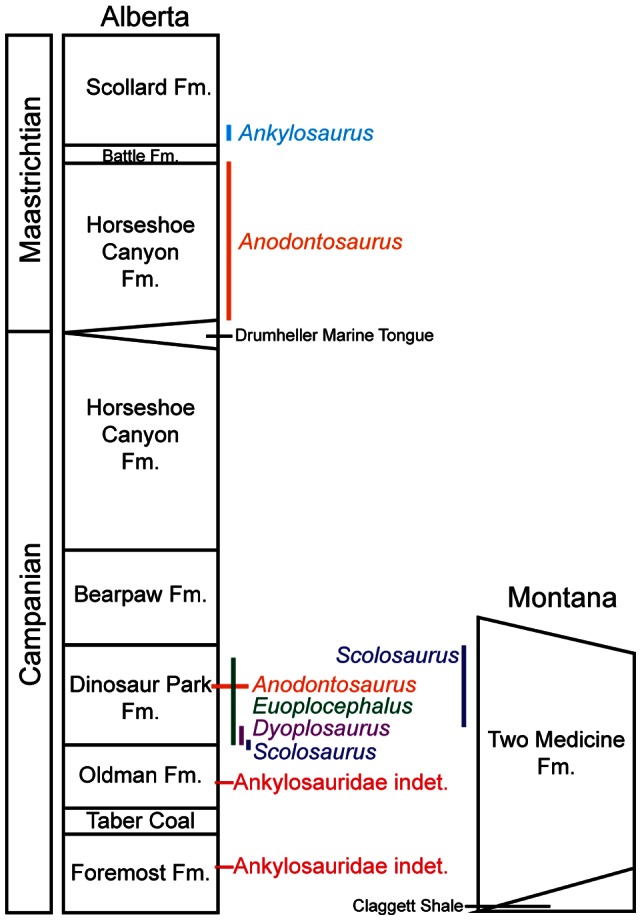
Stratigraphic distribution of Campanian-Maastrichtian ankylosaurid species in Alberta and northwest Montana. Indeterminate ankylosaurid material is known from the Foremost and Oldman formations in southern Alberta. The holotype of *Scolosaurus cutleri* may be from the Oldman Formation or the lower Dinosaur Park Formation; ankylosaurid specimens from the Upper Two Medicine Formation of Montana are referred to *Scolosaurus*. *Dyoplosaurus acutosquameus* is known from Megaherbivore Assemblage Zone 1 in the Dinosaur Park Formation. *Euoplocephalus tutus* has been identified from both Megaherbivore Assemblage Zone 1 and Megaherbivore Assemblage Zone 2 of the Dinosaur Park Formation, but is more common in Zone 1. *Anodontosaurus lambei* is rare in Megaherbivore Assemblage Zone 2 of the Dinosaur Park Formation, with most specimens identified from the Horsethief, Morrin, and Tolman members of the Horseshoe Canyon Formation. In Alberta, *Ankylosaurus magniventris* was present in the Scollard Formation.

The results of this study indicate that ankylosaurid diversity in the Late Cretaceous of Alberta was higher than previously recognized (Fig. 19). Within the Dinosaur Park Formation, there are at least three ankylosaurid species: *Dyoplosaurus acutosquameus*, *Euoplocephalus tutus*, and *Scolosaurus cutleri*. A recent analysis of the biostratigraphy of megaherbivorous dinosaurs in the Dinosaur Park Formation by Mallon et al. [18] found two main assemblage zones: Megaherbivore Assemblage Zone 1 (MAZ-1), from 0 to 28 meters (mostly corresponding to the *Centrosaurus*-*Corythosaurus* faunal zone sensu [36]), and MAZ-2, from 29 to 52 meters (mostly corresponding to the *Styracosaurus* – *Lambeosaurus* faunal zone sensu [36]). MAZ-2 may also extend into the Lethbridge Coal Zone, in the uppermost part of the Dinosaur Park Formation [18], previously considered the pachyrhinosaur-*Lambeosaurus magnicristatus* faunal zone by Ryan and Evans [36]. *Scolosaurus* is currently represented by only a single specimen from either the Oldman Formation or the lower 10 m of the Dinosaur Park Formation (Fig. 1), in MAZ-1a [18]. Even with an additional specimen referred to *Dyoplosaurus*, this taxon is still only found in MAZ-1a as well. *Euoplocephalus* appears to primarily occur in MAZ-1, but two significant specimens–ROM 1930 and TMP 1997.132.1–have been recovered from the upper 30 meters of the formation. At present, ROM 1930 is best referred to *Euoplocephalus*. However, TMP 1997.132.1 shares several features with *Anodontosaurus lambei* from the Horseshoe Canyon Formation, and is here referred to that species. It is unusual for any Albertan dinosaur genus to be present in both the Dinosaur Park and Horseshoe Canyon formations, although cf. *Anchiceratops* and a *Pachyrhinosaurus*-like ceratopsid, both otherwise known only from the Horseshoe Canyon Formation, have been reported from the uppermost Dinosaur Park Formation [18],[65],[84]). At present there are no morphological features that can distinguish the upper Dinosaur Park Formation ankylosaurid from *Anodontosaurus lambei*. Future discoveries may yet provide evidence that the upper Dinosaur Park Formation ankylosaurid warrants taxonomic separation from *Anodontosaurus lambei*. Regardless, there appears to be little stratigraphic overlap between *Anodontosaurus lambei* and the lower Dinosaur Park Formation ankylosaurids *Dyoplosaurus acutosquameus*, *Euoplocephalus tutus*, and *Scolosaurus cutleri*.

In contrast to the high diversity in the lower Dinosaur Park Formation, ankylosaurid specimens in the Horseshoe Canyon Formation are referable only to *Anodontosaurus lambei* at present. *Anodontosaurus lambei* was present throughout the upper part of the formation (Fig. 19). Mallon et al. [66], in an evaluation of variation within the Horseshoe Canyon Formation ceratopsid *Anchiceratops*, noted that this genus had a long stratigraphic range relative to other Albertan ceratopsids. *Anodontosaurus lambei* appears to have had a similarly long stratigraphic range.

The referral of ankylosaurid specimens from the Two Medicine Formation of Montana to *Scolosaurus cutleri*, previously known from only a single specimen from the Dinosaur Park Formation of Alberta, extends the geographic range of this taxon. For the Montanan specimens that had locality information, all were collected from the upper part of the Two Medicine Formation (Fig. 19). The uppermost part of the Two Medicine Formation (10 m below the top of the formation) was dated at 74 Ma [85], whereas the top of the Dinosaur Park Formation was dated at 74.9 Ma [47], and the top of the Oldman Formation within Dinosaur Provincial Park was dated at 76.5 Ma [47]; MOR 433 most likely occurred at a slightly younger time than most of the ankylosaurids from Dinosaur Provincial Park. Although there is uncertainty regarding the stratigraphic position of the holotype of *Scolosaurus*, it probably originated from at least the lowest part of the Dinosaur Park Formation, and it is possible it originated from the underlying Oldman Formation. This might suggest that the referral of specimens from the Two Medicine Formation (*Oohkotokia*) to *Scolosaurus* is incorrect. However, it is possible that the occurrence of *Scolosaurus* in the Two Medicine Formation and (potentially) the Oldman Formation is environmentally and ecologically related: the Oldman Formation represents the maximum regression of the Western Interior Seaway during the Campanian [47], and therefore a comparatively drier, more “upland” environment compared to the Dinosaur Park Formation. Although deposited during a transgressive phase, the Upper Two Medicine Formation represents a relatively dry environment, compared to the laterally equivalent Judith River Formation and the Dinosaur Park Formation [68]. Cranial material associated with a *Scolosaurus* half ring from the Dinosaur Park Formation is needed to confirm the referral of the Two Medicine ankylosaurid material to *Scolosaurus* rather than *Oohkotokia*. Until then, *Oohkotokia* possesses no unique characters that separate it from *Scolosaurus*.

In Alberta, no Judithian ankylosaurid fossils have been recovered north of Dry Island Buffalo Jump Provincial Park, although ankylosaurid teeth have been collected from the Kleskun Hills locality near Grande Prairie [86]. Although the teeth are the right size to be ankylosaurid teeth, the two teeth that were recovered are weathered and may represent teeth of juvenile nodosaurids. Nodosaurid fossils have been collected from as far north as the Matanuska Formation of Alaska [87], but currently ankylosaurids appear to be restricted to more southern parts of Laramidia during the Late Cretaceous.

*Nodocephalosaurus* appears to have been related to the Asian ankylosaurids *Saichania* and *Tarchia*, a relationship first noted by Sullivan [50]. This is unusual, given that *Nodocephalosaurus* is currently known from the Campanian of New Mexico (southern Laramidia), and no northern Laramidian ankylosaurids have recently been hypothesized to have been closely related to any Asian species. It seems unusual that Asian ankylosaurid dinosaurs migrated into North America during the Late Cretaceous without leaving any close relatives in Alaska, Alberta, Montana, or Utah, through what is presumed to be the most likely dispersal route from Asia to New Mexico. The phylogenetic analysis by Thompson et al. [32], as well as Analysis 3 in this paper, recover the Albertan species *Dyoplosaurus acutosquameus* as having affinities with Asian ankylosaurids. Again, it is important to note that the position of *Dyoplosaurus acutosquameus* appears to be quite labile. There is a great deal of missing data in the character matrix for this taxon, and so a close relationship between *Dyoplosaurus acutosquameus* and Asian ankylosaurids should be regarded as tentative at best. However, if further study confirms this relationship, this could support the hypothesis of a dispersal of Asian ankylosaurids into North America during the Late Cretaceous.

### Conclusions

Specimens that were once referred to a single genus, *Euoplocephalus*, are now shown to represent at least four distinct taxa, greatly increasing the diversity of Late Cretaceous North American ankylosaurids. Within Alberta, *Dyoplosaurus acutosquameus*, *Euoplocephalus tutus*, and *Scolosaurus cutleri* were restricted to the lower part of the Dinosaur Park Formation (although *Scolosaurus* may have occurred in the top of the Oldman Formation), and *Anodontosaurus lambei* was present in the upper part of the Dinosaur Park Formation and in the Horseshoe Canyon formation. *Oohkotokia horneri*, from the Two Medicine Formation of Montana, is morphologically indistinct from *Scolosaurus cutleri*. *Dyoplosaurus acutosquameus* has limited cranial material and is represented by only two specimens. In contrast, *Anodontosaurus lambei*, *Euoplocephalus tutus,* and *Scolosaurus cutleri* are known from numerous referred specimens, including both skulls and postcrania. The skeleton of *Anodontosaurus lambei* is not as completely known as that of *Euoplocephalus tutus*, for which nearly the entire skeleton is represented across numerous referred specimens. Although *Euoplocephalus tutus* still includes the most referred material, there is no specimen that includes *in situ* osteoderms, and so the arrangement of osteoderms in *Euoplocephalus tutus* is not known.

The recognition of several species within *Euoplocephalus tutus* sensu lato indicates that *Euoplocephalus tutus* sensu stricto was not as intraspecifically variable as previously suspected. Although cranial ornamentation can be variable, aspects of ankylosaurid cranial ornamentation are taxonomically informative, such as the overall shapes of the squamosal horns, the presence or absence of postocular caputegulae at the bases of the squamosal and quadratojugal horns, the morphology of the first cervical half ring, and the shape and proportions of the tail club knob. The morphology of the pelvis also appears to be taxonomically informative. Conversely, certain aspects of the cranial and postcranial skeleton, such as squamosal horn size and bluntness, cranial caputegulum distinctness, cervical half ring anteroposterior length, and robustness of limb elements (such as the size of the deltopectoral crest of the humerus) are more likely a result of ontogenetic variation. This information can be used to better interpret taxonomic versus intraspecific variation among other ankylosaurid taxa.

## Supporting Information

Figure S1 **CMN 0210, holotype of** ***Euoplocephalus tutus*** **, skull in dorsal and left lateral views with interpretive dorsal view diagram.**
(TIF)

Figure S2 **UALVP 31, referred** ***Euoplocephalus tutus*** **skull in dorsal and right lateral views with interpretive dorsal view diagram.**
(TIF)

Figure S3 **ROM 784, holotype of** ***Dyoplosaurus acutosquameus*** **, skull in dorsal view with interpretive diagram.**
(TIF)

Figure S4 **CMN 8530, holotype of** ***Anodontosaurus lambei*** **, skull in dorsal and left lateral views with interpretive dorsal view diagram.**
(TIF)

Figure S5 **USNM 11892, referred** ***Scolosaurus cutleri*** **skull in dorsal and right lateral views.**
(TIF)

Figure S6 **MOR 433, holotype of** ***Oohkotokia horneri*** **( =** ***Scolosaurus cutleri*** **), in dorsal and right lateral views.**
(TIF)

Figure S7 **TMP 1991.127.1, referred** ***Euoplocephalus tutus*** **skull in dorsal and left lateral views, with interpretive dorsal view diagram.**
(TIF)

Figure S8 **TMP 1997.132.1, referred** ***Anodontosaurus lambei*** **skull in dorsal and left lateral views, with interpretive dorsal view diagram.**
(TIF)

Figure S9 **TMP 2001.42.9, referred** ***Scolosaurus cutleri*** **skull in dorsal and right lateral views.**
(TIF)

Figure S10 **AMNH 5337, referred** ***Euoplocephalus tutus*** **skull in dorsal and right lateral views, with interpretive dorsal view diagram.**
(TIF)

Figure S11 **AMNH 5403, referred** ***Euoplocephalus tutus*** **skull in dorsal and right lateral views. This specimen was sectioned transversely across the rostrum.**
(TIF)

Figure S12 **ROM 832, referred** ***Anodontosaurus lambei*** **skull in dorsal and left lateral views, with interpretive dorsal view diagram.**
(TIF)

Figure S13 **TMP 1997.59.1, referred** ***Anodontosaurus lambei*** **skull in dorsal and left lateral views, with interpretive dorsal view diagram.**
(TIF)

Figure S14 **AMNH 5405, referred** ***Euoplocephalus tutus*** **skull in dorsal and left lateral views, with interpretive dorsal view diagram.**
(TIF)

Figure S15 **ROM1930, referred** ***Euoplocephalus tutus*** **skull in dorsal and right lateral views, with interpretive dorsal view diagram.**
(TIF)

Figure S16 **NHMUK R4947, referred** ***Anodontosaurus lambei*** **skull in dorsal and left lateral views, with interpretive dorsal view diagram.**
(TIF)

Figure S17 **AMNH 5238, referred** ***Anodontosaurus lambei*** **skull in dorsal and right lateral views, with interpretive dorsal view diagram.**
(TIF)

Figure S18 **AMNH 5223, referred** ***Anodontosaurus lambei*** **skull in dorsal and right lateral views.**
(TIF)

Figure S19 **TMP 1996.75.1, referred** ***Anodontosaurus lambei*** **skull in dorsal and left lateral views.**
(TIF)

Table S1 **Locality information for specimens referred to Anodontosaurus lambei, Dyoplosaurus acutosquameus, Euoplocephalus tutus, and Scolosaurus cutleri.**
(XLSX)

Table S2 **General dimensions of skulls examined for this study, in millimeters.**
(XLSX)

Table S3 **Detailed measurements of skulls examined for this study, in millimeters.**
(XLSX)

Table S4 **Measurements of the cervical and dorsal vertebrae, in millimeters.**
(XLSX)

Table S5 **Measurements of the sacral and free caudal vertebrae, in millimeters.**
(XLSX)

Table S6 **Measurements of the handle vertebrae of the tail club, in millimeters.**
(XLSX)

Table S7 **Measurements of the pectoral girdle and forelimb elements, in millimeters.**
(XLSX)

Table S8 **Measurements of the pelvic girdle and hindlimb elements, in millimeters.**
(XLSX)

Table S9 **Measurements of the cervical half rings, in millimeters.**
(XLSX)

Table S10 **Measurements of the tail club knob, in millimeters.**
(XLSX)

Locality Data S1 **Specimens from Alberta plotted geographically using Google Earth (.kmz file).** Specimens have been located as accurately as possible given current locality information provided in Table S1. Locality accuracy is colour coded as follows: red – only a general region is known, e.g. Little Sandhill Creek; yellow – based entirely on field notes, which usually give distances in miles from a particular landmark; blue – Township and Range coordinates; green – GPS coordinates. NHMUK R5161 is marked in purple because it is uncertain whether or not this is the correct quarry for this specimen. Localities within Dinosaur Provincial Park were also cross-checked against an overlay of the Steveville Map 969A [44]. Note: Fossils in Alberta are protected under the Historical Resources Act, which prohibits the excavation of fossils without a permit, and prohibits surface collection within provincial and national parks and protected areas. For more information on the Historical Resources Act, visit the Alberta Queen’s Printer website, http://www.qp.alberta.ca/index.cfm. (KMZ)

Character Statements S1 **List of characters used in the phylogenetic analysis, and changes to previously published character codings.**
(DOCX)

Character Matrix S1 **Phylogenetic data matrix 1, original codings plus** ***Anodontosaurus lambei*** **and** ***Scolosaurus cutleri*** **.**
(NEX)

Character Matrix S2 **Phylogenetic data matrix 2, updated codings.**
(NEX)

Character Matrix S3 **Phylogenetic data matrix 3, updated codings with new characters.**
(NEX)
