# Supplementary figures and images for: Euoplocephalus tutus and the Diversity of Ankylosaurid Dinosaurs in the Late Cretaceous of Alberta, Canada, and Montana, USA
Source: PLoS One. 2013 May 8;8(5):e62421. doi: 10.1371/journal.pone.0062421 (PMC3648582; doi:10.1371/journal.pone.0062421)

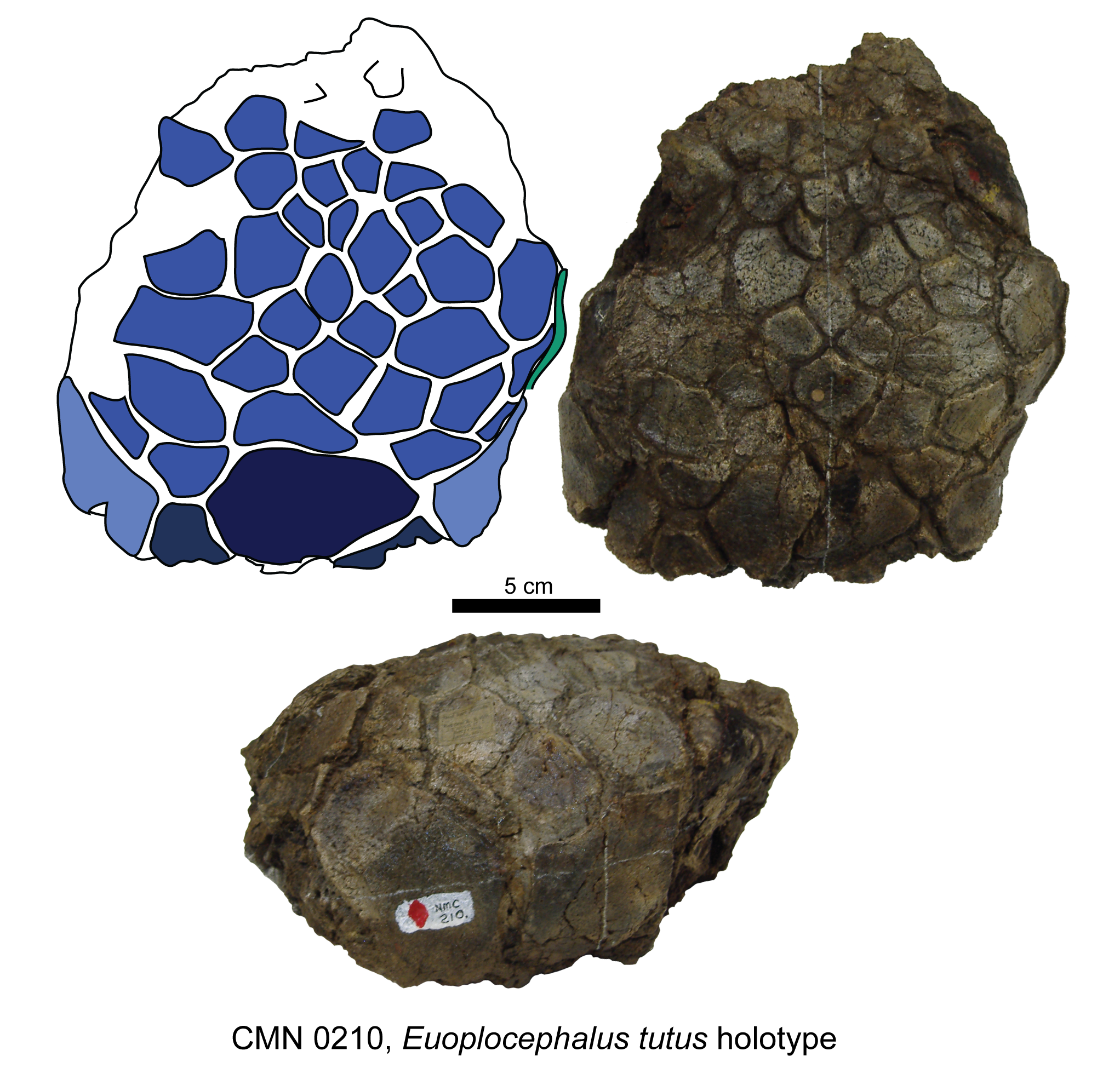

Supplement: Figure S1 — (TIF) [file pone.0062421.s001.tif]

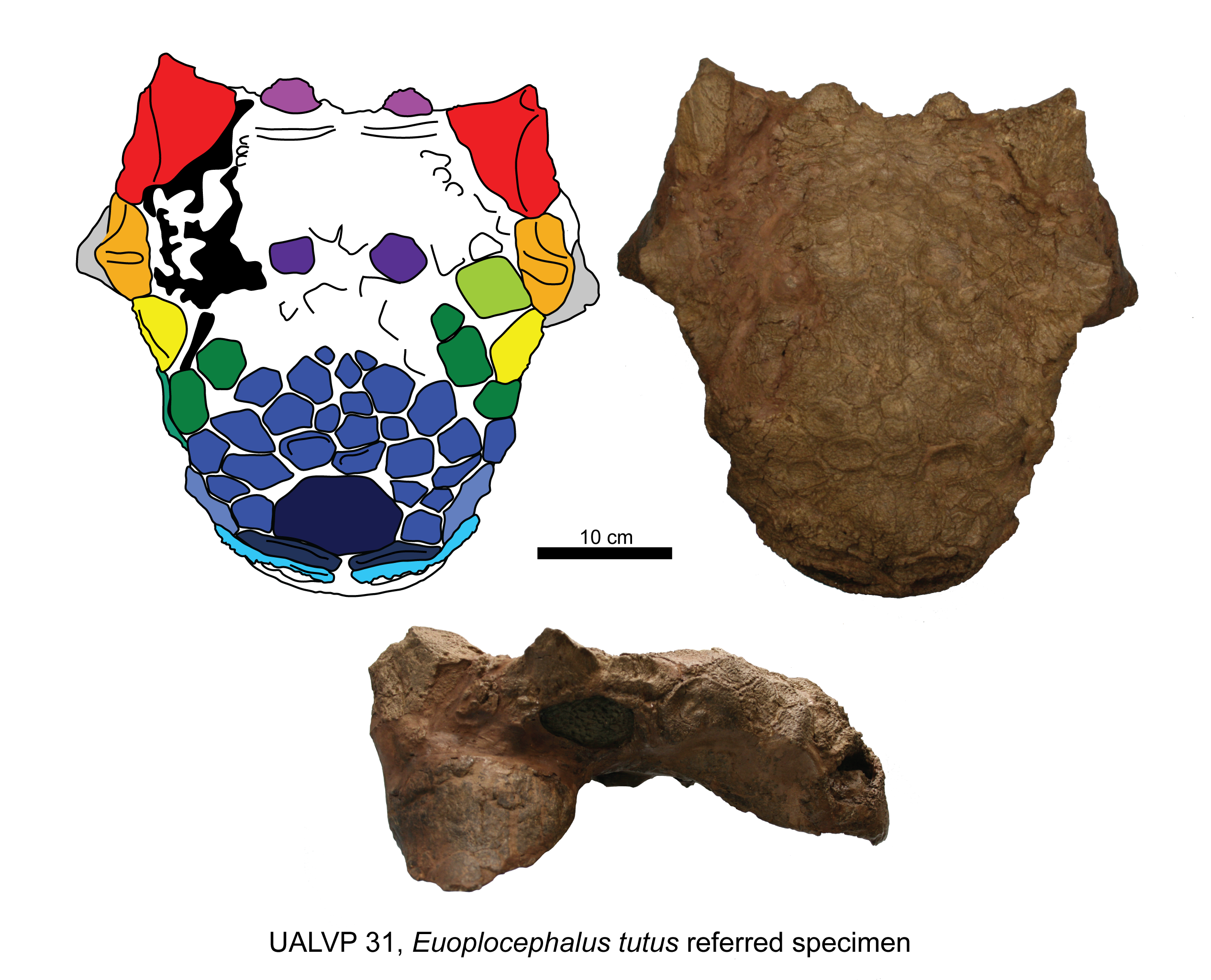

Supplement: Figure S2 — (TIF) [file pone.0062421.s002.tif]

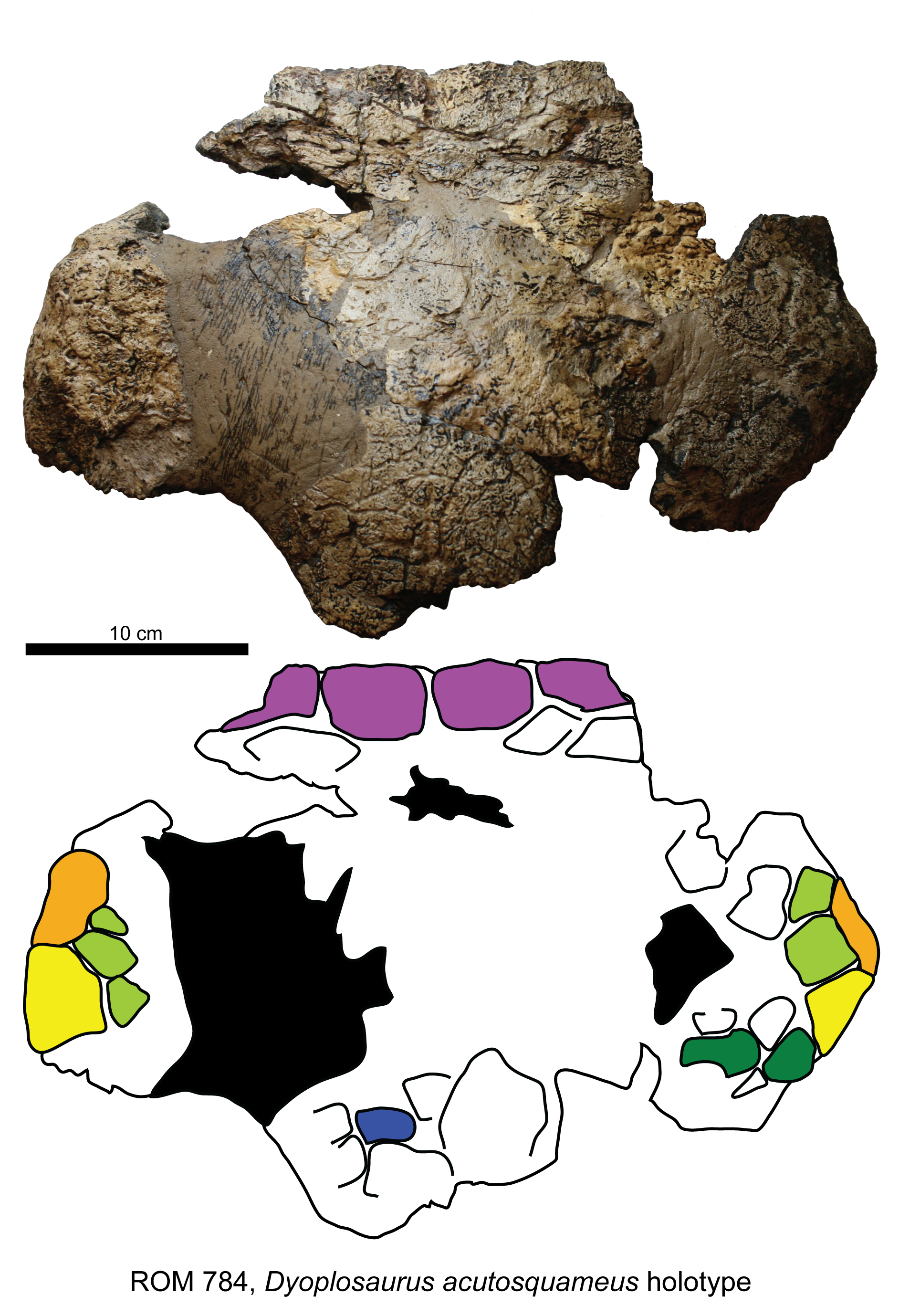

Supplement: Figure S3 — (TIF) [file pone.0062421.s003.tif]

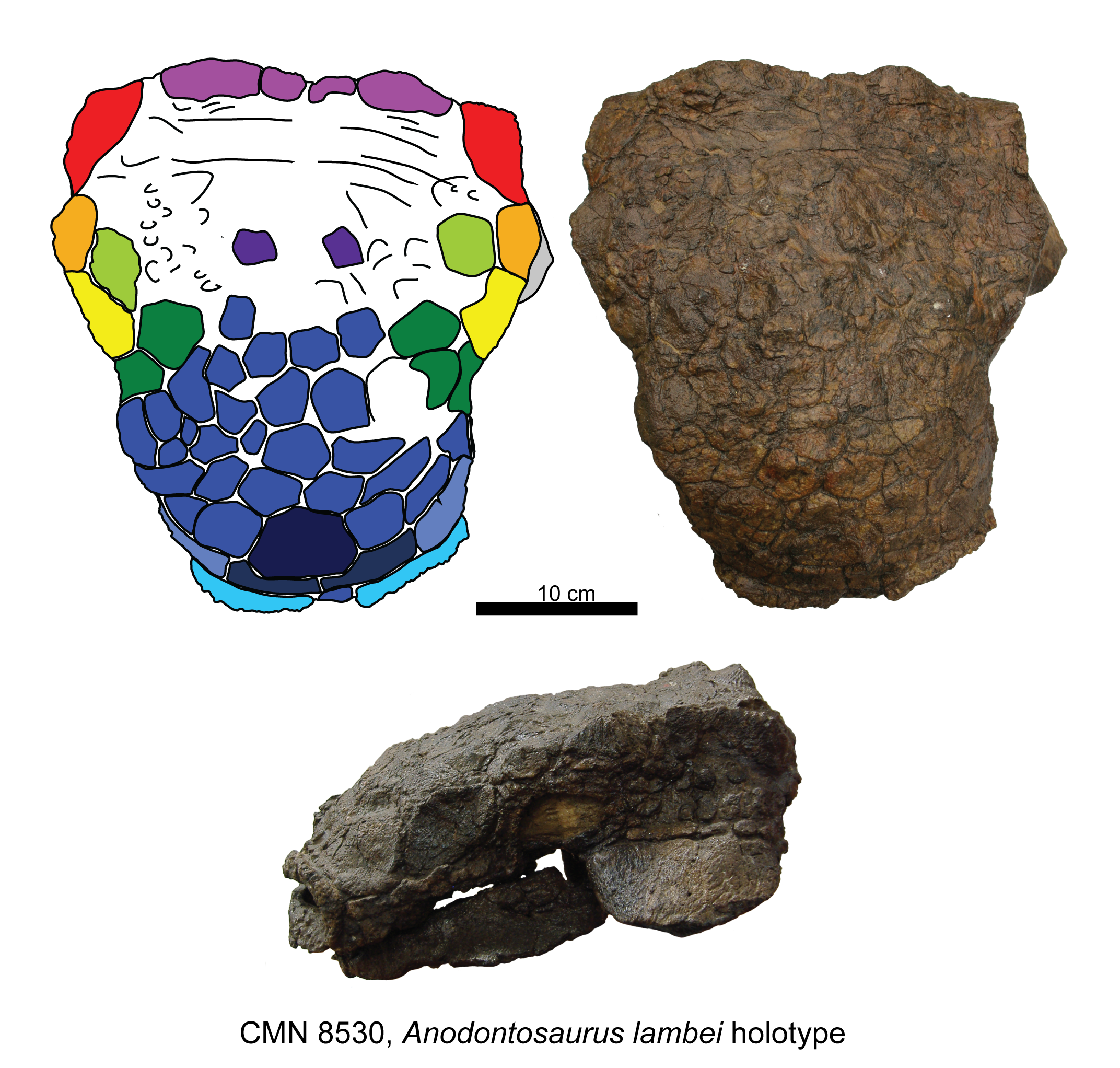

Supplement: Figure S4 — (TIF) [file pone.0062421.s004.tif]

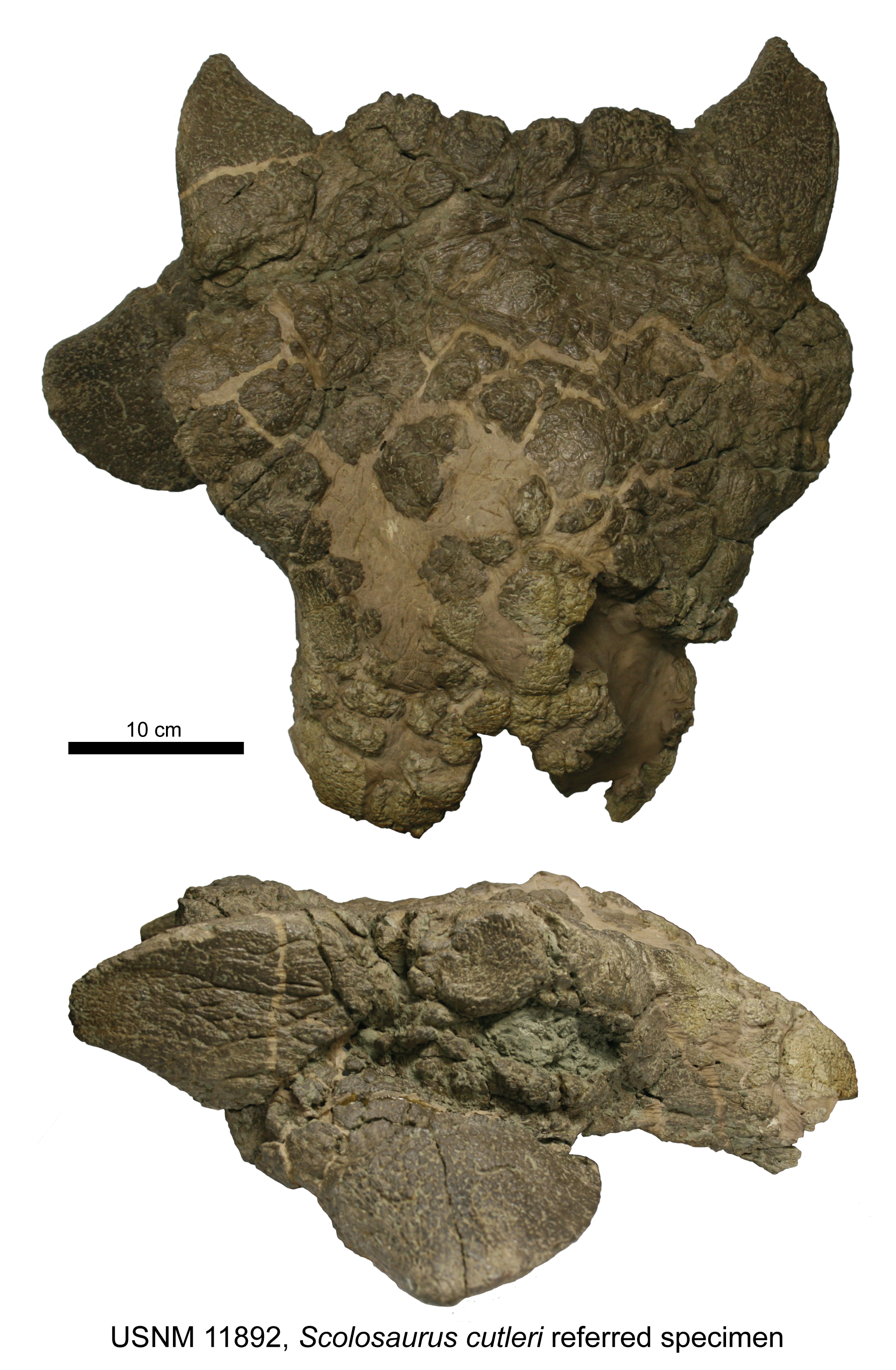

Supplement: Figure S5 — (TIF) [file pone.0062421.s005.tif]

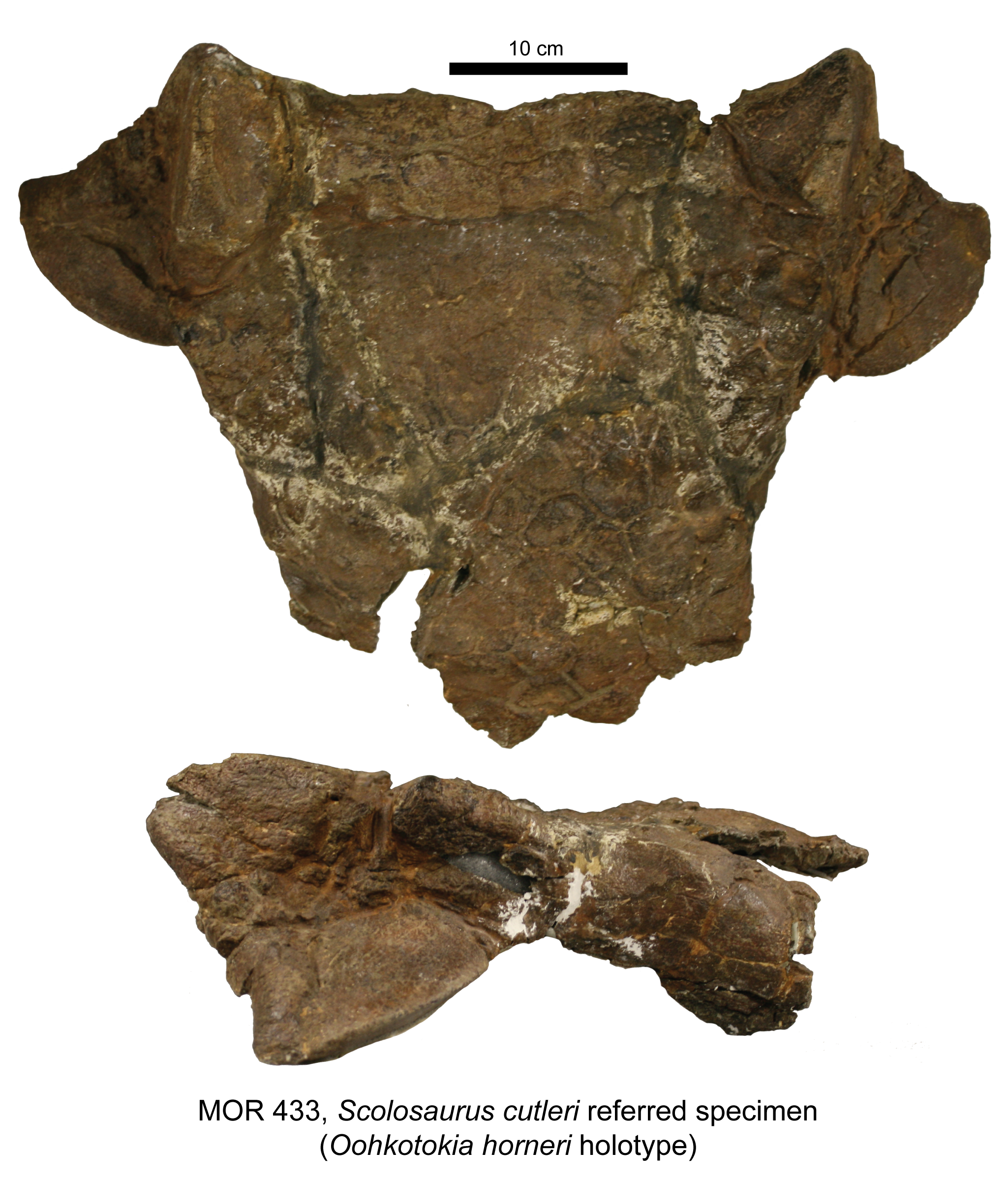

Supplement: Figure S6 — (TIF) [file pone.0062421.s006.tif]

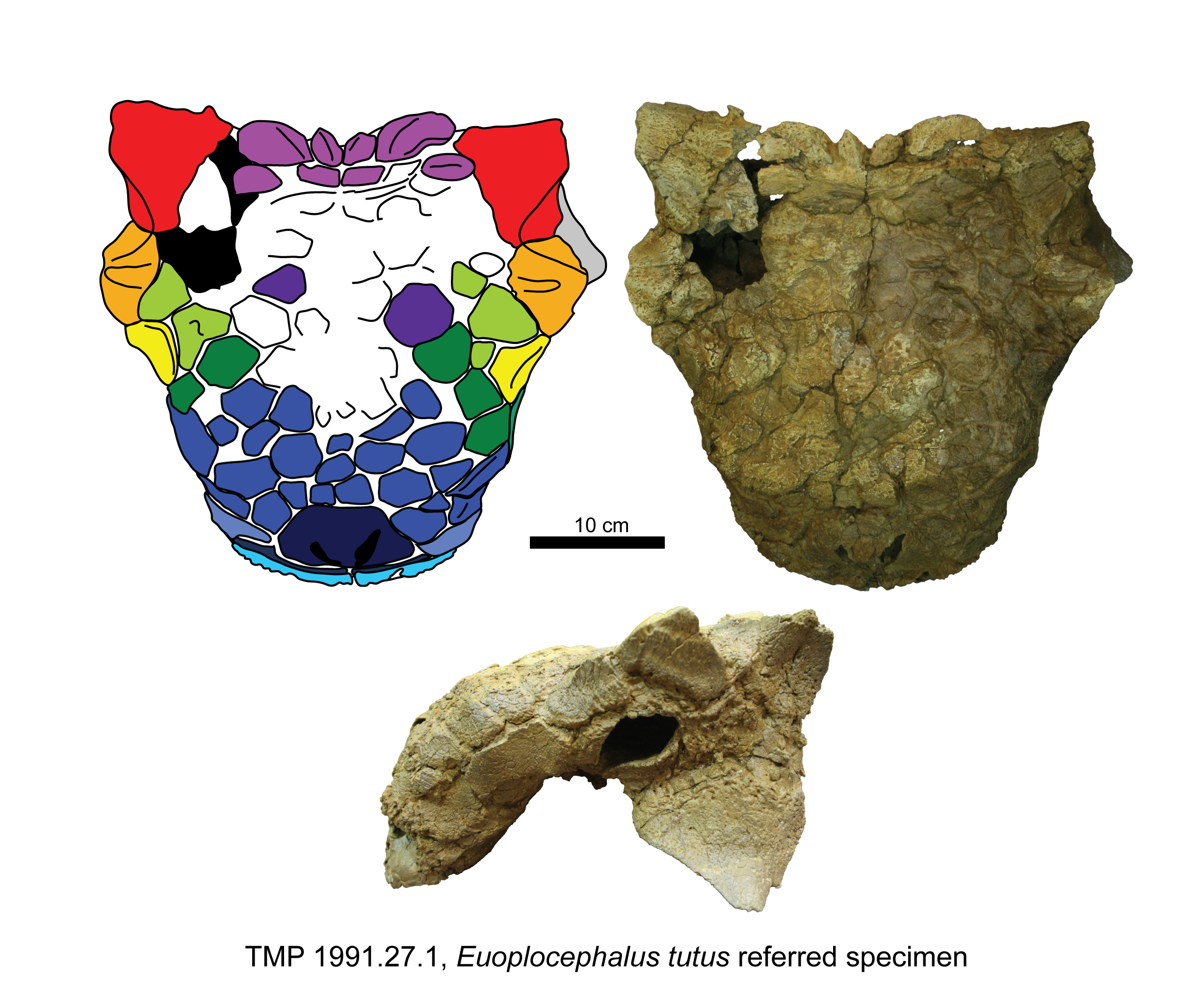

Supplement: Figure S7 — (TIF) [file pone.0062421.s007.tif]

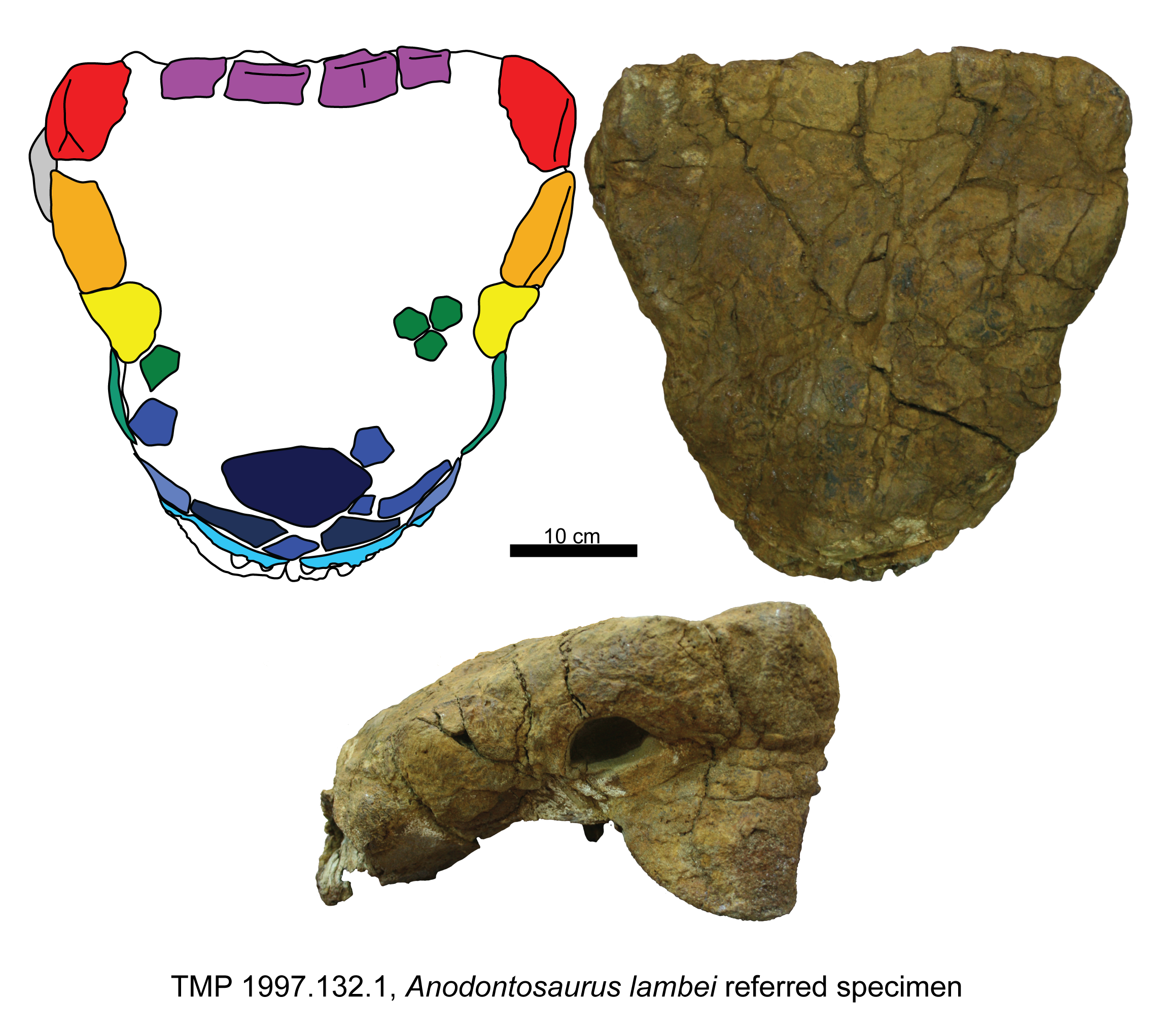

Supplement: Figure S8 — (TIF) [file pone.0062421.s008.tif]

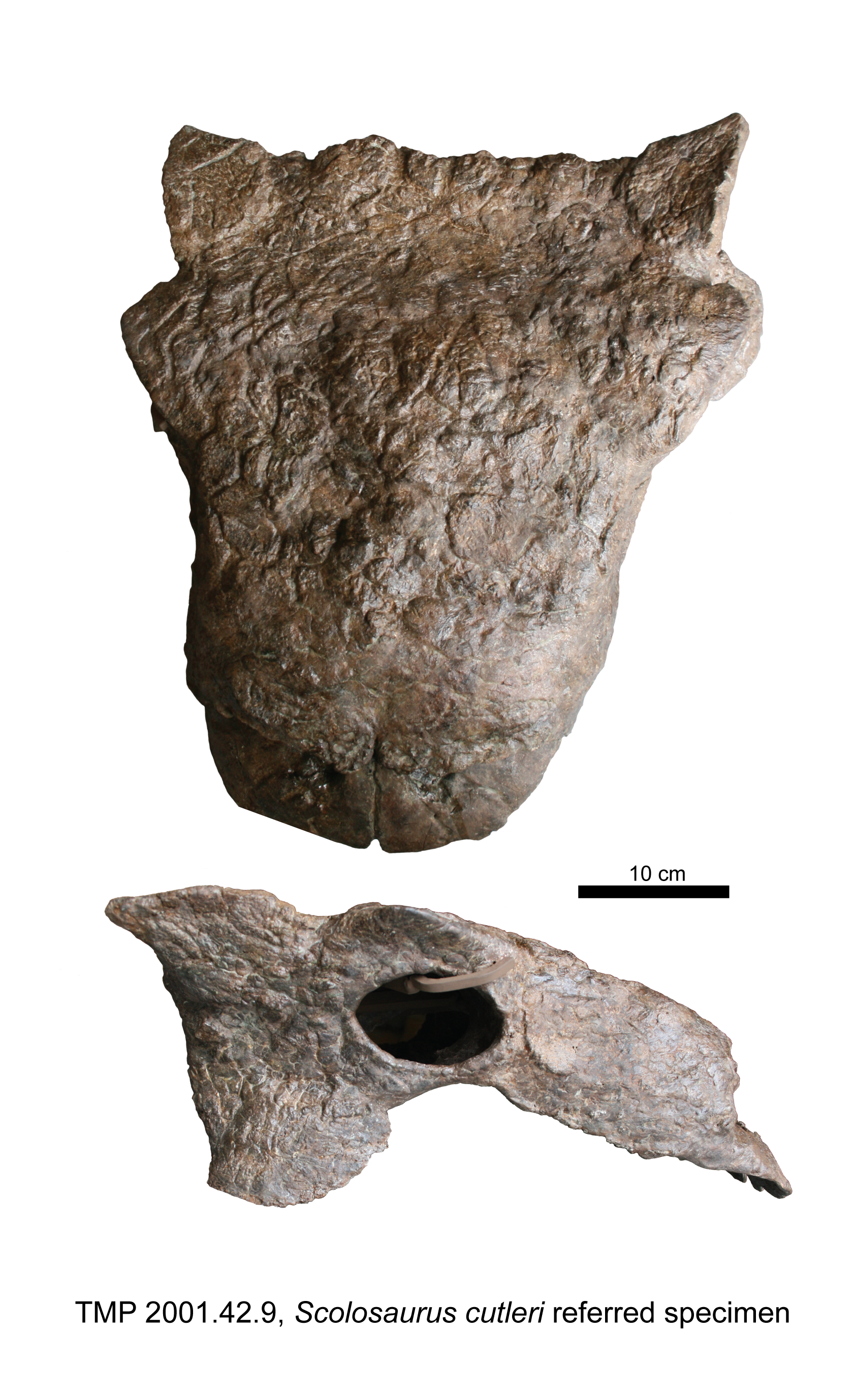

Supplement: Figure S9 — (TIF) [file pone.0062421.s009.tif]

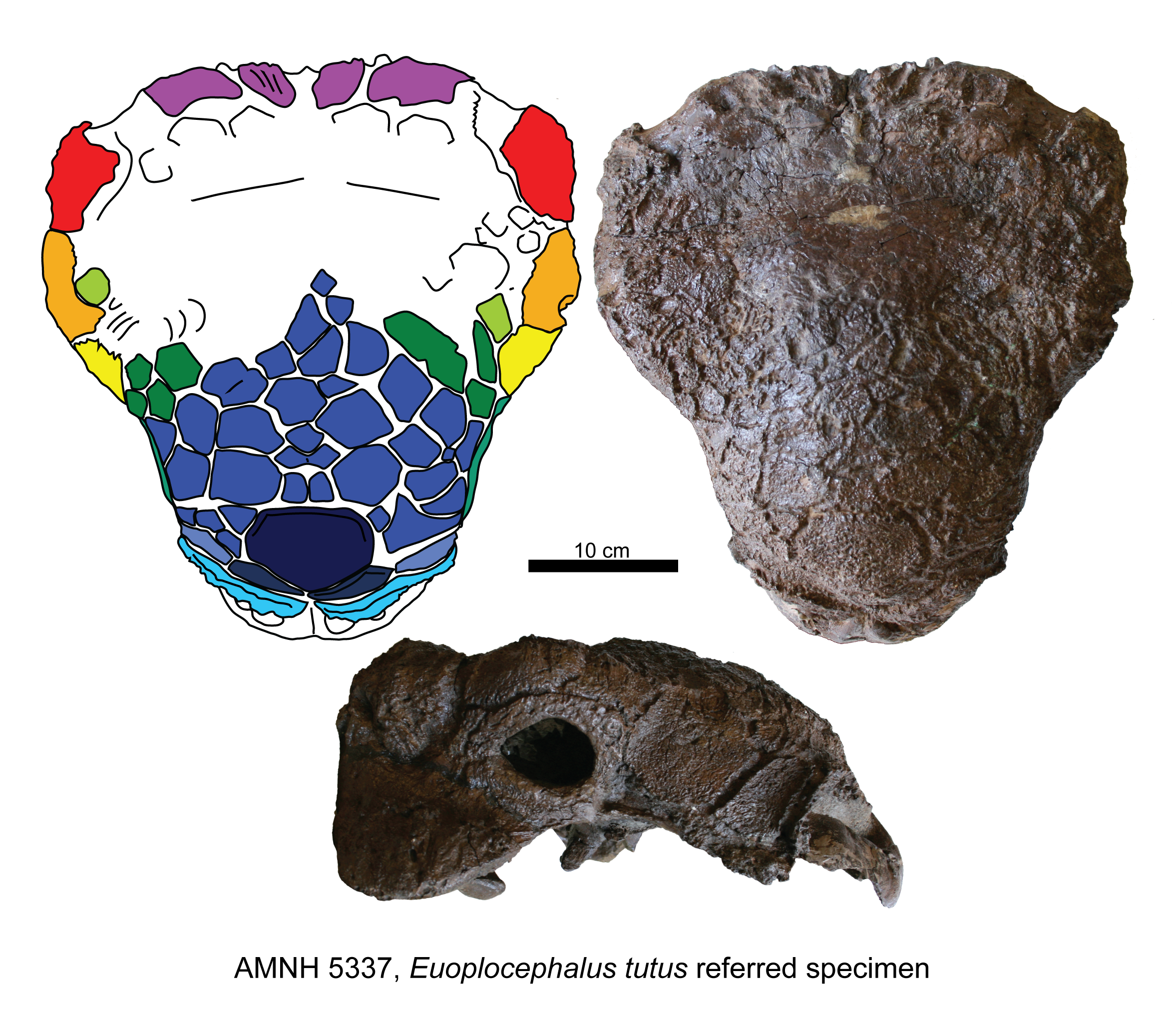

Supplement: Figure S10 — (TIF) [file pone.0062421.s010.tif]

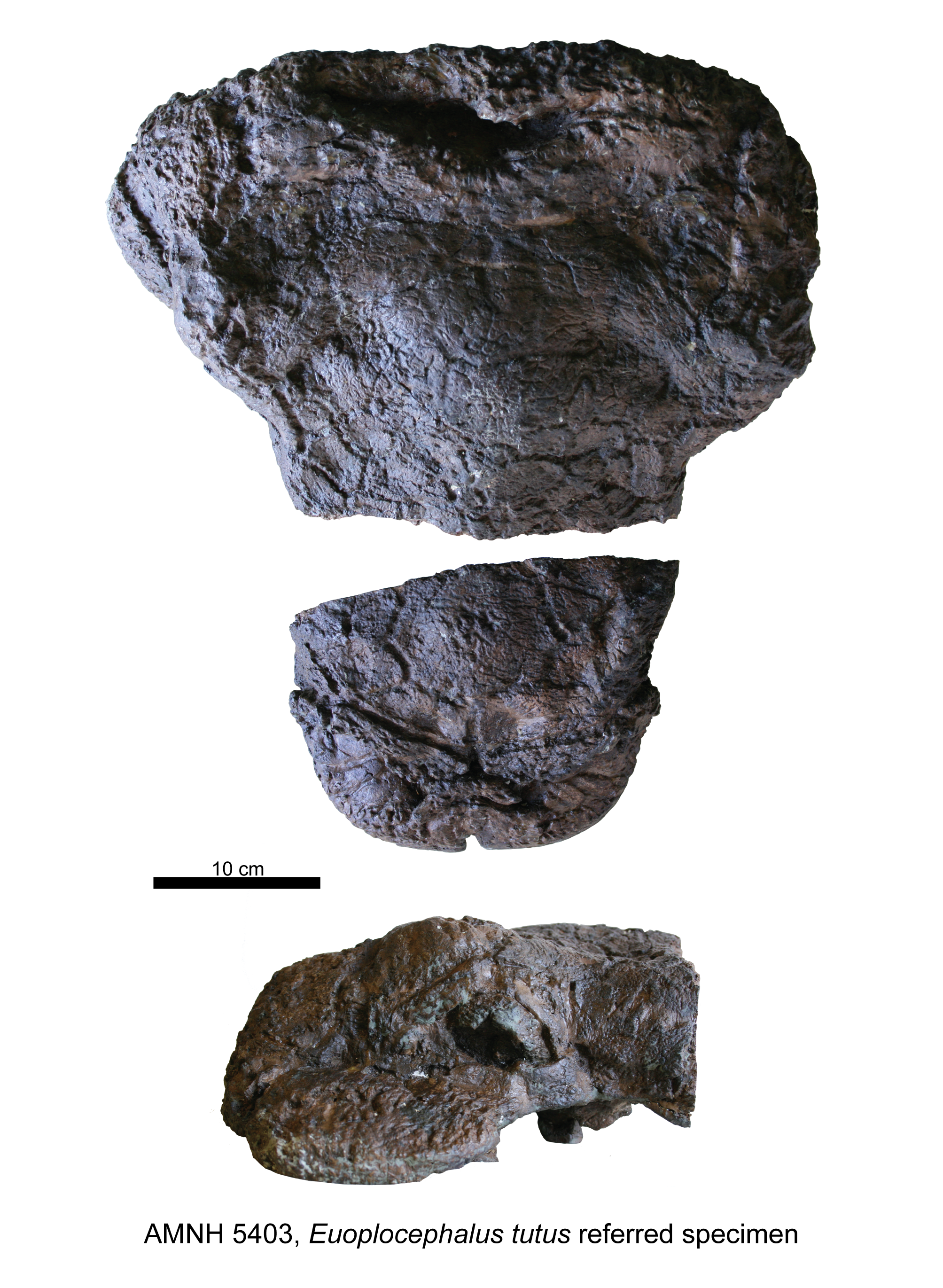

Supplement: Figure S11 — (TIF) [file pone.0062421.s011.tif]

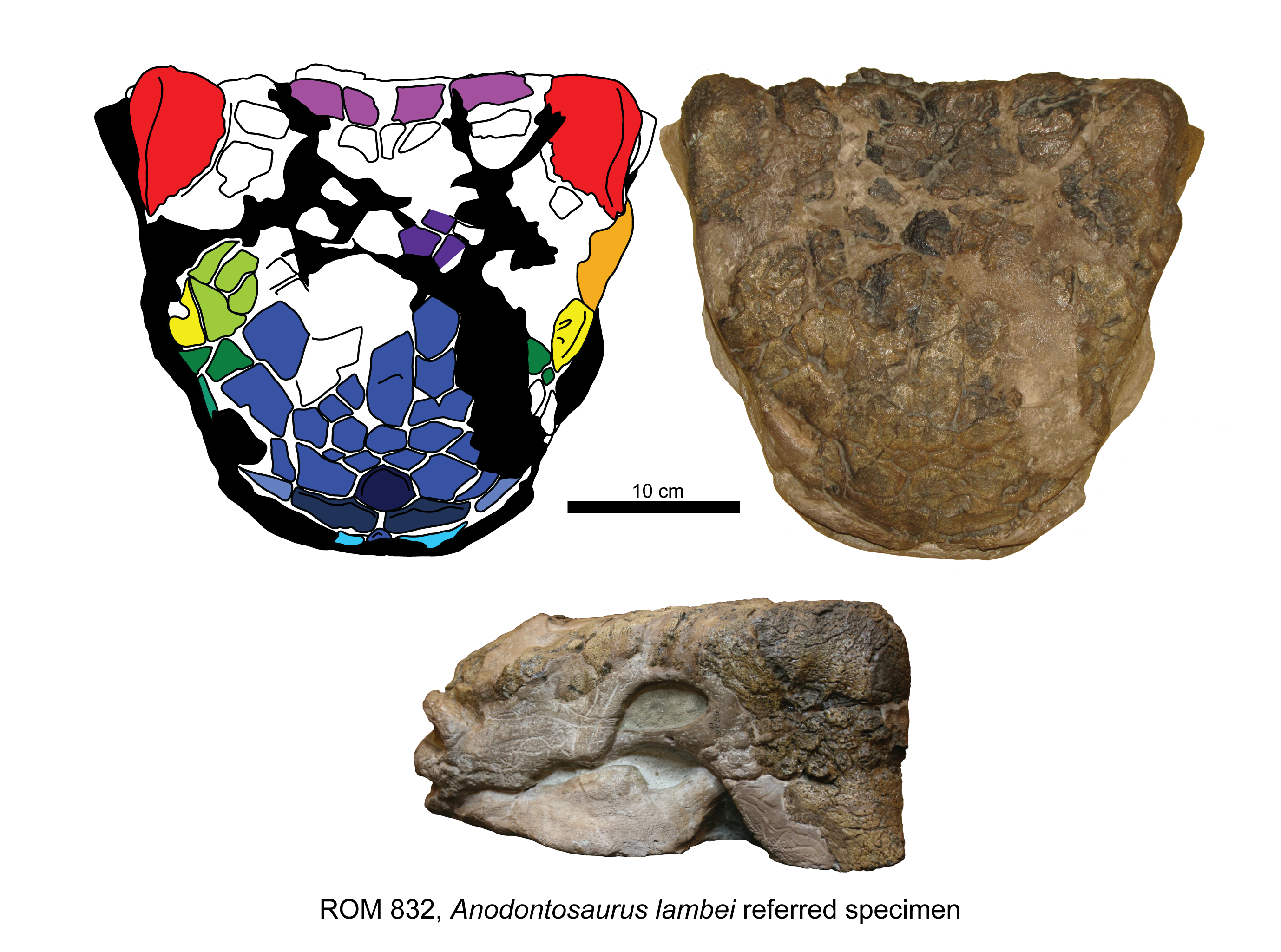

Supplement: Figure S12 — (TIF) [file pone.0062421.s012.tif]

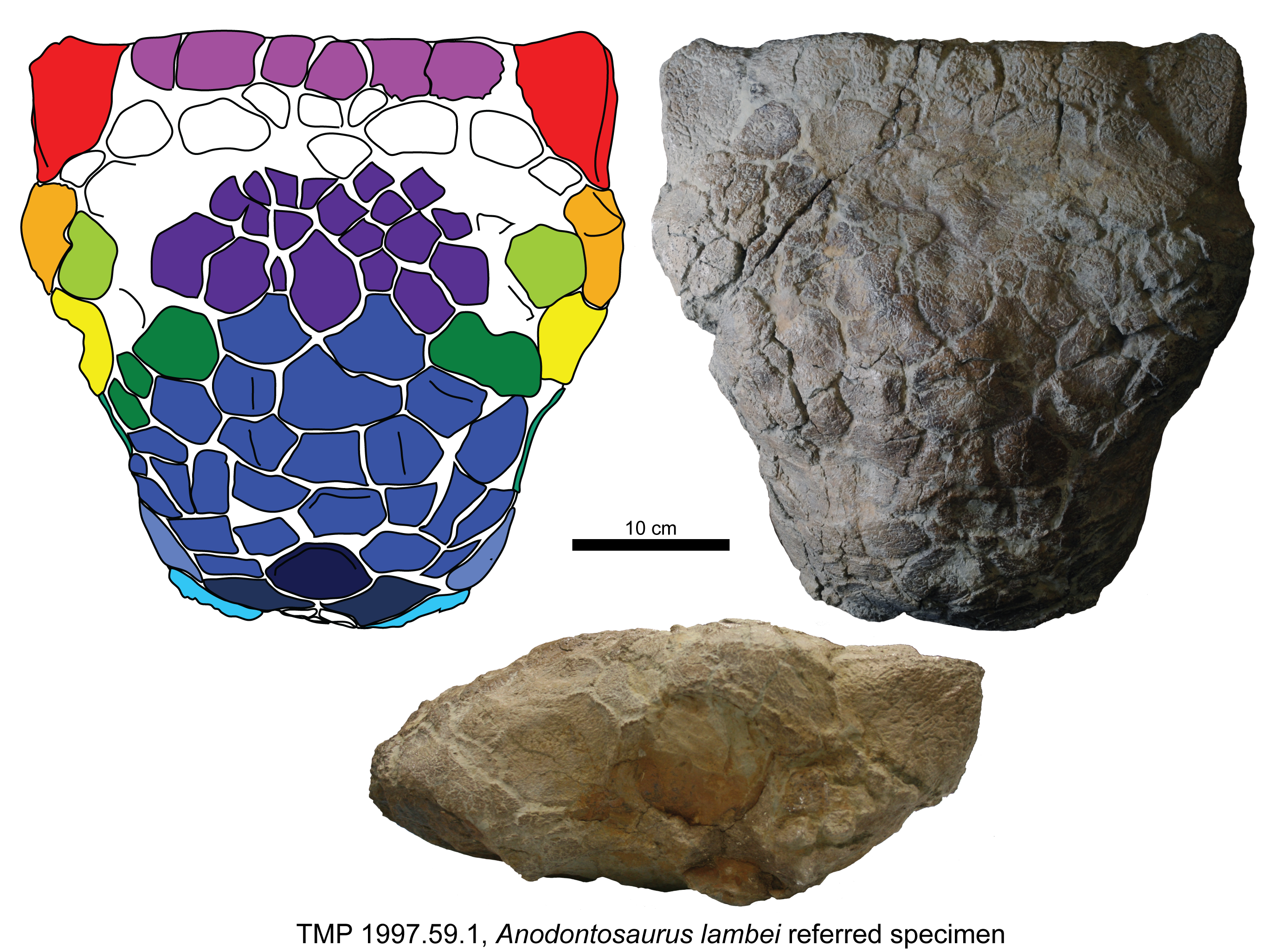

Supplement: Figure S13 — (TIF) [file pone.0062421.s013.tif]

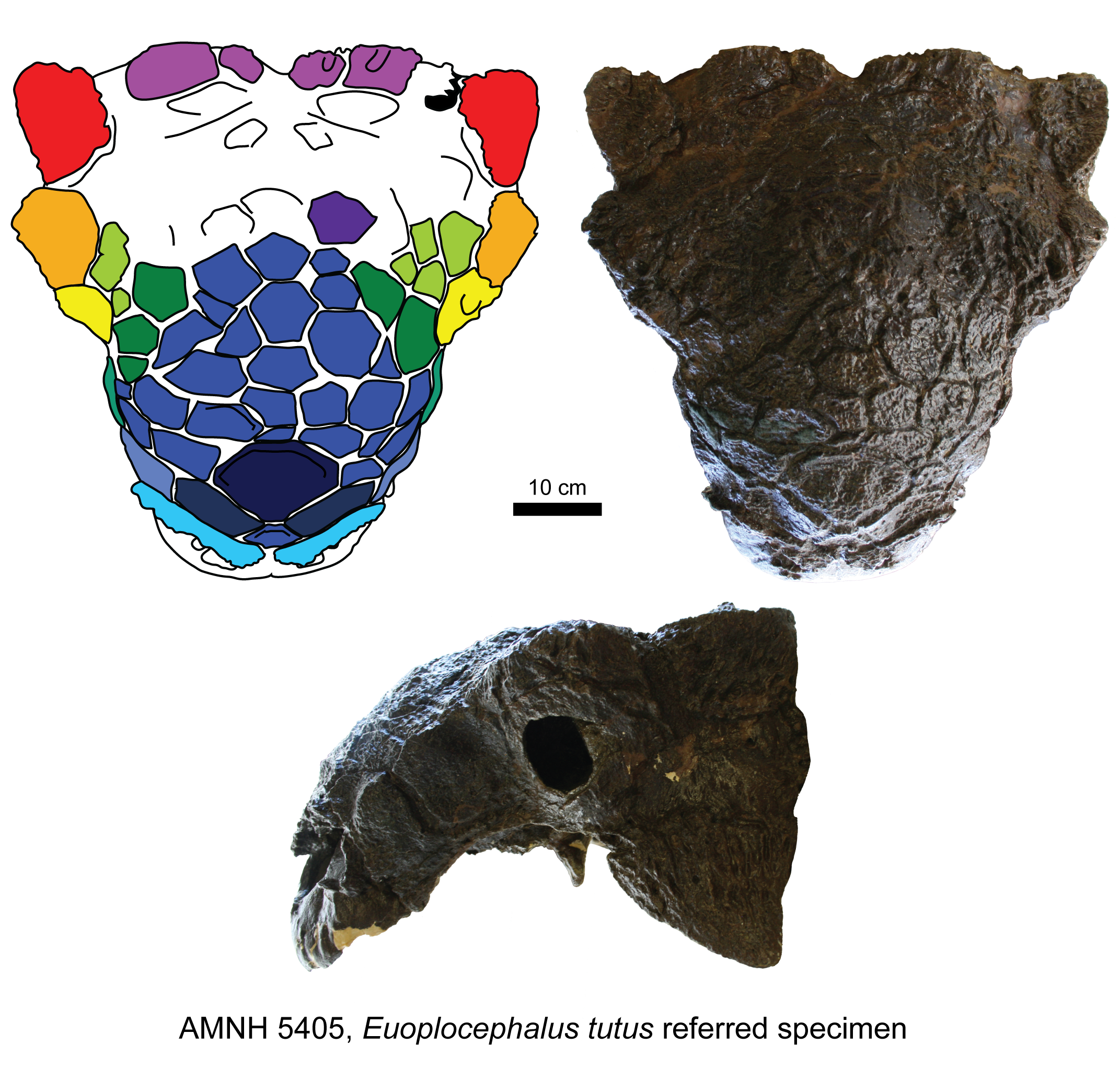

Supplement: Figure S14 — (TIF) [file pone.0062421.s014.tif]

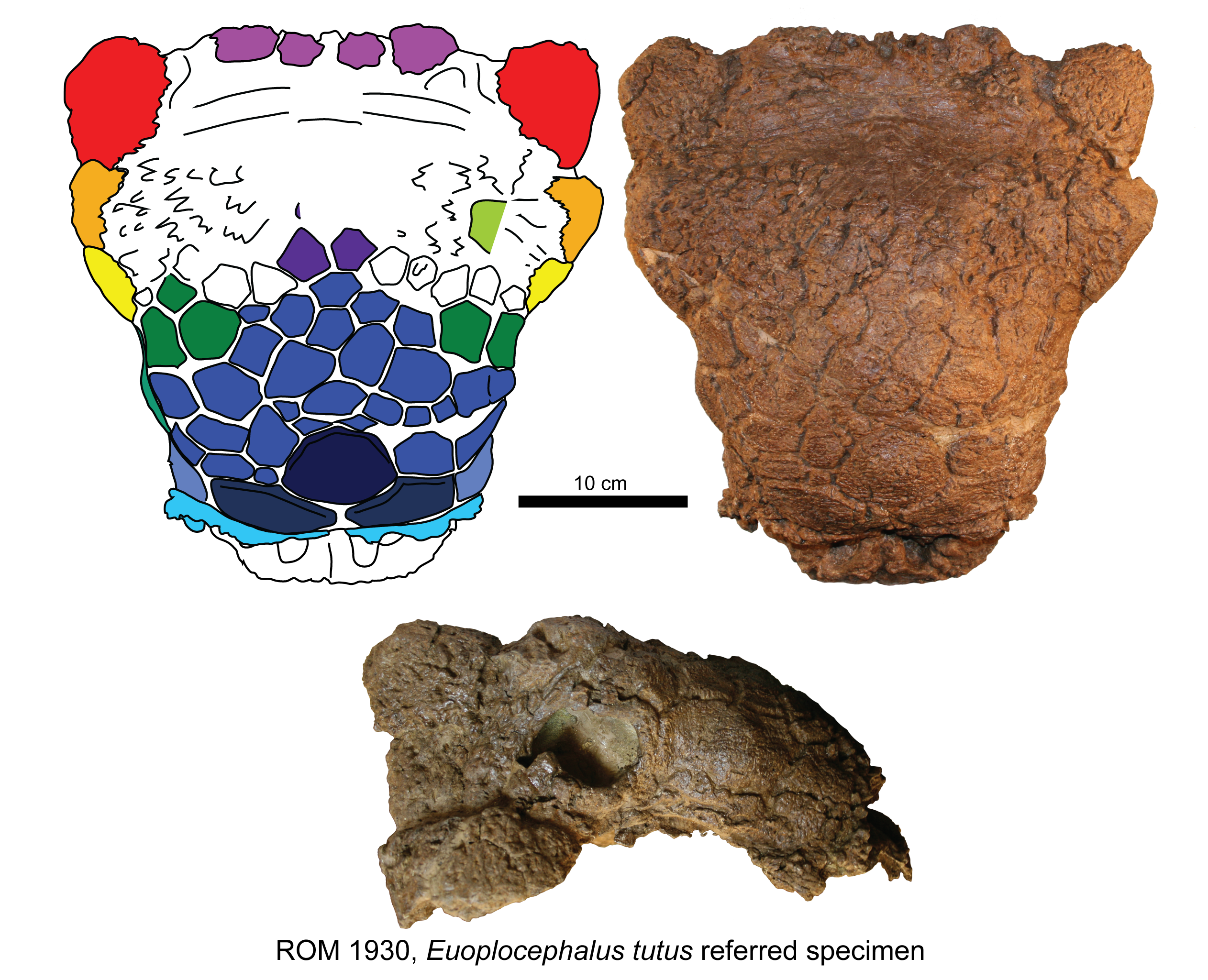

Supplement: Figure S15 — (TIF) [file pone.0062421.s015.tif]

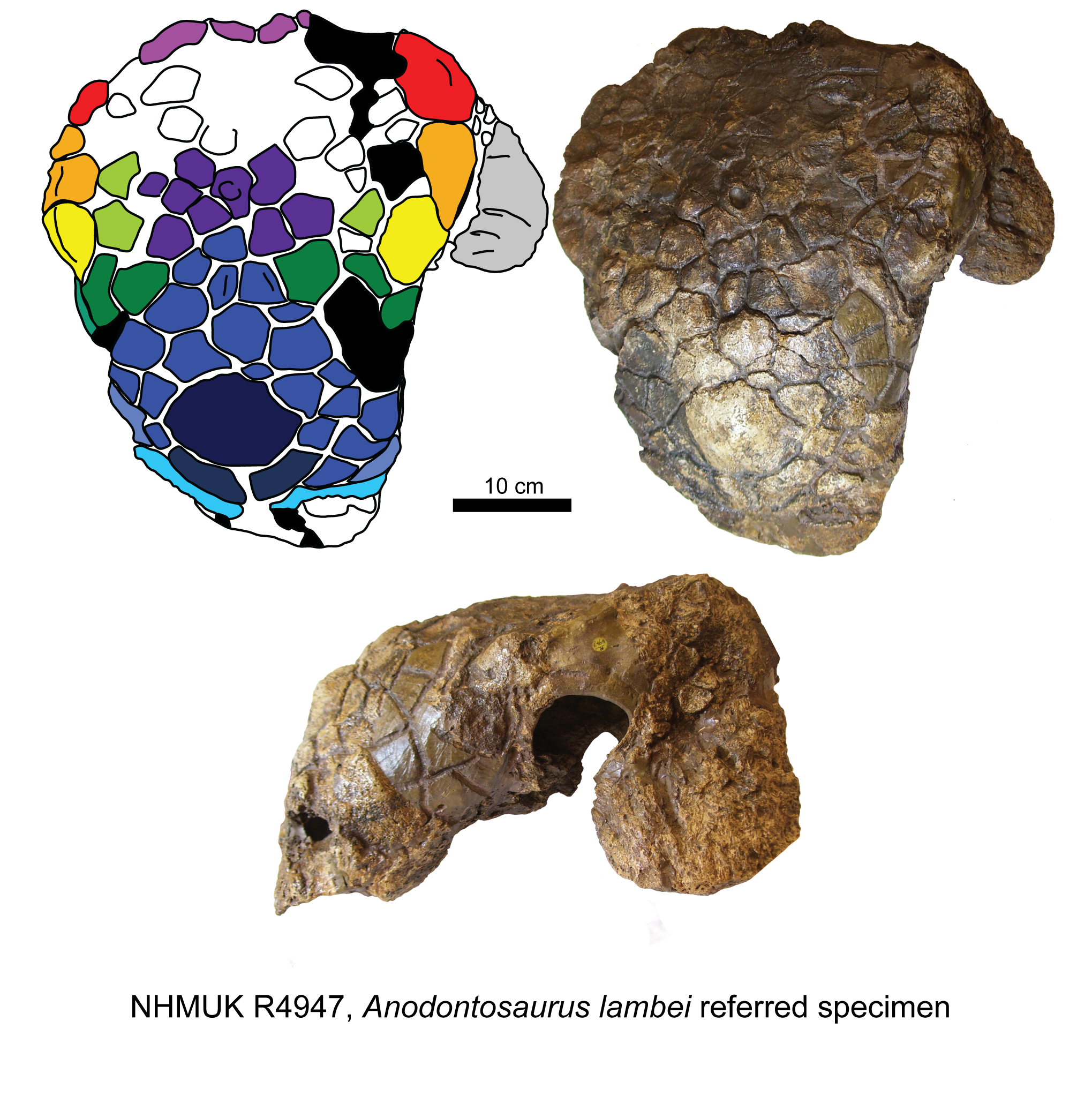

Supplement: Figure S16 — (TIF) [file pone.0062421.s016.tif]

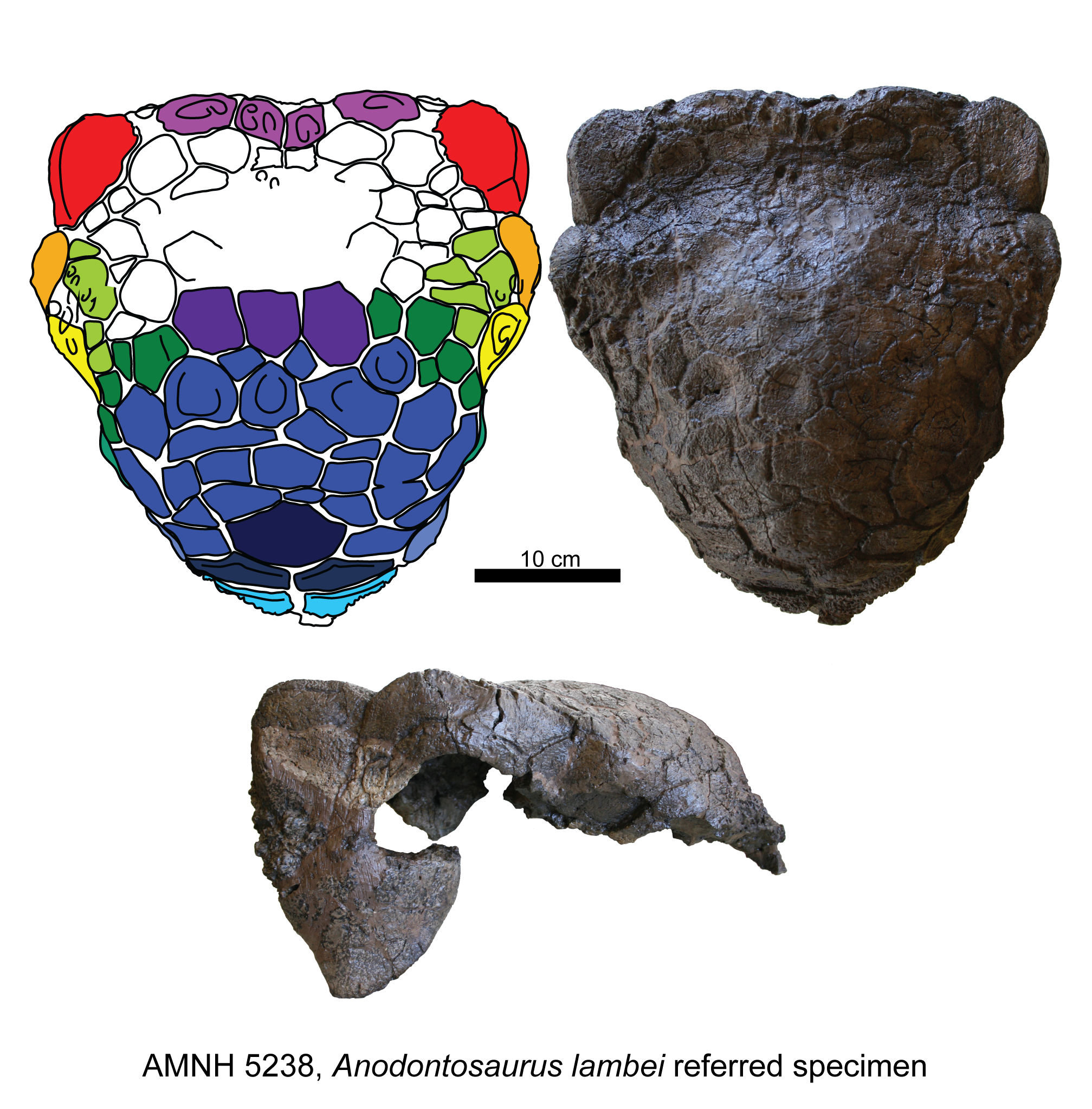

Supplement: Figure S17 — (TIF) [file pone.0062421.s017.tif]

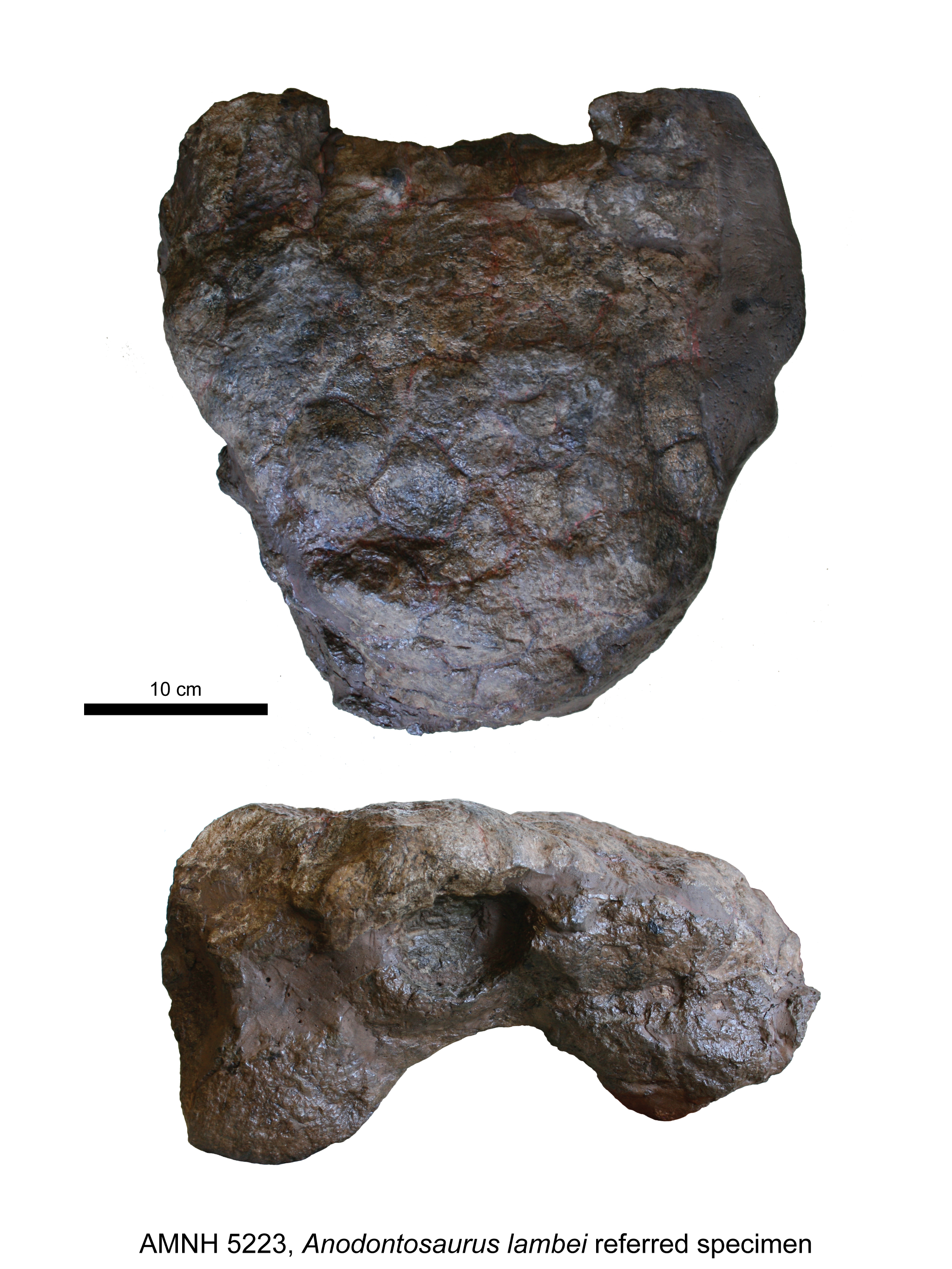

Supplement: Figure S18 — (TIF) [file pone.0062421.s018.tif]

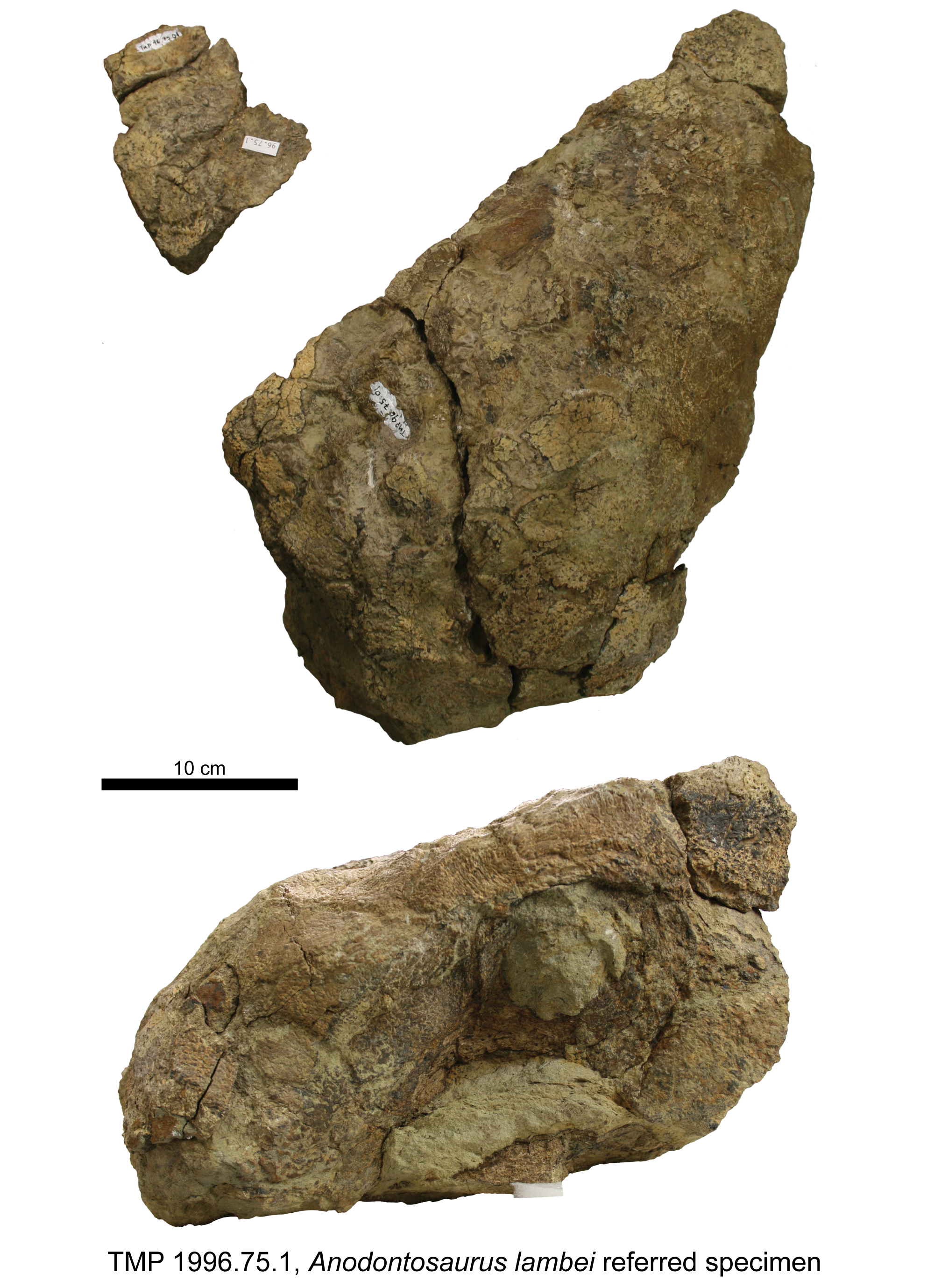

Supplement: Figure S19 — (TIF) [file pone.0062421.s019.tif]
