# Supplementary material for: Euoplocephalus tutus and the Diversity of Ankylosaurid Dinosaurs in the Late Cretaceous of Alberta, Canada, and Montana, USA
Source: PLoS One. 2013 May 8;8(5):e62421. doi: 10.1371/journal.pone.0062421 (PMC3648582; doi:10.1371/journal.pone.0062421)
Supplement: Character Statements S1 — (DOCX) [file pone.0062421.s031.docx]

**CHARACTER STATEMENTS S1**

Character statements and remarks. All character statements are the same as those in Thompson et al. [1], but some character state codings have been altered for the ‘updated codings’ analysis (Matrix 2, Analysis 2). New characters for Matrix 3 and Analysis 3 appear last.

1. Antorbital fenestra: present (0); absent (1). (Sereno [2]: character 8)
2. Lateral temporal fenestra, visible in lateral view: visible (0); hidden (1). (Carpenter et al. [3]: character 6)
3. Supratemporal fenestra: open (0); closed (1). (Lee [4]: character 2)

Updated codings: *Dyoplosaurus* from ? to 1. This region of the skull is preserved in ROM 784.

1. Skull dimensions, including ornamentation: longer than wide (0); as wide, or wider than long (1). (Carpenter et al. [3]: character 1)
2. Width of the posterior margin of the skull relative to the maximum width across the orbits: greater or equal (0); less (1). (Vickaryous et al. [5]: character 6)
3. Size of occiput: higher than wide (0); wider than high (1). (Lee [4]: character 1)

Updated codings: *Nodocephalosaurus* from ? to 1; *Talarurus* from ? to 1. Notes: The occiput is preserved in both *Nodocephalosaurus* and *Talarurus* and so the proportions of height vs. width can be coded.

1. External nares, opening faces: laterally (0); anterolaterally (1); anteriorly (2). (Carpenter et al. [3]: character 10)
2. External nares, visible in dorsal view: visible (0); hidden (1).

Updated codings: *Pinacosaurus grangeri* from 1 to 0.

1. Near vertical narial septum separating the respiratory passage and lateral sinus: absent (0); present (1). (Vickaryous et al. [5]: character 24)
2. Near horizontal narial septum separating the respiratory passage and lateral sinus: absent (0); present (1).
3. Shape of respiratory passage: straight (0); sinuous (1). (Sereno [2]: character 103 )
4. Lateral sinuses: absent (0); present (1). (Sereno [2]: character 95)
5. Orbits, angle of orbital axis: <40º (0); >40º (1).
6. Antorbital region of the dorsal skull surface: flat (0); domed (1). (Sereno [2]: character 99)

Updated codings: *Nodocephalosaurus* from 0 to ?; *Minotaurasaurus* from 1 to 0. Notes: The holotype and only skull of *Nodocephalosaurus* is severely crushed and distorted, and so a dorsoventral dimension-based character coding for this taxon is dubious. As can be seen in lateral view, the skull of *Minotaurasaurus* is flat.

1. Development of the postocular shelf: not developed (0); completely separating orbit from temporal space (1). (Sereno [2]: character 104)
2. Gap between palate and braincase: open (0); closed by a dorsal projection of the pterygoid (1). (Sereno [2]: character 61)

Updated codings: *Tianzhenosaurus* from 1 to ?. Notes: This feature cannot be assessed using the figures or description in Pang and Cheng [6], and the original specimen was not observed for this study.

1. Cranial sutures in adult specimens: visible (0); obliterated (1). (Hill et al. [7]: character 36)

Updated codings: *Dyoplosaurus* from ? to 1; *Nodocephalosaurus* from ? to 1. Notes: Although fragmentary, the skull of *Dyoplosaurus* does not preserve open sutures, and so this character should be coded as “1” for this taxon. In *Nodocephalosaurus*, the cranial sutures are clearly obliterated.

1. Dimensions of premaxillary palate: longer than wide (0); wider than long (1). (Vickaryous et al. [5]: character 13)

Updated codings: *Minotaurasaurus* from ? to 1, *Tianzhenosaurus* from ? to 1.

1. Shape of the premaxillary palate: sub-triangular (0); sub-quadrate (1); sub-oval (2). (Sereno [2]: character 80)
2. ‘V’ or ‘U’-shaped median indentation of the anterior margin of the premaxilla: absent (0); present (1). (Sereno [2]: character 91)

Updated codings: *Ankylosaurus* from 1 to ?, *Minotaurasaurus* from 0 to ?. Notes: This region is broken in the holotype and only specimen of *Minotaurasaurus*, and in all specimens of *Ankylosaurus*.

1. Posteroventral extension of premaxillary tomium in lateral view: ends anterior to the maxillary teeth (0); obscures most anterior maxillary teeth (1). (Sereno [2]: character 100)
2. Bone bordering anterior margin of internal nares: premaxilla (0); maxilla (1).

Updated codings: *Minotaurasaurus* from 0 to ?; *Pinacosaurus mephistocephalus* from 1 to ?, *Tianzhenosaurus* from 1 to ?. Notes: This region is obscured by sediment in the holotype and only skull of *Minotaurasaurus*. It is unclear from the figures in Godefroit et al. [8] of the holotype of *Pinacosaurus mephistocephalus* if the premaxilla or maxilla borders the anterior margin of the internal nares, and so this character is modified from 1 to ?.This feature cannot be assessed using the figures or description in Pang and Cheng [6] of *Tianzhenosaurus*, and the original specimen was not observed for this study.

1. Shape of the ventral margin of premaxillary tomium in lateral view: flat (0); convex (1); concave (2). (Vickaryous et al. [5]: character 12)

Updated codings: *Euoplocephalus* from 1 to 2; *Tianzhenosaurus* from 1 to ?. Notes: The holotype of *Tianzhenosaurus* is broken in this region.

1. Shape of the maxillary tooth row: straight (0); medially convex (1). (Vickaryous et al. [5]: character 18)

Updated codings: *P. grangeri* from 0 to 1; *Tarchia* from 0 to 1; *Tsagantegia* from 0 to 1. Notes: In all of these taxa the maxillary tooth row is at least slightly medially convex.

1. Maxillary tooth row position: lateral margin of skull (0); inset (1). (Lee [4]: character 4)

Updated codings: *Nodocephalosaurus* from ? to 1. Notes: The maxillary tooth row is clearly inset from the lateral margin of the skull in the holotype and only specimen of *Nodocephalosaurus*.

1. Distance between most posterior extent of maxillary tooth rows relative to the width of the premaxillary beak: wider (0); narrower (1). (Sereno [2]: character 102)

Updated codings: *Ankylosaurus magniventris* from 1 to 0. Notes: In AMNH 5214, the beak is narrower than the posterior width between the maxillary tooth rows; see Carpenter [9].

1. Palpebral shape: rod (0); plate (1). (Sereno [2]: character 5)

Updated codings: Changed all ankylosaurs to 1 where skull is known.

1. Form of palpebral articulation: mobile contact with prefrontal (0); extensive sutural contact with prefrontal, frontal and postorbital (1). (Sereno [2]: character 9)
2. Anterior and posterior supraorbitals (recognizable by distinct regions of ornamentation above the orbit): absent (0); present (1). (Sereno [2]: character 13)
3. Form of supraorbital ornamentation: boss-like, rounded laterally (0); sharp lateral rim, forming a ridge (1). (Vickaryous et al. [5]: character 5)

Updated codings: *Dyoplosaurus* from 1 to ?. Notes: The supraorbitals are poorly preserved in the holotype and only specimen of *Dyoplosaurus*.

1. Form of the parietal surface: not domed (0); domed (1). (Lee [4]: character 24)

Updated codings: *Dyoplosaurus* from 1 to 0. Notes: Although the posterior region of the skull of *Dyoplosaurus* was described as ‘domed’ in Arbour et al. [10], the domed region more likely represents the frontals, not the parietals.

1. Proportions of jugal orbital ramus: depth greater than transverse breadth (0); transverse breadth greater than depth (1). (Sereno [2]: character 1)

Updated codings. *Minotaurasaurus* from ? to 1. Notes: This feature is preserved in *Minotaurasaurus*.

1. Shape of quadrate in lateral aspect: curved (anteriorly convex, posteriorly concave) (0); straight (1). (Vickaryous et al. [5]: character 38)

Updated codings: *Minotaurasaurus* from ? to 1; *Nodocephalosaurus* from ? to 1. Notes: This feature is preserved in both of these skulls and therefore can be coded.

1. Inclination of quadrate in lateral aspect: near vertical (0); almost 45º anterolaterally (1). (Lee [4]: character 10)
2. Form of the anterior surface of the quadrate: transversely concave (0); not concave (1). (Lee [4]: character 12)

Updated codings: *Minotaurasaurus* from ? to 1. Notes: This feature is preserved in *Minotaurasaurus*.

1. Ventral projection of the mandibular process of the quadrate in lateral view: projects beyond the quadratojugal ornamentation (0); hidden by quadratojugal ornamentation (1). (Vickaryous et al. [5]: character 40)

Updated codings: *Nodocephalosaurus* from ? to 1. Notes: This feature is preserved in *Nodocephalosaurus*. Although the specimen is distorted, it is apparent that the quadrate was obscured by the quadratojugal horn in lateral view.

1. Form of quadrate mandibular extremity: symmetrical (0); medial condyle larger than lateral condyle (1). (Sereno [2]: character 10)

Updated codings: *Nodocephalosaurus* from ? to 1. Notes: This feature is preserved in *Nodocephalosaurus*.

1. Inclination of the articular surface of the quadrate condyle in posterior view: horizontal (0); ventromedially inclined at approximately 45° to horizontal (1). (Sereno [2]: character 14)

Updated codings: *Minotaurasaurus* from 0 to 1.

1. Lateral ramus of the quadrate: present (0); absent (1). (Sereno [2]: character 15)
2. Dorsoventral depth of the pterygoid process of the quadrate: deep (0); shallow (1). (Sereno [2]: character 60; Lee [4]: character 7)
3. Contact between paroccipital process and quadrate: sutural (0); fused (1). (Carpenter et al. [3]: character 13)
4. Contact between pterygoids: pterygoids separate posteromedially, forming an interpterygoid vacuity (0); pterygoids joined medially forming a pterygoid shield (1).
5. Direction of the pterygoid flange: anterolateral (0); anterior/parasagittal (1). (Vickaryous et al. [5]: character 29)
6. Contact between basipterygoid processes and pterygoid: sutural (0); fused (1). (Vickaryous et al. [5]: character 30)
7. Position of ventral margin of the pterygovomerine keel relative to alveolar ridge: dorsal (0); level (1). (Sereno [2]: character 59)

Updated coding: *Minotaurasaurus* from 0 to ?, *Tianzhenosaurus* from 1 to ?. Notes: The pterygovomerine keel is broken in *Minotaurasaurus*, and so this character cannot be coded for this taxon. The extent and preservation of the vomer is unclear from the figures in Pang and Cheng [6].

1. Dorsal extent of median vomer lamina: does not meet skull roof (0); meets skull roof (1). (Lee [4]: character 14)
2. Pterygoid foramen: absent (0); present (1). (Hill et al. [7]: character 21)

Updated coding: *Tarchia* from 0 to 1, *Tianzhenosaurus* from 0 to ?. Notes: This feature is present in the holotype and only known skull of *Tarchia*. It is unclear from the figures in Pang and Cheng [6] if this feature is present in *Tianzhenosaurus*.

1. Position of posterior margin of pterygoid body relative to the anterior margin of the quadrate condyle: anteriorly positioned (0); in transverse alignment (1). (Vickaryous et al. [5]: character 28)

Updated codings: *Tianzhenosaurus* from 0 to 1.

1. Posteroventral secondary palate: absent (0); present (1).
2. Posterior palatal foramen: absent (0); present (1). (Lee [4]: character 17)

Updated codings: *Saichania* from 0 to 1, Tsagantegia from ? to 0, *Tianzhenosaurus* from 0 to ?.

1. Direction of paroccipital process extension: posterolateral (0); lateral (1). (Carpenter et al. [3]: character 11; Lee [4]: character 33)
2. Depth of the distal end of paroccipital processes: expanded (0); not expanded (1).

Updated codings: *Saichania* from 0 to 1; *Talarurus* from 0 to ?; *Tsagantegia* from ? to 0. Notes: The paroccipital processes are not noticeably expanded in *Saichania*, although the definition of ‘expanded’ versus ‘not expanded’ should probably be clarified. The paroccipital processes are broken in the holotype skull PIN 557 of *Talarurus* and in referred specimen PIN 3780/1. They are slightly expanded in *Tsagantegia*, although again this could be better described as being downturned distally.

1. Thickness of bone at the dorsal margin of the foramen magnum relative to surrounding bone: little difference (0); distinctly thickened (1).

Updated codings: *Euoplocephalus* from 0 to 1.

1. Bones forming the occipital condyle: basioccipital and exoccipital (0); basioccipital only (1). (Lee [4]: character 9)

Updated codings: *Ankylosaurus* from 0 to 1, *Euoplocephalus* from 0 to 1, *Minotaurasaurus* from ? to 1, *Nodocephalosaurus* from 0 to 1, *Saichania* from 0 to 1, *Tarchia* from 0 to 1, *Talarurus* from 0 to 1, Tsagantegia from 0 to 1, *Tianzhenosaurus* from 0 to ?.

1. Form of the ventral surface of basioccipital-basisphenoid region of the braincase: transversely convex (0); has a medial depression (1); has a medial longitudinal ridge (2).
2. Length of basisphenoid relative to the basioccipital: longer (0); shorter or equal (1). (Sereno [2]: character 12)

Updated codings: *Minotaurasaurus* from ? to 1. Notes: The basisphenoid and basioccipital are preserved in *Minotaurasaurus*, and so this feature can be coded.

1. Form of basisphenoidal tuberosities: medially separated rounded rugose stubs (0); continuous transverse rugose ridge (1). (Vickaryous et al. [5]: character 32)
2. Size of basipterygoid processes: twice as long as wide or over (0); less than twice as long as wide (1).

Updated codings: *Minotaurasaurus* from ? to 1, Tsagantegia from ? to 1. Notes: The basipterygoid processes are preserved in *Minotaurasaurus* and *Tsagantegia*, and so this feature can be coded.

1. Form of the cranial nerve foramina IX-XII: separate foramina (0); single foramen shared with the jugular vein (1).
2. Degree of endocranial flexure: strong (0); weak (1).
3. Direction of occipital condyle: posterior (0); posteroventral (1). (Vickaryous et al. [5]: character 36)

Updated codings: *Tarchia* from 0 to 1.

1. Direction of the foramen magnum: posterior (0); posteroventral (1). (Vickaryous et al. [5]: character 37)
2. Premaxillary teeth: present (0); absent (1). (Sereno [2]: character 18)
3. Cingula on maxillary and/or dentary teeth: absent (0); present (1). (Carpenter et al. [3]: character 21)

Updated codings: *Tianzhenosaurus* from 1 to ?. It is unclear from the figures in Pang and Cheng [6] if this feature is present in *Tianzhenosaurus*.

1. Maxillary and/or dentary tooth crown shape: ≥13 denticles, tooth crown pointed (0); <13 denticles, tooth crown rounded (1).
2. Number of dentary teeth: <25 (0); ≥25 (1).

Updated codings: *Tsagantegia* from ? to 0; *Tianzhenosaurus* from 0 to ?. Notes: Pang and Cheng ([6]:330) state that “…13 relatively complete teeth and three broken tooth bases are preserved”, but it is unclear if this refers only to preserved teeth and partial teeth, or tooth alveoli. This also cannot be determined from the figures in Pang and Cheng [6].

1. Position of mandible articulation relative to mandibular adductor fossa: posterior (0); posteromedial (1). (Sereno [2]: character 64)

Updated codings: *Tianzhenosaurus* 1 to ?. Notes: The mandible is preserved but not figured.

1. Mandibular fenestra: present (0); absent (1).

Updated codings: *Tianzhenosaurus* 1 to ?. Notes: The mandible is preserved but not figured, and the presence or absence of the fenestra is not mentioned.

1. Depth of the dentary symphysial ramus relative to half the maximum depth of the mandibular ramus in lateral view: deeper (0); shallower (1). (Sereno [2]: character 17)

Updated codings: *Minotaurasaurus* from ? to 1, *Tianzhenosaurus* 1 to ?. Notes: Both dentaries are preserved in *Minotaurasaurus*, and so this feature can be coded. The mandible is preserved in *Tianzhenosaurus* but not figured, and this feature is not explicitly described.

1. Shape of dorsal margin of the dentary in lateral view: straight (0); sinuous (1). (Sereno [2]: character 4)

Updated codings: *Tianzhenosaurus* 1 to ?. Notes: The dentary is preserved but not figured, and this feature is not explicitly described.

1. Shape of ventral margin of the dentary in lateral view: straight (0); sinuous (1). (Sereno [2]: character 85)

Updated codings: *Euoplocephalus* from 1 to 0, *Tianzhenosaurus* 1 to ?. Notes: The dentary is preserved in *Tianzhenosaurus* but not figured, and this feature is not explicitly described.

1. Shape of the alveolar margin: weakly convex (0); strongly convex (1).
2. Development of the coronoid process: not developed (0); distinct (1). (Sereno [2]: character 108)
3. Position of glenoid for quadrate relative to mandibular axis: medially offset (0); in line (1). After Carpenter et al. [11].
4. Size and projection of the retroarticular process: small with no dorsal projection (0); well developed with a dorsal projection (1).
5. Size of predentary ventral process: distinct, prong-shaped process (0); rudimentary eminence (1). (Sereno [2]: character 66)
6. Ornamentation, defined as sculpturing of skull bones or addition of osteoderms (caputegulae): absent (0); present (1). (Sereno [2]: character 63)
7. Cranial armour pattern: amorphous, rugose (0); pattern of polygons covering the skull roof (1). After Carpenter et al. [11].

Updated codings: *Tsagantegia* from 0 to 1; *Dyoplosaurus* from 0 to ?. Notes: Although the drawings of the holotype skull (MPC 700/17) of *Tsagantegia* in Tumanova [12] show amorphous cranial ornamentation, firsthand examination of the skull indicates that there are distinct low-relief polygons covering the skull roof. In the holotype of *Dyoplosaurus*, only the parietal region of the skull is preserved. Distinct polygons are generally not present in this region of the skull in *Euoplocephalus* and it is possible that *Dyoplosaurus* was similar in this regard. As such, this feature cannot be coded for *Dyoplosaurus*.

1. Distribution of polygons on skull roof: random (0); symmetrical (1).
2. A single large medial polygon of ornamentation in the parietal region: absent (0); present (1).

Updated codings: *Dyoplosaurus* from ? to 0. Notes: The parietal region of *Dyoplosaurus* is preserved, allowing this character to be coded.

1. A single medial polygon located posteriorly to the external nares: absent (0); present (1). (Vickaryous et al. [5]: character 9)
2. Surface of polygonal ornamentation on the dorsal surface of the skull: flat (0); domed (1). After Sullivan [13].

Updated codings: *P. grangeri* from ? to 1; *Talarurus* from 0 to 1. Notes: Juvenile *P. grangeri* lack polygonal ornamentation on the dorsal surface of the skull, but the holotype skull AMNH 6523, of a larger, presumably adult individual, clearly preserves domed caputegulae on the skull surface. The holotype of *Talarurus* PIN 557 preserves distinctly domed cranial caputegulae.

1. Projection of postorbital/squamosal ‘horns’ relative to the posterior margin of the dorsal surface of the skull: horns end anteriorly (0); horns extend posteriorly beyond skull roof (1).

Updated codings: *Nodocephalosaurus* from ? to 1, *Tianzhenosaurus* from 0 to 1. Notes: Although the skull is crushed, the squamosal horns clearly project posteriorly beyond the nuchal shelf of *Nodocephalosaurus*.

1. Postorbital/squamosal ‘horn’: absent (0); present (1). (Lee [4]: character 18)
2. Shape of postorbital/squamosal ‘horn’: rounded (0); pyramidal (1). (Vickaryous et al. [5]: character 6)
3. Quadratojugal ‘horn’: absent (0); present (1). After Carpenter et al. [11].
4. Shape of quadratojugal ‘horn’: rounded (0); pyramidal (1).
5. Raised nuchal sculpturing, defined as a transversely expanded region of ornamentation at the posterior margin of the skull roof: absent (0); present (1). (Vickaryous et al. [5]: character 11)

Updated codings: *Minotaurasaurus* from 0 to 1; *Dyoplosaurus* from ? to 1. Notes: The nuchal shelf is preserved in both of these taxa, and both taxa have raised nuchal sculpturing.

1. Posterior projection of the nuchal shelf: does not obscure occiput in dorsal view (0); obscures occiput in dorsal view (1). (Vickaryous et al. [5]: character 12)
2. Length of mandibular osteoderm (caputegulum) with respect to the length of the mandible: less than or equal to half the length (0); over three quarters the length (1). After Carpenter et al. [11].
3. Mandibular osteoderm (caputegulum): absent (0); present (1).
4. Type of articulation between the atlantal neural arch and intercentrum: open (0); fused in adult (1). (Sereno [2]: character 19)

Updated codings: *Tianzhenosaurus* 1 to ?.

1. Type of contact between the atlantal neural arches: no median contact (0); median contact (1). (Sereno [2]: character 68)

Updated codings: *Tianzhenosaurus* 1 to ?.

1. Contact between atlas and axis: articulated (0); fused (1). (Vickaryous et al. [5]: character 46)
2. Dimensions of cervical vertebrae centra: anteroposteriorly longer than transverse width (0); anteroposteriorly shorter than transverse width (1). After Kirkland et al. [14].

Updated codings: *Tianzhenosaurus* 1 to ?.

1. Ratio of maximum neural spine width to height in anterior cervicals: <0.25 (0); ≥0.25 (1). After Carpenter et al. [11].

Updated codings: *Tianzhenosaurus* 1 to ?.

1. Alignment of anterior and posterior faces of cervical centra: aligned (0); anterior face dorsal to posterior face (1); anterior face ventral to posterior face (2). (Vickaryous et al. [5]: character 47)
2. Ratio of anteroposterior (dorsal vertebra) centrum length to posterior centrum height: >1.1 (0); <1.1 (1).

Updated codings: *Tianzhenosaurus* 1 to ?.

1. Longitudinal keel on ventral surface of dorsal centra: present (0); absent (1).

Updated codings: *P. grangeri*from ? to 1. Notes: a longitudinal keel is present on the ventral surface of dorsal centra in referred *P. grangeri* specimen PIN 614.

1. Cross sectional shape of neural canal in posterior dorsals: circular (0) elliptical, with long axis running dorsoventrally (1). After Carpenter [15].

Updated codings: *Saichania* from 0 to ?. Notes: Although a second specimen (MPC 100/1305) has been referred to *Saichania* by Carpenter et al. [16], it is currently unclear if there are shared diagnostic features between this specimen and the holotype (MPC 100/151). Additionally, although dorsal vertebrae are preserved in the holotype, they were not figured by Maryańska [17] and no reference is made to the shape of the neural canal. The postcrania of MPC 100/151 was not examined firsthand by VMA or PJC and so this character cannot be verified for *Saichania*.

1. Shape of the proximal cross-section of the dorsal ribs: triangular (0); ‘L’- or ‘T’-shaped (1).

Updated codings: *Tianzhenosaurus* 1 to ?.

1. Attachment of dorsal ribs to posterior dorsal vertebrae: articulated (0); fused (1).

Updated codings: *Tianzhenosaurus* 1 to ?.

1. Contact between most posterior dorsal vertebrae: articulated (0); fused to form a presacral rod (1).
2. Paravertebrae: absent (0); present (1).
3. Longitudinal groove in ventral surface of the sacrum: absent (0); present (1).

Updated codings: *Tianzhenosaurus* 1 to ?.

1. Number of sacral vertebrae: 5 (0); 4 (1); 3 (2). (Sereno [2]: character 69)
2. Ratio of maximum distal width to height of the neural spines of proximal caudals: ≤0.2 (0); >0.2 (1). After Carpenter [18].

Updated codings: *Tianzhenosaurus* 1 to ?.

1. Direction of the transverse processes of proximal caudals: anterolaterally projecting (0); posterolaterally projecting (1); laterally projecting (2). After Carpenter [18].

Updated codings: *Ankylosaurus magniventris* from 2 to 0; *Edmontonia* 0 to 2, *Euoplocephalus* from 1 to 0; *Nodocephalosaurus* from 1 to 0. Notes: In *Ankylosaurus*, *Euoplocephalus*, and *Nodocephalosaurus*, the transverse processes of the free caudals project anterolaterally from the centrum. In *Edmontonia*, the transverse processes project laterally.

1. Length of transverse processes relative to neural spine height in proximal caudals: sub-equal (0); approximately twice the length (1). (Sereno [2]: character 70)

Updated codings: *Euoplocephalus* from 1 to 0, *Tianzhenosaurus* 1 to ?. Notes: Although the proportions may change slightly along the vertebral column, the transverse processes are usually almost as long as the neural spine is high in *Euoplocephalus*.

1. Persistence of transverse processes down the length of the caudal series: not present beyond the mid-length of the series (0); present beyond the mid-length of the series (1).
2. Attachment of haemal arches to their respective centra: articulated (0); fused (1).

Updated codings: *Nodocephalosaurus* from 0 to ?; *P. grangeri* from ? to 1, *Tianzhenosaurus* from 1 to ?. Notes: Caudal vertebrae have been referred to *Nodocephalosaurus* by Sullivan and Fowler [19], but these were isolated elements unassociated with other, more diagnostic material, from the same formation as the holotype specimen. As such, it seems best to code this character as unknown for *Nodocephalosaurus* at present. Caudal centra with preserved haemal arches are present in *P. grangeri* referred specimen PIN 614.

1. Shape of distal caudal postzygapophyses: short with a sub-triangular end (wedge-shaped) (0); long with a rounded end (tongue shaped) (1). (Sereno [2]: character 110)

Updated codings: *Dyoplosaurus* from ? to 1, *Pinacosaurus mephistocephalus* from ? to 1, *Saichania* from 1 to ?. A tail club is preserved in ROM 784 and has the typical handle vertebrae of other ankylosaurids. A tail club is also preserved in *Pinacosaurus mephistocephalus* and has modified distal caudal vertebrae. An isolated tail club has been referred to *Saichania*, but no tail club is preserved with the holotype material.

1. Extent of pre- and postzygapophyses over their adjacent centra in posterior vertebrae: extend over less than half the length of the adjacent centrum (0); extend over more than half the length of the adjacent centrum (1). (Sereno [2]: character 109)

Updated codings: *Saichania* from 1 to ?. Notes: An isolated tail club has been referred to *Saichania*, but no tail club is preserved with the holotype material.

1. Shape of the posterior haemal arches: rounded haemal spine in lateral view with no contact between haemal arches (0); inverted ‘T’-shaped haemal spine in lateral view, with contact between the ends of adjacent spines (1). (Sereno [2]: character 71)

Updated codings: *Pinacosaurus mephistocephalus* from ? to 1, *Saichania* from 1 to ?, *Tianzhenosaurus* from 1 to ?. A tail club handle is preserved in *Pinacosaurus mephistocephalus*. An isolated tail club has been referred to *Saichania*, but no tail club is preserved with the holotype material. The tail club of *Tianzhenosaurus* was not figured or described.

1. Ossified tendons in distal region of tail: absent (0); present (1). (Sereno [2]: character 97)

Updated codings: *Saichania* from 1 to ?, *Tianzhenosaurus* from 1 to ?. An isolated tail club has been referred to *Saichania*, but no tail club is preserved with the holotype material.

1. Dimensions of coracoid: longer than wide (0); wider than long or equal width and length (1).
2. Form of the anterior margin of the coracoid: convex (0); straight (1).
3. Anteroventral process of coracoid: absent (0); present (1).
4. Size of coracoid glenoid relative to scapula glenoid: sub-equal (0); half the size (1). (Sereno [2]: character 89)

Updated codings: *Pinacosaurus grangeri* from ? to 0.

1. Contact between scapula and coracoid: articulated (0); fused (1).
2. Scapula glenoid orientation: ventrolateral (0); ventral (1). (Sereno [2]: character 87)

Updated codings: *Tianzhenosaurus* from 1 to ?.

1. Ventral process of scapula at the posteroventral margin of glenoid: absent (0); present (1).

Updated codings: *Saichania* from ? to 1; *P. grangeri* from ? to 1. Notes: The scapula is preserved in the holotype specimen of *Saichania*, and a scapula is known in *P. grangeri* referred specimen PIN 614.

1. Form of the scapula acromion process: not developed or ridge-like along the dorsal border of the scapula (0); flange-like and folded over towards the scapula glenoid (1); ridge terminating in a knob-like eminence (2). (Vickaryous et al. [5]: character 52)

Updated codings: *Tianzhenosaurus* from 1 to ?.

1. Orientation of the acromion process of scapula: directed away from the glenoid (0); directed towards scapula glenoid (1). After Kirkland [20].

Updated codings: *Tianzhenosaurus* from 1 to ?.

1. Scapulocoracoid buttress: absent (0); present (1).

Updated codings: *Talarurus* from ? to 0. Notes: See PIN 557.

1. Distal end of scapula shaft: narrow (0); expanded (1). (Sereno [2]: character 20)

Updated codings: *Talarurus* from 1 to 0, *Tianzhenosaurus* from 1 to ?. Notes: In specimen PIN 557, the scapula is narrow distally.

1. Contact between sternal plates: separate (0); fused (1). (Sereno [2]: character 112; Lee [4]: character 60)
2. Separation of humeral head and deltopectoral crest in anterior view: continuous (0); separated by a distinct notch (1).

Updated codings: *Talarurus* from ? to 0. Notes: See PIN 557.

1. Separation of humeral head and medial tubercle in anterior view: continuous (0); separated by a distinct notch (1).

Updated codings: *Talarurus* from ? to 0, *Euoplocephalus* from 1 to 0. Notes: See PIN 557 for *Talarurus*.

1. Ratio of deltopectoral crest length to humeral length: ≤0.5 (0); >0.5 (1).

Updated codings: *Tianzhenosaurus* from 1 to ?.

1. Orientation of deltopectoral crest projection: lateral (0); anterolateral (1). (Sereno [2]: character 113)

Updated codings: *Tianzhenosaurus* from 0 to ?.

1. Shape of the radial condyle of humerus round / proximal end of radius in end-on view: non-circular (0); circular (1).

Updated codings: *Tianzhenosaurus* from 0 to ?.

1. Ratio of the length of metacarpal V to metacarpal III: ≤0.5 (0); >0.5 (1). (Sereno [2]: character 6)

Updated codings: *Tianzhenosaurus* from 1 to ?.

1. Manual digit number: 5 (0); 4 (1); 3 (2).

Updated codings: *Tarchia* from 1 to ?, *Tianzhenosaurus* from 1 to ?. Notes: The manus is unknown for *Tarchia*.

1. Shape of manual and pedal ungual phalanges: claw shaped (0); hoof shaped (1). (Sereno [2]: character 7)

Updated codings: *Dyoplosaurus* from ? to 1, *Edmontonia* from 0 to 1, *Tianzhenosaurus* from 1 to ?. Notes: The pedal unguals are preserved in ROM 784.

1. Length of the preacetabular process of ilium as a percentage of total ilium length: ≤ 50% (0); > 50 %.

Updated codings: *Dyoplosaurus* from 0 to ?, *Tianzhenosaurus* 1 to ?. Notes: The preacetabular process of the ilium of ROM 784 is broken, and so this character cannot be coded for *Dyoplosaurus*.

1. Angle of lateral deflection of the preacetabular process of the ilium: 10º–20º (0); 45º (1). (Sereno [2]: character 21)

Updated codings: *Pinacosaurus mephistocephalus* from 0 to 1; *Dyoplosaurus* from 0 to 1, *Tianzhenosaurus* from 1 to ?.

1. Orientation of the preacetabular portion of the ilium: near vertical (0); near horizontal (1). (Kirkland [20]: character 45)

Updated codings: *Tianzhenosaurus* from 1 to ?.

1. Form of the preacetabular portion of the ilium: straight process (0); pronounced ventral curvature (1).

Updated codings: *Tianzhenosaurus* from 1 to ?.

1. Lateral exposure of the acetabulum: exposed (0) acetabulum partially obscured as it is partially encircled by the distal margin of the ilium (1).

Updated codings: *Pinacosaurus mephistocephalus* from 0 to ?; *Tarchia* from 1 to ?; *Saichania* from 1 to ?, *Tianzhenosaurus* 1 to ?. Notes: The pelvis is not known for *Tarchia or Saichania*, and so this character cannot be coded. The pelvis is preserved in *Pinacosaurus mephistocephalus*, but it cannot be determined from the photographs in Godefroit et al. [8] how this feature should be coded.

1. Perforation of the acetabulum: present, open acetabulum (0); absent, closed acetabulum (1). (Sereno [2]: character 74)

Updated codings: *Tianzhenosaurus* from 1 to ?.

1. Postacetabular ilium length, relative to diameter of acetabulum: greater (0); smaller (1). (Sereno [2]: character 114)

Updated codings: *Pinacosaurus grangeri* from ? to 1, *Tianzhenosaurus* from 1 to ?. Notes: The postacetabular process is shorter than the length of the acetabulum in specimen PIN 614 of *Pinacosaurus grangeri*.

1. Pubis size: large (0); reduced (1). (Kirkland [20]: character 46)

Updated codings: *Euoplocephalus* from 1 to ?, *Tianzhenosaurus* from 1 to ?. Notes: A pubis is unknown for any specimen of *Euoplocephalus*.

1. Prepubic process: present (0); absent (1).

Updated codings: *Euoplocephalus* from 1 to ?, *Tianzhenosaurus* from 1 to ?. Notes: A pubis is unknown for any specimen of *Euoplocephalus*.

1. Structure and rotation of the body of the pubis: gracile without dorsolateral rotation (0); massive and dorsolaterally rotated (1). After Carpenter [18].

Updated codings: *Euoplocephalus* from 1 to ?. Notes: A pubis is unknown for any specimen of *Euoplocephalus*.

1. Size of pubic contribution to acetabulum: over 25 % (0); less than 25 % (1). (Vickaryous et al. [5]: character 58)

Updated codings: *Euoplocephalus*, *Saichania*, *Talarurus*, and *Tarchia*, from 1 to ?. Notes: A pubis is unknown in all of these taxa, and so this character cannot be coded.

1. Shape of ischium: straight (0); ventrally flexed at mid-length (1). (Kirkland [20]: character 37)

Updated codings: *Dyoplosaurus* from ? to 0, *Tianzhenosaurus* from 0 to ?. Notes: Although the shafts are broken at the midlength of each ischium, the ischia of ROM 784 would have been straight.

1. Shape of the dorsal margin of ischium: straight or concave (0); convex (1). (Sereno [2]: character 115)

Updated codings: *Tianzhenosaurus* from 1 to ?.

1. Angle between long axis of femoral head and long axis of shaft: <100º (0); 100º to 120º (1); >120º (2).

Updated codings: *Ankylosaurus* from 1 to 2, *Pinacosaurus grangeri* from 1 to 2, *Euoplocephalus* from 1 to 2. Notes: *Euoplocephalus* estimated from AMNH 5404. *Ankylosaurus* estimated from Carpenter [9].

1. Separation of femoral head from greater trochanter: continuous (0); separated by a distinct notch or change in slope (1).

Updated codings: *Tianzhenosaurus* from 1 to ?.

1. Differentiation of the anterior trochanter of the femur: separated from femoral shaft by a deep groove laterally and dorsally (0); fused to femoral shaft (1). (Kirkland [20]: character 36)

Updated codings: *Tianzhenosaurus* from 1 to ?.

1. Oblique ridge on lateral femoral shaft, distal to anterior trochanter: absent (0); present (1).

Updated codings: *Dyoplosaurus* from ? to 0. Notes: the femur is preserved in ROM 784, so this character can be coded.

1. Form of the fourth trochanter: pendant (0); ridge-like (1). (Sereno [2]: character 24)

Updated codings: *Dyoplosaurus* from ? to 1, *Tianzhenosaurus* from 1 to ?. Notes: the femur is preserved in ROM 784, so this character can be coded.

1. Location of the fourth trochanter on the femoral shaft: proximal (0) distal, over half-way down the femoral shaft (1).

Updated codings: *Dyoplosaurus* from ? to 1, *Tianzhenosaurus* from 1 to ?. Notes: the femur is preserved in ROM 784, so this character can be coded.

1. Maximum distal width of the tibia, compared to the maximum proximal width: narrower (0); wider (1). (Sereno [2]: character 188)

Updated codings: *Dyoplosaurus* from ? to 1, *Tianzhenosaurus* from 1 to ?. Notes: the tibia is preserved in ROM 784, so this character can be coded.

1. Contact between tibia and astragalus: articulated (0); fused, with suture obliterated (1).

Updated codings: *Tianzhenosaurus* from 1 to ?.

1. Number of pedal digits: 5 (0); 4 (1); 3 (2).

Updated codings: *P. grangeri* from ? to 2, *Tianzhenosaurus* from 1 to ?. Notes: See description of *Pinacosaurus* manual and pedal elements in Currie et al. [21].

1. Phalangeal number in pedal digit IV: 5 (0); ≤4 (1). (Sereno [2]: character 26)
2. Parasagittal row of keeled osteoderms situated on the dorsal aspect of the trunk: absent (0); present (1). (Sereno [2]: character 2)

Updated codings: *Pinacosaurus mephistocephalu*s from ? to 1; *Dyoplosaurus* from ? to 1. Notes: *in situ* osteoderms are preserved in the holotypes of both of these taxa (cervical half rings are osteodermal elements).

1. Large, laterally compressed plates on the dorsal aspect of the trunk: absent (0); present (1).
2. Lateral rows of osteoderms on the dorsal aspect of the trunk: absent (0); present (1). (Sereno [2]: character 3)
3. Number of distinct cervical pectoral bands: none (0); one (1); two (2). (Kirkland [20]: character 38)

Updated codings: changed all ankylosaurids to 2 where both cervical half rings are known. *Tarchia* from 1 to ?. Notes: No cervical half rings are preserved with diagnostic *Tarchia* material.

1. Form of the cervical bands: separate at the midline, forming pairs of quarter rings (0); fused at the midline, forming half rings (1).

Updated codings: *Ankylosaurus magniventris* from 0 to 1. Notes: Firsthand examination of *Ankylosaurus magniventris* AMNH 5895 indicates that the two cervical ring fragments thought to go together by Carpenter [9] do not fit together. Instead, these represent fragments from the first and second cervical rings. Because they do not fit together, there is no reason to assume that *Ankylosaurus magniventris* had ‘quarter rings’, rather than the typical semicircular half rings found in all other ankylosaurids.

1. Pectoral spikes: absent (0); present (1). After Kirkland [20].
2. Form of pectoral spikes: no grooves and a solid base (0); posterior groove with a hollow base (1).
3. Sacral shield of fused osteoderms: absent (0); present (1). (Kirkland [20]: character 42)

Updated codings: *Euoplocephalus* from 0 to ?, *Dyoplosaurus* from 0 to ?, *Edmontonia* 1 to ?, *Saichania* from 0 to ?. Notes: There are no specimens that preserve *in situ* sacral osteoderms for *Euoplocephalus*, *Edmontonia*, or *Saichania*. *Dyoplosaurus* preserves osteoderms lateral to the ilia, but not on the dorsal surface.

1. Form of ossicles in sacral armour: irregular ossicles (0); sub-hexagonal ossicles of similar sizes (1).

Updated codings: *Euoplocephalus* from 0 to ?. Notes: There are no specimens that preserve *in situ* sacral osteoderms for *Euoplocephalus*.

1. Size of lateral trunk plates, sacral plates and caudal plates: small (0); large and hollow based (1).

Updated codings: *Euoplocephalus* from 1 to ?, *Tianzhenosaurus* from 1 to ?. Notes: There are no specimens that preserve *in situ* lateral trunk plates, sacral plates, and caudal plates for *Euoplocephalus*.

1. Form of caudal plate: little dorsal projection (0); tall with thin dorsal extremity (1).

Updated codings: *Euoplocephalus* from 0 to ?, *Tianzhenosaurus* from 0 to ?. Notes: There are no specimens that preserve *in situ* caudal osteoderms for *Euoplocephalus*.

1. Tail club: absent (0); present (1). (Kirkland [20]: character 44)
2. New character (Analysis 3 only): Small (<2 cm diameter), circular caputegulae posterolateral to orbit, along ventral edge of squamosal horn and/or along dorsal edge of quadratojugal horns: absent (0); present (1)
3. New character (Analysis 3 only): Cervical half rings: composed of osteoderms that are either tightly adjacent to one another or coossified at the edges, forming arc over the cervical region (0), composed of osteoderms and underlying bony band segments, osteoderms may or may not coossify to the band, forming arc over the cervical region (1).
4. New character (Analysis 3 only): Composition of first cervical half ring: first cervical half ring has 4 to 6 primary osteoderms only (0), first cervical half ring has 4 to 6 primary osteoderms surrounded by small (<2 cm diameter) circular secondary osteoderms.
5. New character (Analysis 3 only): Form of caudal osteoderms: dorsoventrally compressed, triangular in dorsal view (0), or low cones (1).
6. New character (Analysis 3 only): Tail club knob shape: major knob osteoderms semicircular in dorsal view (1), triangular in dorsal view (2).
7. New character (Analysis 3 only): Tail club knob proportions: tail club knob length > width (1), length = width (2), width > length (3).
8. Thompson RS, Parish JC, Maidment SCR, Barrett PM (2012) Phylogeny of the ankylosaurian dinosaurs (Ornithischia: Thyreophora). J Syst Palaeontol 10:301-312.
9. Sereno PC (1999) The evolution of dinosaurs. Science 284:2137-2147.
10. Carpenter K, Miles C, Cloward K (1998) Skull of a Jurassic ankylosaur (Dinosauria). Nature 393:782-783.
11. Lee Y-N (1996) A new nodosaurid ankylosaur (Dinosauria: Ornithischia) from the Paw Paw Formation (Late Albian) of Texas. J Vertebr Paleontol 16:232-245.
12. Vickaryous MK, Maryańska T, Weishampel DB (2004) Ankylosauria. In: Russell DB, Dodson P, Osmólska H, editors. The Dinosauria, 2nd Edition. Berkeley: University of California Press, pp. 363–392.
13. Pang Q, Cheng Z (1998) A new ankylosaur of Late Cretaceous from Tianzhen, Shanxi. Prog Nat Sci 8:326-334.
14. Hill RV, Witmer LW, Norell MA (2003) A new specimen of *Pinacosaurus grangeri* (Dinosauria: Ornithischia) from the Late Cretaceous of Mongolia: ontogeny and phylogeny of ankylosaurs. Am Mus Novit 3395:1-29.
15. Godefroit P, Pereda−Suberbiola X, Li H, Dong Z (1999) A new species of the ankylosaurid dinosaur *Pinacosaurus* from the Late Cretaceous of Inner Mongolia (P.R. China). Bull Inst R Sc N B-S 69: 17–36.
16. Carpenter K (2004) Redescription of *Ankylosaurus magniventris* Brown, 1908 (Ankylosauridae) from the Upper Cretaceous of the Western Interior of North America. Can J Earth Sci 41:961-986.
17. Arbour VM, Burns ME, Sissons RL (2009) A redescription of the ankylosaurid dinosaur *Dyoplosaurus acutosquameus* Parks, 1924 (Ornithischia: Ankylosauria) and a revision of the genus. J Vertebr Paleontol 29:1117-1135.
18. Carpenter K, Kirkland JI, Burge D, Bird J (1999) Ankylosaurs (Ankylosauria: Ornithischia) of the Cedar Mountain Formation, Utah, and their stratigraphic distribution. In: Gillette DD, editor. Vertebrate paleontology in Utah, Utah Geological Survey Miscellaneous Publications 9. Salt Lake City: Utah Geological Survey, pp. 243-251.
19. Tumanova TA (1993) [A new armored dinosaur from southeastern Gobi]. Paleontol Zh 3:92-98. [In Russian]
20. Sullivan RM (1999) *Nodocephalosaurus kirtlandensis*, gen. et sp. nov., a new ankylosaurid dinosaur (Ornithischia: Ankylosauria) from the Upper Cretaceous Kirtland Formation (Upper Campanian), San Juan Basin, New Mexico. J Vertebr Paleontol 19:126-139.
21. Kirkland JI, Carpenter K, Hunt AP, Scheetz RD (1998) Ankylosaur (Dinosauria) specimens from the Upper Jurassic Morrison Formation. Mod Geol 23:145-177.
22. Carpenter K (1990) Ankylosaur systematics: an example using *Panoplosaurus* and *Edmontonia* (Ankylosauria: Nodosauridae). In: Carpenter K, Currie PJ, editors. Dinosaur systematics: perspectives and approaches. Cambridge: Cambridge University Press, pp. 281-297.
23. Carpenter K, Hayashi S, Kobayashi Y, Maryańska T, Barsbold R, et al. (2011) *Saichania chulsanensis* (Ornithischia, Ankylosauridae) from the Upper Cretaceous of Mongolia. Palaeontogr Abt A 293:1-61.
24. Maryańska T (1977) Ankylosauridae (Dinosauria) from Mongolia. Palaeontologia Polonica 37:85–151.
25. Carpenter K (2001) Phylogenetic analysis of the Ankylosauria. In: Carpenter K, editor. The armored dinosaurs. Bloomington: Indiana University Press, pp. 455-483.
26. Sullivan RM, Fowler DW (2006) New specimens of the rare ankylosaurid dinosaur *Nodocephalosaurus kirtlandensis* (Ornithischia: Ankylosauridae) from the Upper Cretaceous Kirtland Formation (De-na-zin Member), San Juan Basin, New Mexico. New Mexico Museum of Natural History and Science Bulletin 35:259-261.
27. Kirkland JI (1998) A polacanthine ankylosaur (Ornithischia: Dinosauria) from the Early Cretaceous (Barremian) of eastern Utah. New Mexico Museum of Natural History and Science Bulletin 14:271-281.
28. Currie PJ, Badamgarav D, Koppelhus EB, Sissons R, Vickaryous MK (2011) Hands, feet, and behavior in *Pinacosaurus* (Dinosauria: Ankylosauridae). Acta Palaeontol Pol 56:489-504.
